# Longitudinal Trajectories of Physical and Mental Health-Related Quality of Life Following Psychobiotic Supplementation in Gastrointestinal Oncology Patients: A Post Hoc Analysis of the ProDeCa Study

**DOI:** 10.3390/nu18193254

**Published:** 2026-10-02

**Authors:** Georgios G. Tzikos, Alexandra-Eleftheria Menni, Ioannis M. Theodorou, Helen Theodorou, Joulia K. Tsetis, Eleni Chamalidou, George Stavrou, Anne D. Shrewsbury, Amalia Bolanou, Aristeidis Ioannidis, Katerina Kotzampassi

**Affiliations:** 1 Department of Surgery, Aristotle University of Thessaloniki, 54636 Thessaloniki, Greece; tzikos_giorgos@gmail.com (G.G.T.); alexandra.menni@gmail.com (A.-E.M.); a_shrewsbury@yahoo.com (A.D.S.); ariioann@yahoo.gr (A.I.); 2 Hellenic Institute for the Study of Sepsis (HISS), 11528 Athens, Greece; itheodorou@med.uoa.gr (I.M.T.); amalia.mpolanou.ab@gmail.com (A.B.); 3 Department of Sociology, School of Social Sciences, University of Crete, 74100 Rethymno, Greece; psy5057@psy.soc.uoc.gr; 4 Uni-Pharma S.A., 14564 Athens, Greece; jtsetis@uni-pharma.gr; 5 Outpatient Surgical Oncology Unit, Chemotherapy Department, AHEPA University Hospital, Aristotle University of Thessaloniki, 54636 Thessaloniki, Greece; eleniham@gmail.com; 6 Department of Surgery, 417 NIMTS (Army Share Fund Hospital), 11521 Athens, Greece; stavgd@gmail.com

**Keywords:** psychobiotics, HR-QoL, surgical oncology, chemotherapy, gastrointestinal cancer, gut–brain axis, post hoc analysis, SF-36 questionnaire, adjunctive therapy

## Abstract

Background/Objectives: Patients undergoing surgery followed by adjuvant chemotherapy for gastrointestinal cancer frequently experience impaired health-related quality of life (HR-QoL). This post hoc analysis of the ProDeCa randomized trial investigated whether psychobiotic treatment was associated with longitudinal changes in HR-QoL. Methods: Participants in the randomized, double-blind, placebo-controlled ProDeCa trial (NCT06496438) received either a four-strain psychobiotic formulation or placebo for four weeks during adjuvant chemotherapy. HR-QoL was assessed using the Short Form-36 (SF-36) questionnaire at baseline (V0), treatment completion (V1), and two months later (V2). Longitudinal changes in the eight SF-36 domains and the Physical (PCS) and Mental (MCS) Component Summary scores were analyzed using mixed models for repeated measures. Results: The present analysis included 266 participants, with HR-QoL data available for 231–232 participants at V2, depending on the SF-36 outcome. Significant group-by-visit interactions were observed across the eight SF-36 domains (all *p* < 0.001), indicating divergent longitudinal HR-QoL trajectories between treatment groups. Visit-specific adjusted comparisons generally favored psychobiotics; however, between-group differences in Vitality and Mental Health were not statistically significant at V1 and became significant at V2. At V2, the adjusted LS mean between-group differences were 16.79 points for PCS (95% CI, 15.69–17.88) and 9.66 points for MCS (95% CI, 8.49–10.84). Exploratory subgroup analyses did not provide evidence of differential treatment effects based on formal interaction tests. Conclusions: Psychobiotic treatment was associated with more favorable longitudinal HR-QoL outcomes during adjuvant chemotherapy after gastrointestinal cancer surgery. These findings support further investigation of psychobiotics as an adjunctive strategy for HR-QoL support in larger multicenter randomized trials.

## 1. Introduction

Health-related quality of life (HR-QoL) has become a key outcome in contemporary oncology, particularly among patients undergoing adjuvant chemotherapy following radical gastrointestinal cancer surgery, where treatment-related physical and psychological burdens substantially affect daily functioning. Accordingly, large oncological trials increasingly incorporate validated patient-reported outcome measures alongside conventional efficacy endpoints, including tumor response and survival, to provide a more comprehensive assessment of treatment impact from the patient’s perspective. Moreover, pooled analyses across different malignancies have consistently demonstrated that impaired baseline patient-reported functioning is associated with poorer survival outcomes, underscoring the prognostic significance of HR-QoL and its importance in balancing treatment efficacy with patients’ overall well-being and quality of life [1,2,3,4].

Gastrointestinal malignancies represent a particularly challenging clinical setting because HR-QoL may be compromised by both the underlying disease and the cumulative effects of multimodal treatment [5]. Advanced age and reduced physiological reserve may further delay postoperative recovery and functional restoration [6]. Extensive upper gastrointestinal resections are frequently associated with persistent dysphagia, impaired digestive function, nutritional deficiencies, weight loss, and sarcopenia [7,8]. Likewise, colorectal surgery may lead to long-term alterations in bowel function, particularly diarrhea, while the presence of a temporary or permanent stoma may impose additional psychosocial burdens, including concerns regarding leakage, odor, altered body image, and sexual dysfunction, all of which may substantially delay recovery of HR-QoL [9,10,11,12].

Although radical surgical resection remains the cornerstone of curative treatment for gastrointestinal malignancies [13], a substantial proportion of patients subsequently require adjuvant chemotherapy to reduce the risk of disease recurrence. Despite its well-established survival benefits, chemotherapy is frequently accompanied by fatigue, pain, peripheral neuropathy, mucositis, diarrhea, immunosuppression, and psychological distress. These treatment-related toxicities often result in persistent physical limitations and emotional disturbances, including anxiety and depression, thereby adversely affecting multiple domains of HR-QoL, with some impairments persisting well beyond treatment completion [10,14,15,16,17,18,19,20].

Identifying patients at increased risk of compromised HR-QoL has therefore become an important component of supportive cancer care. A recent multicentre cross-sectional study of colorectal cancer patients receiving outpatient chemotherapy demonstrated that both clinical and sociodemographic characteristics—including the presence of a colostomy, comorbidity burden, unemployment, female sex, rural residence, and lack of home ownership—were independently associated with poorer outcomes across several SF-36 domains [9]. These findings highlight the multifactorial determinants of HR-QoL and emphasize the need for interventions targeting not only tumor control but also patients’ physical, psychological, and social well-being.

Growing evidence supports the role of the gut–brain axis, a bidirectional communication network linking the intestinal microbiota with the central nervous system, in regulating both mental health and physical resilience [21,22]. Within this framework, psychobiotics have emerged as a promising therapeutic approach. These probiotics are defined by their ability to exert beneficial effects on mental health through neuroactive, metabolic, and immunomodulatory mechanisms [23,24]. Proposed mechanisms include modulation of the hypothalamic–pituitary–adrenal (HPA) axis, attenuation of systemic and neuroinflammation, production of neuroactive metabolites, regulation of vagal signaling, and reinforcement of intestinal barrier integrity [14,17,25,26,27,28,29]. Accumulating preclinical and clinical evidence suggests that these mechanisms may alleviate depression, anxiety, and perceived stress, improve cognitive function, and reduce systemic inflammation [24,27,28,30], providing a strong biological rationale for investigating psychobiotics as a supportive strategy to improve both psychological outcomes and HR-QoL in patients with cancer [31,32,33,34].

In the recently published ProDeCa randomized clinical trial involving patients undergoing gastrointestinal cancer surgery followed by adjuvant chemotherapy, treatment with a defined four-strain psychobiotic formulation (*Bifidobacterium animalis* subsp. *lactis* LMG P-21384, *Bifidobacterium breve* DSM 16604, *Bifidobacterium longum* DSM 16603, and *Lacticaseibacillus rhamnosus* ATCC 53103) significantly improved symptoms of depression, anxiety, perceived stress, insomnia, and pain [35,36]. Given the close interrelationship between psychological well-being, physical functioning, and perceived quality of life, these findings raised the clinically relevant question of whether the observed benefits extended beyond individual psychological symptoms to broader dimensions of HR-QoL. Accordingly, the present post hoc analysis of the ProDeCa trial was designed to investigate the longitudinal effects of psychobiotic treatment on HR-QoL in patients receiving adjuvant chemotherapy after gastrointestinal cancer surgery. HR-QoL was assessed using the Short Form-36 (SF-36) questionnaire, and longitudinal changes were examined across all eight SF-36 domains as well as the Physical and Mental Component Summary scores. We hypothesized that psychobiotic treatment would be associated with more favorable HR-QoL trajectories over time than placebo after adjustment for baseline score and clinically relevant baseline covariates, including gender, baseline depression status, baseline pain status, and tumor location.

## 2. Materials and Methods

### 2.1. Study Design and Participants

This post hoc secondary analysis was conducted within the framework of the ProDeCa randomized clinical trial (ClinicalTrials.gov Identifier: NCT06496438, registered on 11 July 2024). HR-QoL assessment using the SF-36 was included in the original trial protocol and was prospectively collected from participants receiving psychobiotic or placebo treatment during adjuvant chemotherapy following gastrointestinal cancer surgery. The specific longitudinal and subgroup analyses reported in the present study were subsequently undertaken post hoc [35,36].

Eligible participants were adults (≥18 years) undergoing adjuvant chemotherapy after radical resection for gastrointestinal cancer who were cognitively able to provide written informed consent. Patients with active psychiatric disorders, a history of suicide attempt, current antidepressant treatment, recent antibiotic or probiotic use (within four weeks before enrolment), or severe immunosuppression were excluded from the study. Recruitment began in November 2023, and participant eligibility assessment was completed in October 2024.

As previously reported in the primary ProDeCa publication, 295 participants were randomized (146 to psychobiotics and 149 to placebo). During the four-week intervention period, three participants died and 26 did not meet the protocol-defined adherence threshold (>90% of prescribed doses). Accordingly, 266 participants (132 psychobiotic and 134 placebo) who completed at least the baseline (V0) and post-treatment (V1) assessments constituted the analytical population for the present post hoc analysis. Because post-randomization exclusions occurred, this population does not represent a strict intention-to-treat cohort. The complete participant flow has been reported in the primary ProDeCa publication [35,36]. Detailed baseline and clinical characteristics of the ProDeCa cohort have been reported previously [35]. Tumor location, disease stage, Charlson Comorbidity Index, and selected chemotherapy-related adverse effects and treatment interruptions were comparable between the psychobiotic and placebo groups.

All participants received standard adjuvant chemotherapy according to institutional protocols, consisting of either the FOLFOX regimen (5-fluorouracil, leucovorin, and oxaliplatin) or the FOLFIRI regimen (5-fluorouracil, leucovorin, and irinotecan). The choice of chemotherapy regimen was made independently of study allocation and therefore had no influence on treatment assignment to the psychobiotic or placebo group [35]. Pain management followed the World Health Organization analgesic ladder. Most participants received step-2 analgesia, consisting of weak opioids (codeine or tramadol) combined with paracetamol or tramadol alone at daily doses of up to 300 mg. Nine patients with pancreatic cancer additionally received tapentadol under the supervision of the Chronic Pain Management Unit of our University Hospital [36].

### 2.2. Intervention Protocol

Participants randomly assigned to the intervention group received the psychobiotic regimen twice daily for four consecutive weeks, corresponding to a total daily dose of 1.76 × 10^11^ CFU [35,36]. The formulation contained the following four bacterial strains at the specified doses: *Bifidobacterium animalis* subsp. *lactis* LMG P-21384 [BS01] [2.50 × 10^10^ cfu/dose], *Bifidobacterium breve* DSM 16604 [BR03] [1.00 × 10^10^ cfu/dose], *Bifidobacterium longum* DSM 16603 [BL04] [8.00 × 10^9^ cfu/dose], and *Lacticaseibacillus rhamnosus* ATCC 53103 [GG] [4.50 × 10^10^ cfu/dose]. The investigational preparation used in the ProDeCa trial contained only the four bacterial strains specified above and did not contain saffron or magnesium, thus differing in this respect from the commercially available formulation referred to in the trial registry. Participants allocated to the placebo group received sachets containing sorbitol, steviol glycosides, polyethylene glycol 6000, and lemon-grapefruit flavors. The sachets were identical to the psychobiotic formulation in packaging, taste, smell, appearance, consistency, and solubility in drinking water, thereby maintaining blinding throughout the intervention period. Compliance in both treatment groups was assessed by sachet counts at the end of the four-week treatment period.

### 2.3. Health-Related Quality of Life [HR-QoL]

Health-related quality of life (HR-QoL) was assessed in all participants at baseline before initiation of the study intervention (V0), immediately after completion of the four-week treatment period (V1), and two months later (V2) using the validated Greek version of the 36-Item Short Form Health Survey (SF-36), one of the most widely used generic instruments for assessing HR-QoL across a broad spectrum of diseases, including cancer [6,12,37,38].

The SF-36 is a self-administered questionnaire comprising 36 items that evaluate two principal dimensions of health—physical and mental well-being—through eight multi-item domains: Physical Functioning, Role Physical, Bodily Pain, General Health, Vitality, Social Functioning, Role Emotional, and Mental Health. Physical Functioning assesses limitations in performing everyday physical activities; Role Physical evaluates the extent to which physical health interferes with work and other daily activities; and Bodily Pain measures both pain intensity and its impact on normal functioning. General Health and Vitality provide an overall assessment of perceived health status and energy levels, respectively. The remaining three domains—Social Functioning, Role Emotional, and Mental Health—reflect the psychosocial aspects of health, including social participation, role limitations attributable to emotional problems, and overall psychological well-being. In addition, the Physical Component Summary (PCS) and Mental Component Summary (MCS) scores were calculated to provide global measures of physical and mental health status. Scores for each of the eight SF-36 domains were transformed to a 0–100 scale, with higher scores indicating better perceived health status and functioning. The Physical Component Summary (PCS) and Mental Component Summary (MCS) scores were calculated separately as composite summary measures according to the SF-36 scoring methodology [39,40,41,42,43,44].

### 2.4. HR-QoL in Relation to Depression, Pain, Tumor Location and Gender

**Figure 1:**
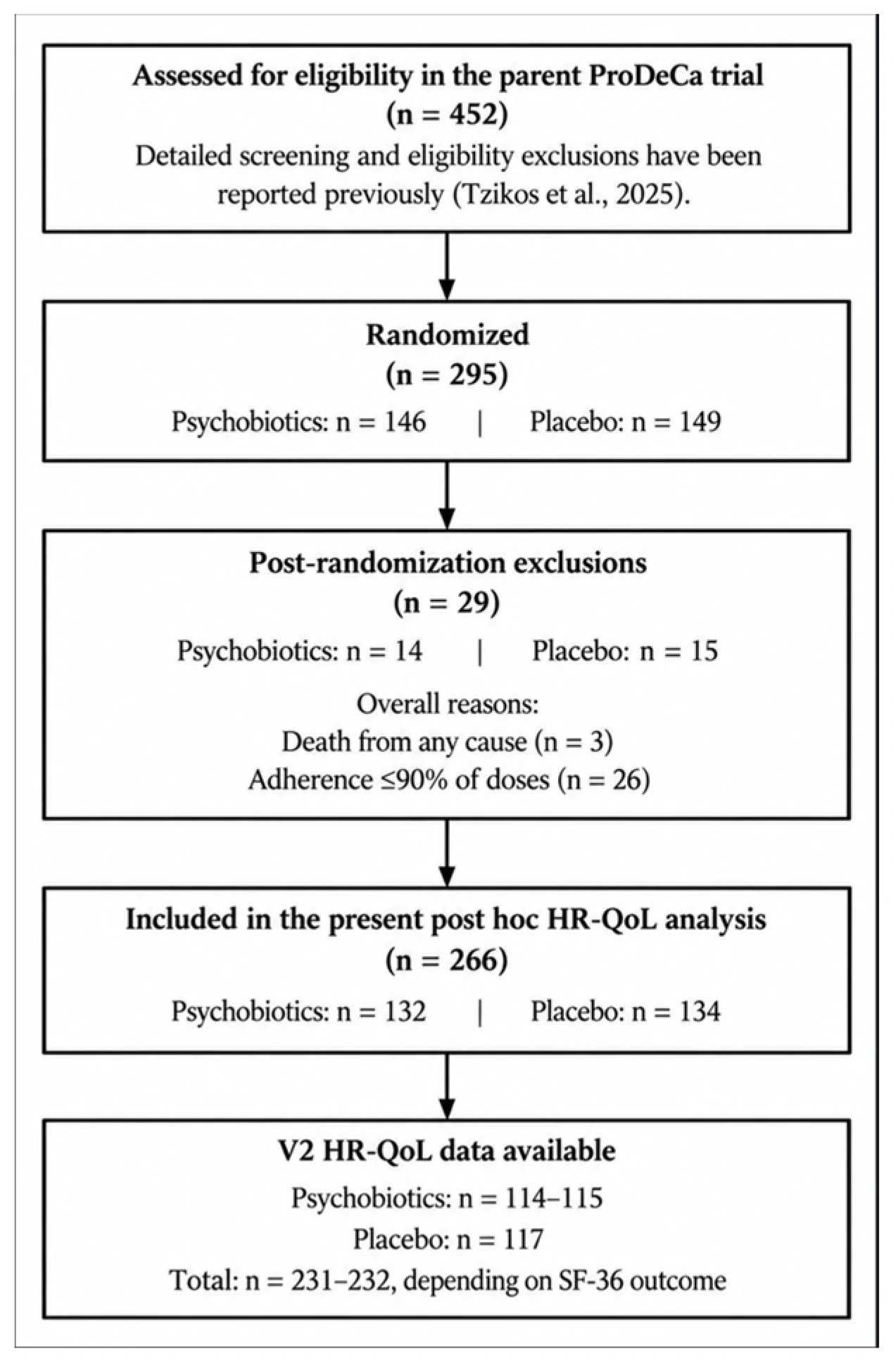
Participant flow from the parent ProDeCa randomized trial to the analytical population included in the present post hoc HR-QoL analysis [35]. V2 sample size varied slightly across SF-36 outcomes because of outcome-specific missing data.

Although participants in the two treatment groups were comparable with respect to their baseline anthropometric and demographic characteristics, previous analyses of the ProDeCa trial demonstrated significant differences in depression and pain status that could potentially influence HR-QoL outcomes [35,36]. In addition, gender and tumor location (upper or lower gastrointestinal tract) were considered clinically relevant variables that might be associated with patients’ perceived quality of life. Therefore, exploratory post hoc subgroup analyses were performed according to depression status, pain status, gender, and tumor location, while maintaining treatment allocation (psychobiotics or placebo) as the primary comparison factor. Depression was assessed using the 17-item Hamilton Depression Rating Scale (HDRS-D-17) [35], and pain using the Short-Form McGill Pain Questionnaire (MPQ-SF) [36], whereas gender and tumor location were obtained from the baseline demographic and clinical data. The overall analytical design and subgroup classification of the present post hoc analysis are presented in Figure 1.

### 2.5. Statistical Analysis

The primary objective of this post hoc analysis was to evaluate longitudinal changes in health-related quality of life (HR-QoL) between patients receiving psychobiotic treatment and those receiving placebo during adjuvant chemotherapy. Analyses were performed separately for each of the eight SF-36 domains, as well as for the Physical Component Summary (PCS) and Mental Component Summary (MCS) scores. Continuous variables are presented as mean ± standard deviation (SD) or median with interquartile range (IQR), as appropriate, whereas categorical variables are expressed as frequencies and percentages. Participant numbers across the examined baseline subgroup categories are provided in Table S3.12 (Supplementary Materials). Baseline comparisons between treatment groups were performed using the independent-samples *t*-test or the Mann–Whitney U test for continuous variables, according to data distribution, and the χ^2^ test or Fisher’s exact test for categorical variables.

Longitudinal changes from baseline were analyzed using a Mixed Model for Repeated Measures (MMRM), which accounts for within-subject correlations and permits the inclusion of participants with incomplete follow-up under the assumption that missing observations were missing at random (MAR). The dependent variable was the absolute change from baseline for each SF-36 domain and for the PCS and MCS summary scores. Each model included treatment group (psychobiotics or placebo), study visit (Visit 1—V1 and Visit 2—V2), the corresponding baseline score, and the treatment-by-visit interaction as fixed effects. Clinically relevant baseline covariates, including gender, baseline depression status, baseline pain status, and tumor location, were included as covariates. An interaction between baseline score and study visit was also incorporated into the model. Within-subject correlations arising from repeated measurements were modelled using an unstructured covariance matrix.

Adjusted least-squares (LS) mean changes from baseline, together with their 95% confidence intervals (95% CIs), were estimated for each treatment group at each follow-up visit. Adjusted between-group differences in LS mean changes were subsequently estimated for V1 and V2. Exploratory post hoc subgroup analyses according to gender, depression status, pain status, and tumor location were performed to examine the consistency of the observed between-group patterns across clinically relevant patient subgroups. These analyses were considered exploratory and hypothesis-generating and were interpreted accordingly.

Given the post hoc and exploratory nature of the analyses, no formal adjustment for multiple comparisons was applied. Accordingly, individual *p*-values were interpreted cautiously and were not considered confirmatory, particularly for subgroup analyses. No statistically significant interactions between treatment group and the examined baseline subgroup characteristics were observed; accordingly, subgroup findings were considered exploratory and hypothesis-generating and were not interpreted as evidence of treatment-effect modification. Estimates from highly stratified subgroups with small sample sizes were interpreted cautiously and were not considered evidence of differential treatment effects. The large number of HR-QoL outcomes, repeated assessments, and exploratory subgroup comparisons, without formal multiplicity adjustment, increases the possibility of type I error. Accordingly, individual *p*-values, particularly those derived from subgroup analyses, should be interpreted cautiously and the subgroup findings should be regarded as hypothesis-generating.

All statistical tests were two-sided, and a *p*-value < 0.05 was considered statistically significant. Statistical analyses were performed using R software (version 4.5.0).

## 3. Results

A post hoc analysis was performed using data from participants enrolled in the ProDeCa randomized clinical trial to evaluate the effects of psychobiotic treatment on health-related quality of life (HR-QoL) during adjuvant chemotherapy following gastrointestinal cancer surgery. HR-QoL was assessed using the Short Form-36 (SF-36) questionnaire at baseline (V0), at the completion of the four-week intervention (V1), and two months later (V2). In addition to the eight SF-36 domains, the Physical Component Summary (PCS) and Mental Component Summary (MCS) scores were calculated to provide overall measures of physical and mental health status [43,44].

A total of 266 participants were included in the analysis, comprising 132 patients allocated to the psychobiotic group and 134 to the placebo group at baseline. All participants completed the baseline (V0) and post-intervention (V1) assessments. At the final follow-up (V2), HR-QoL data were available for 231–232 participants, depending on the SF-36 outcome, comprising 114–115 participants in the psychobiotic group and 117 in the placebo group. Longitudinal analyses were performed using mixed models for repeated measures, allowing all available observations to contribute to the estimation of treatment effects despite incomplete follow-up.

Baseline SF-36 scores varied between treatment groups across several domains, with the most pronounced differences observed for Physical Functioning, Bodily Pain, Vitality, and Mental Health. Complete baseline descriptive data for all eight SF-36 domains and the PCS and MCS summary scores are provided in Table S3.11. These baseline differences were accounted for in the longitudinal MMRM analyses by inclusion of the corresponding baseline domain or summary score, together with a baseline-by-visit interaction.

### 3.1. Physical Function [PF]

**Figure 2:**
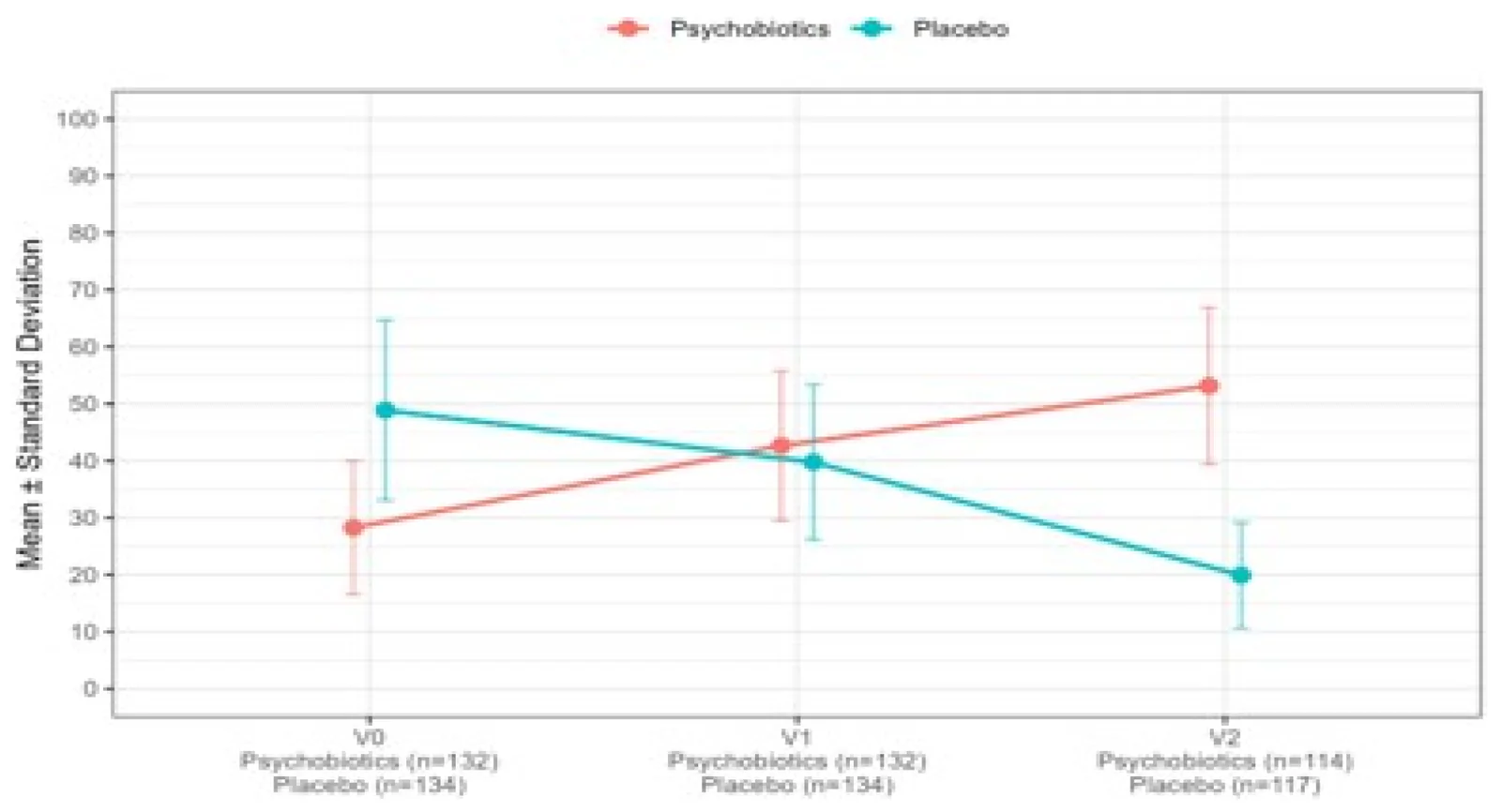
Physical Function [PF] by Visit and Group.

At V0, the mean ± SD PF score was 28.26 ± 11.71 in the psychobiotics group and 48.88 ± 15.80 in the placebo group. Within the psychobiotics group, the mean ± SD change from V0 to V1 was 14.39 ± 11.04 and that from V0 to V2 was 24.78 ± 10.96, whereas the corresponding changes in the placebo group were −9.10 ± 15.14 and −28.89 ± 13.84, respectively (Supplementary Table S1.1; Figure 2). Longitudinal analysis showed a significant group-by-visit interaction for PF (*p* < 0.001; Supplementary Table S2.1).Interactions between baseline characteristics and treatment group were not statistically significant.

**Figure 3:**
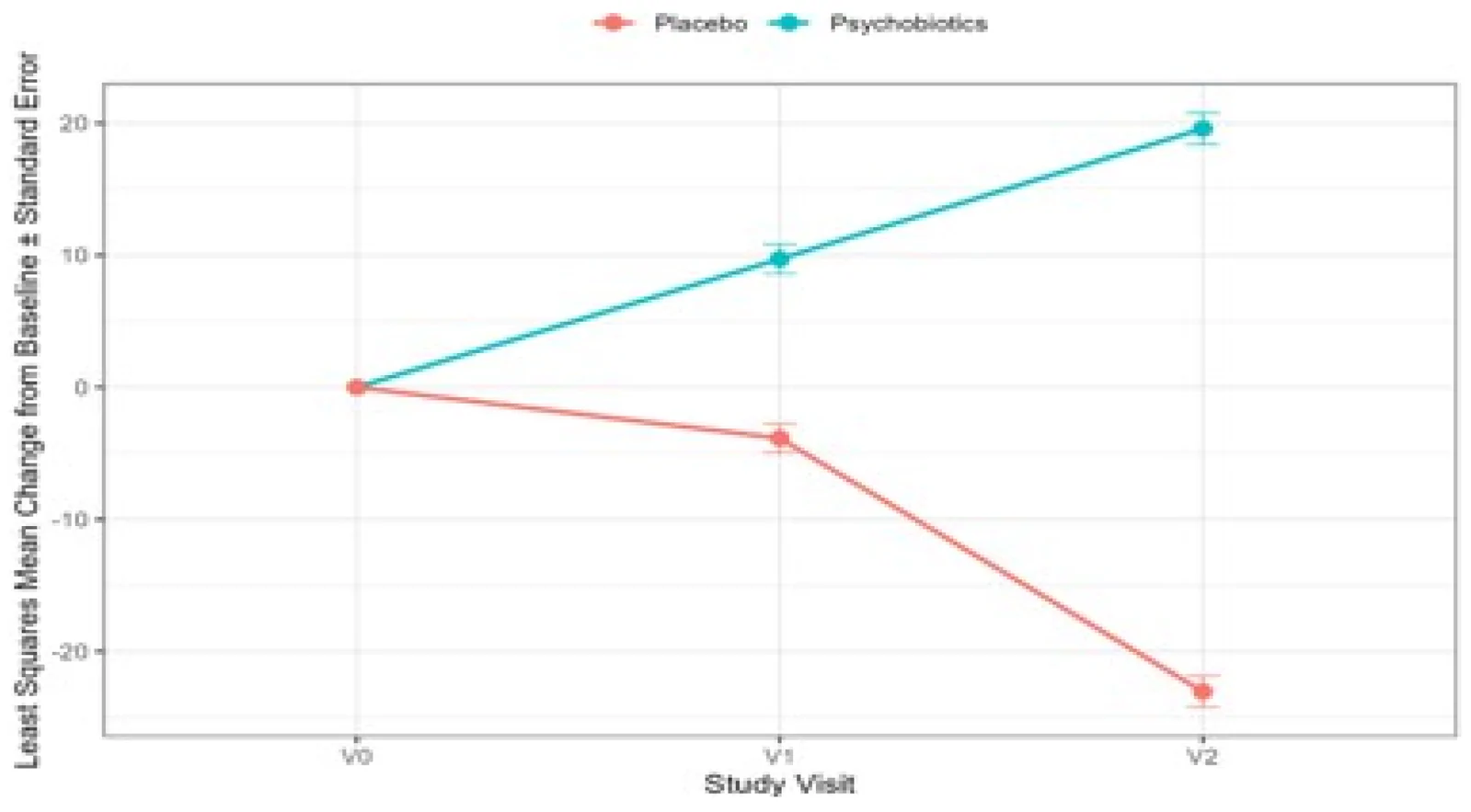
Least Squares Mean Change from Baseline in Physical Function [PF].

At V1, participants receiving psychobiotics demonstrated a higher adjusted LS mean change in PF than those receiving placebo by 13.58 points (95% CI: 10.33–16.83; *p* < 0.001). At V2, the corresponding difference was 42.64 points (95% CI: 39.18–46.11; *p* < 0.001) (Table 1 and Figure 3). Exploratory adjusted subgroup estimates are presented in Supplementary Table S3.1.

### 3.2. Role Physical [RP]

**Figure 4:**
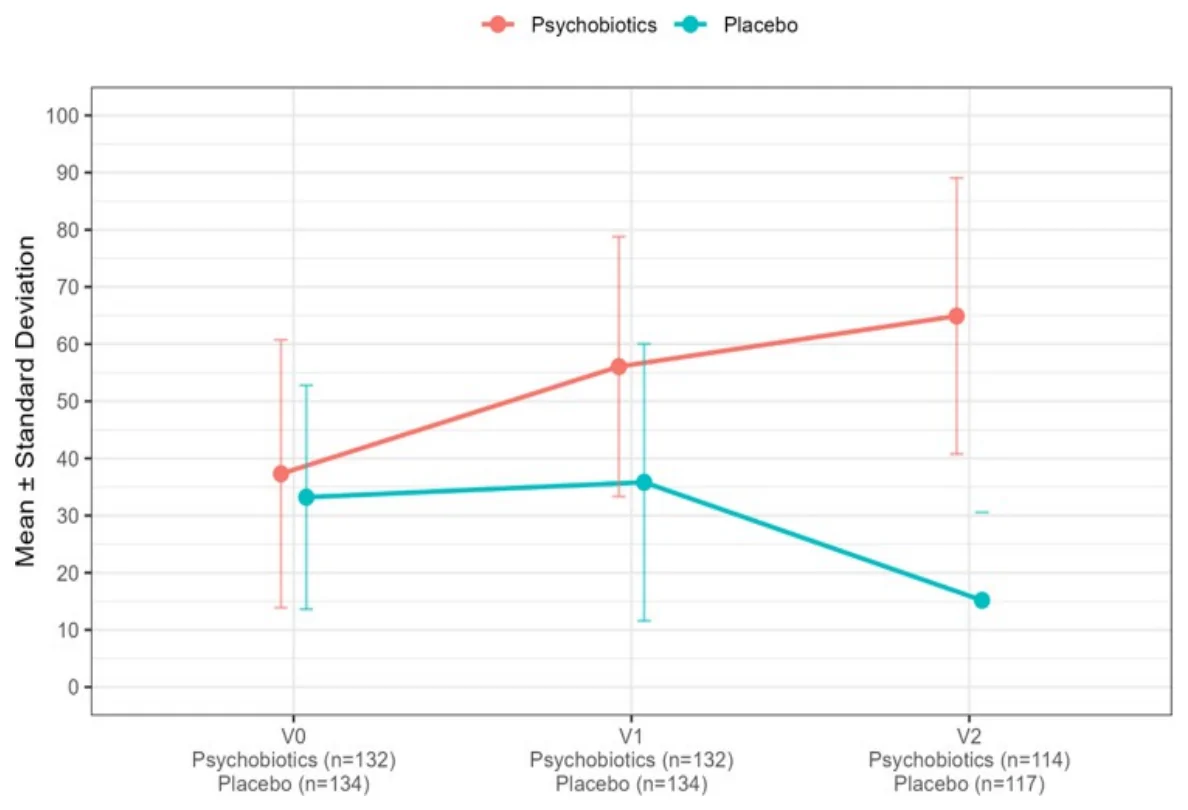
Role Physical [RP] by Visit and Group.

At baseline (V0), the mean ± SD RP score was 37.31 ± 23.42 in the psychobiotics group and 33.21 ± 19.58 in the placebo group. Within the psychobiotics group, the mean ± SD change from V0 to V1 was 18.75 ± 16.46 and that from V0 to V2 was 28.29 ± 18.37, whereas the corresponding changes in the placebo group were 2.61 ± 29.76 and −17.74 ± 19.98, respectively (Supplementary Table S1.2; Figure 4). Longitudinal analysis showed a significant group-by-visit interaction for RP (*p* < 0.001; Supplementary Table S2.2). Interactions between baseline characteristics and treatment group were not statistically significant.

**Figure 5:**
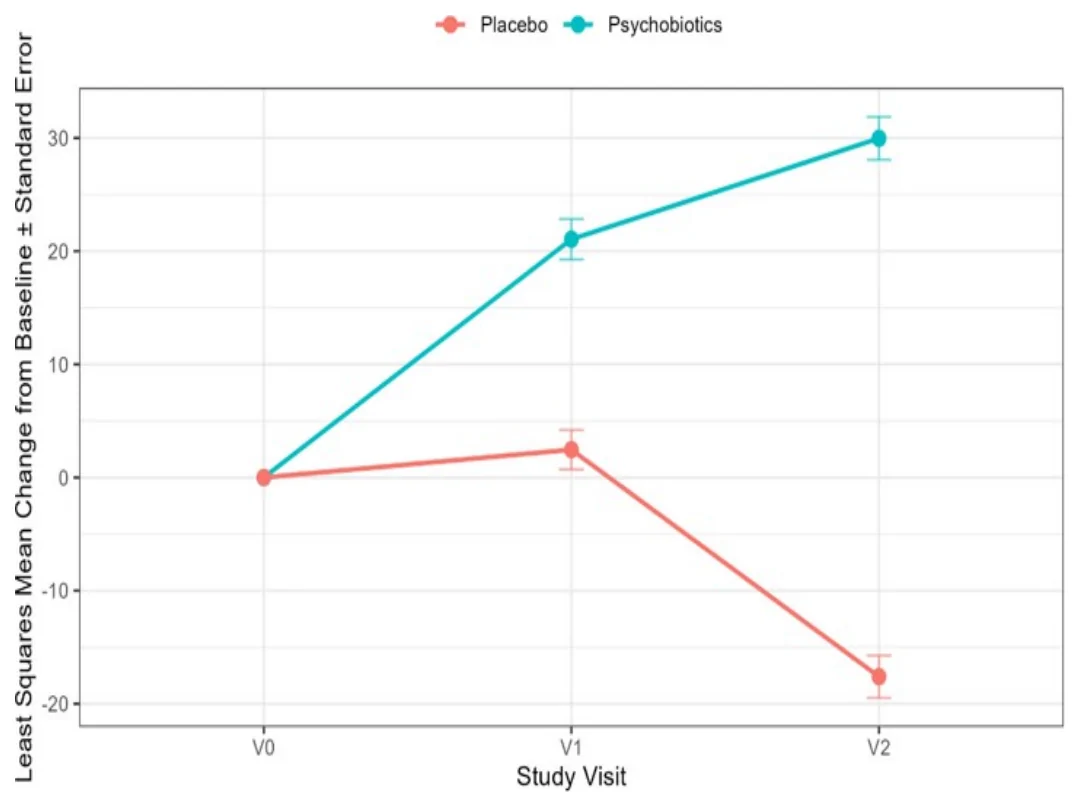
Least Squares Mean Change from Baseline in Role Physical [RP].

At V1, participants receiving psychobiotics demonstrated a higher adjusted LS mean change in RP than those receiving placebo by 18.60 points (95% CI: 13.96–23.24; *p* < 0.001). At V2, the corresponding difference was 47.58 points (95% CI: 42.60–52.55; *p* < 0.001) (Table 2 and Figure 5). Exploratory adjusted subgroup estimates are presented in Supplementary Table S3.2.

### 3.3. Bodily Pain [BP]

**Figure 6:**
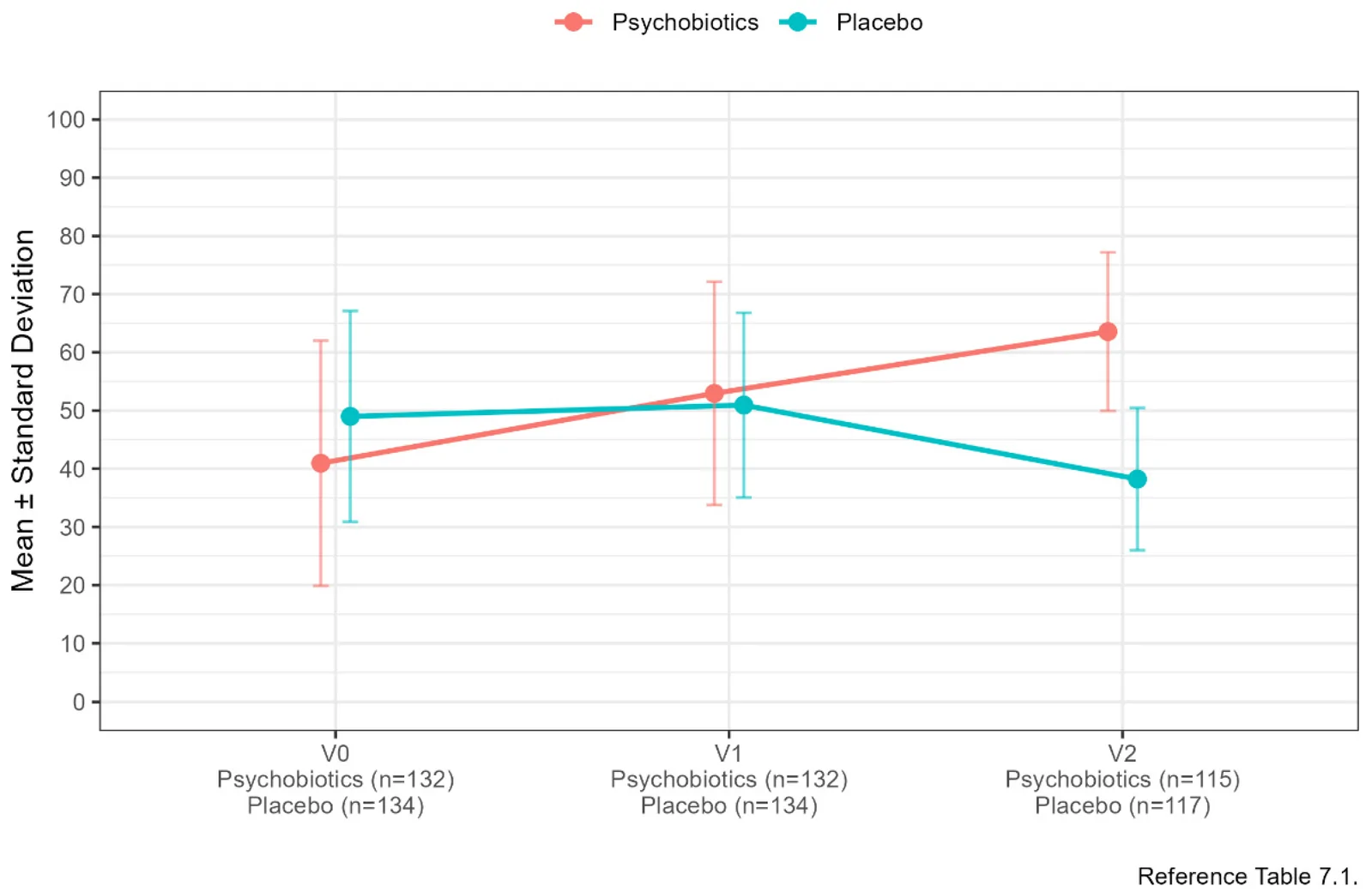
Bodily Pain [BP] by Visit and Group.

At baseline (V0), the mean ± SD BP score was 40.95 ± 21.07 in the psychobiotics group and 48.99 ± 18.11 in the placebo group. Within the psychobiotics group, the mean ± SD change from V0 to V1 was 12.01 ± 15.96 and that from V0 to V2 was 22.96 ± 15.86, whereas the corresponding changes in the placebo group were 1.94 ± 22.25 and −11.41 ± 19.95, respectively (Supplementary Table S1.3; Figure 6). Longitudinal analysis showed a significant group-by-visit interaction for BP (*p* < 0.001; Supplementary Table S2.3). Interactions between baseline characteristics and treatment group were not statistically significant.

**Figure 7:**
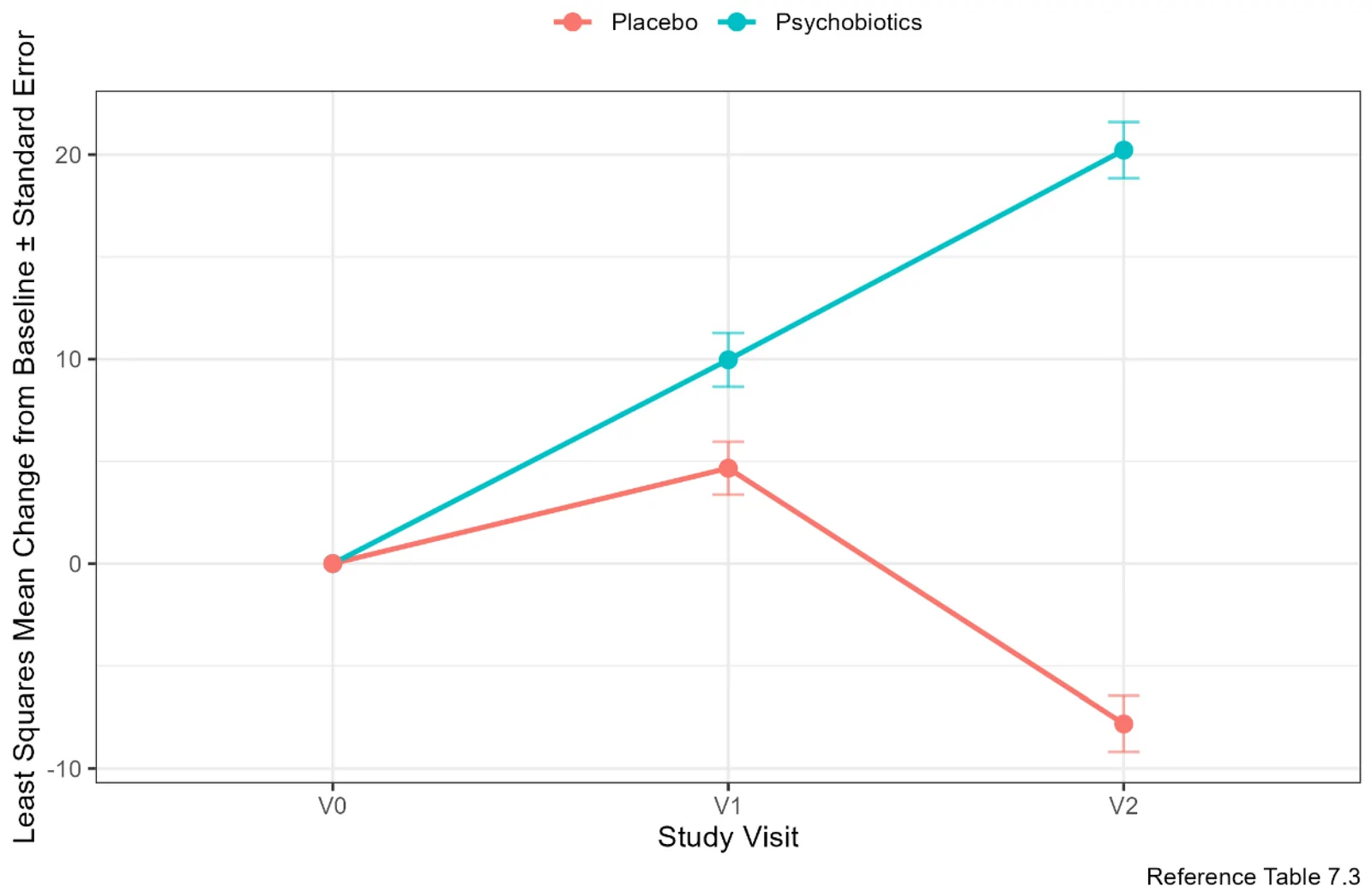
Least Squares Mean Change from Baseline in Bodily Pain [BP].

At V1, participants receiving psychobiotics demonstrated a higher adjusted LS mean change in BP than those receiving placebo by 5.30 points (95% CI: 1.89–8.71; *p* = 0.002). At V2, the corresponding difference was 28.05 points (95% CI: 24.44–31.65; *p* < 0.001) (Table 3 and Figure 7). Exploratory adjusted subgroup estimates are presented in Supplementary Table S3.3.

### 3.4. General Health [GH]

**Figure 8:**
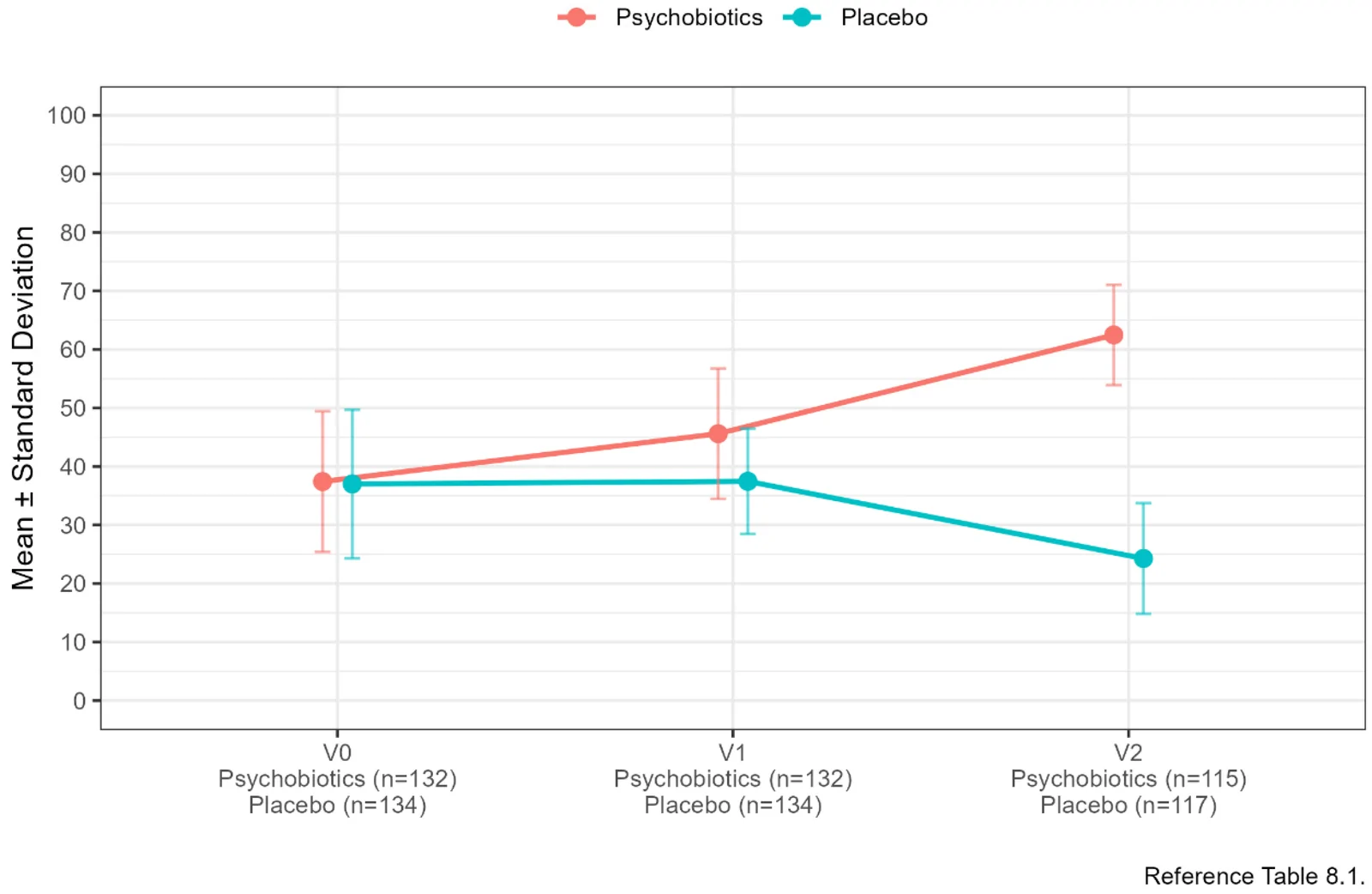
General Health [GH] by Visit and Group.

At baseline (V0), the mean ± SD GH score was 37.42 ± 12.02 in the psychobiotics group and 37.01 ± 12.72 in the placebo group. Within the psychobiotics group, the mean ± SD change from V0 to V1 was 8.18 ± 8.36 and that from V0 to V2 was 25.17 ± 9.62, whereas the corresponding changes in the placebo group were 0.45 ± 13.78 and −13.12 ± 13.98, respectively (Supplementary Table S1.4; Figure 8). Longitudinal analysis showed a significant group-by-visit interaction for GH (*p* < 0.001; Supplementary Table S2.4). Interactions between baseline characteristics and treatment group were not statistically significant.

**Figure 9:**
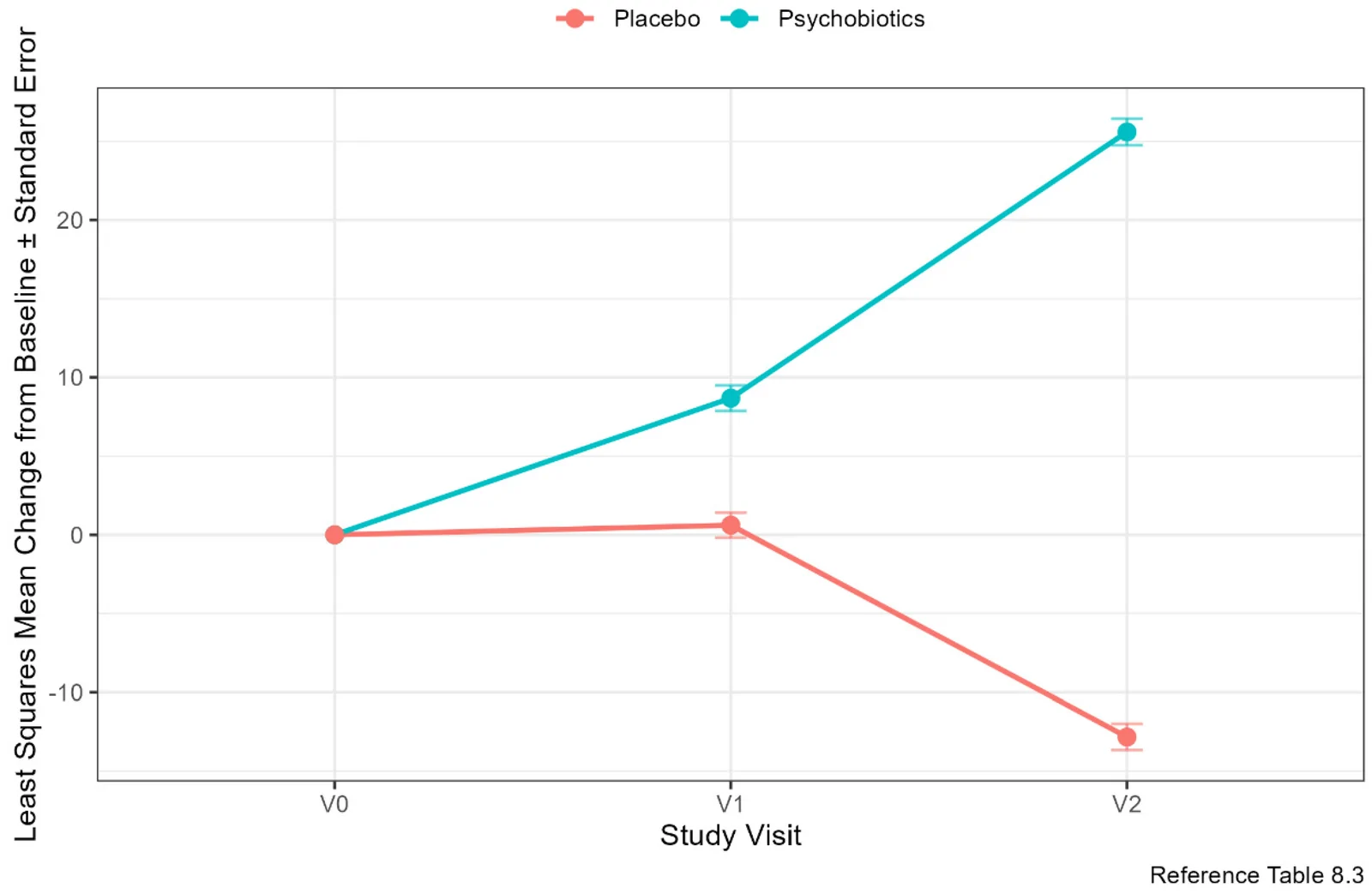
Least Squares Mean Change from Baseline in General Health [GH].

At V1, participants receiving psychobiotics demonstrated a higher adjusted LS mean change in GH than those receiving placebo by 8.07 points (95% CI: 6.01–10.13; *p* < 0.001). At V2, the corresponding difference was 38.42 points (95% CI: 36.26–40.58; *p* < 0.001) (Table 4 and Figure 9). Exploratory adjusted subgroup estimates are presented in Supplementary Table S3.4.

### 3.5. Vitality [VT]

**Figure 10:**
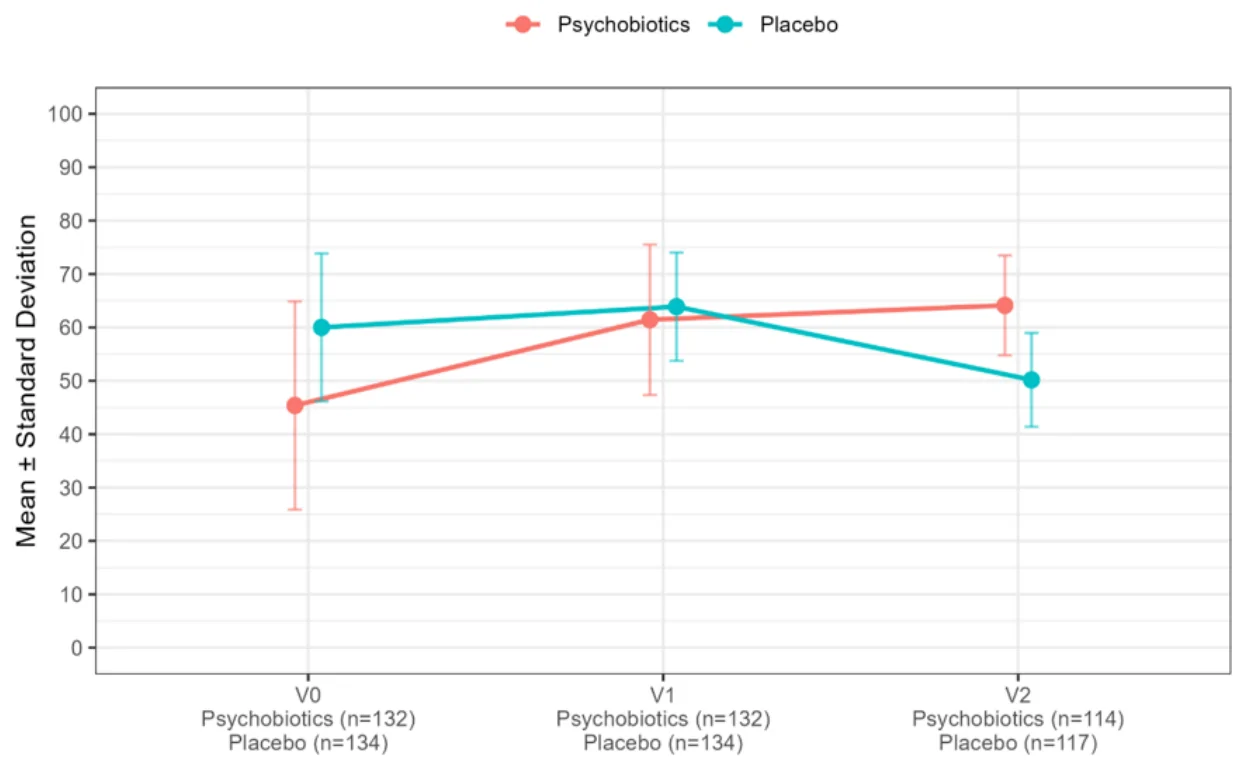
Vitality [VT] by Visit and Group.

At V0, the mean ± SD VT score was 45.38 ± 19.50 in the psychobiotics group and 60.00 ± 13.85 in the placebo group. Within the psychobiotics group, the mean ± SD change from V0 to V1 was 16.06 ± 17.66 and that from V0 to V2 was 18.68 ± 16.51, whereas the corresponding changes in the placebo group were 3.88 ± 17.64 and −9.57 ± 13.82, respectively (Supplementary Table S1.5; Figure 10). Longitudinal analysis showed a significant group-by-visit interaction for VT (*p* < 0.001; Supplementary Table S2.5). Interactions between baseline characteristics and treatment group were not statistically significant.

**Figure 11:**
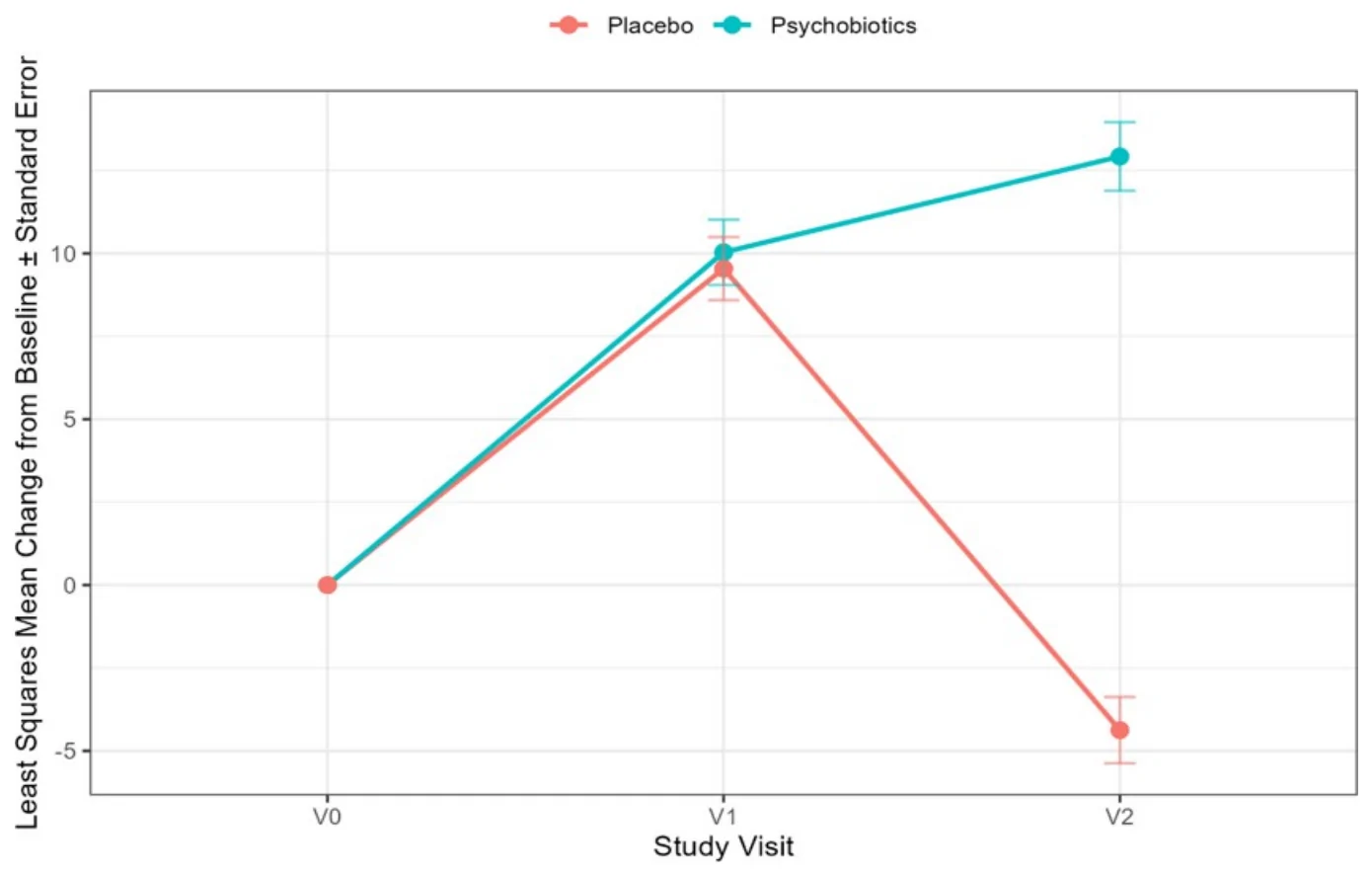
Least Squares Mean Change from Baseline in Vitality [VT].

At V1, no statistically significant difference between the psychobiotic and placebo groups was observed in adjusted LS mean change in VT. At V2, participants receiving psychobiotics demonstrated a higher adjusted LS mean change in VT than those receiving placebo by 17.30 points (95% CI: 14.53–20.07; *p* < 0.001) (Table 5 and Figure 11). Exploratory adjusted subgroup estimates are presented in Supplementary Table S3.5.

### 3.6. Social Functioning [SF]

**Figure 12:**
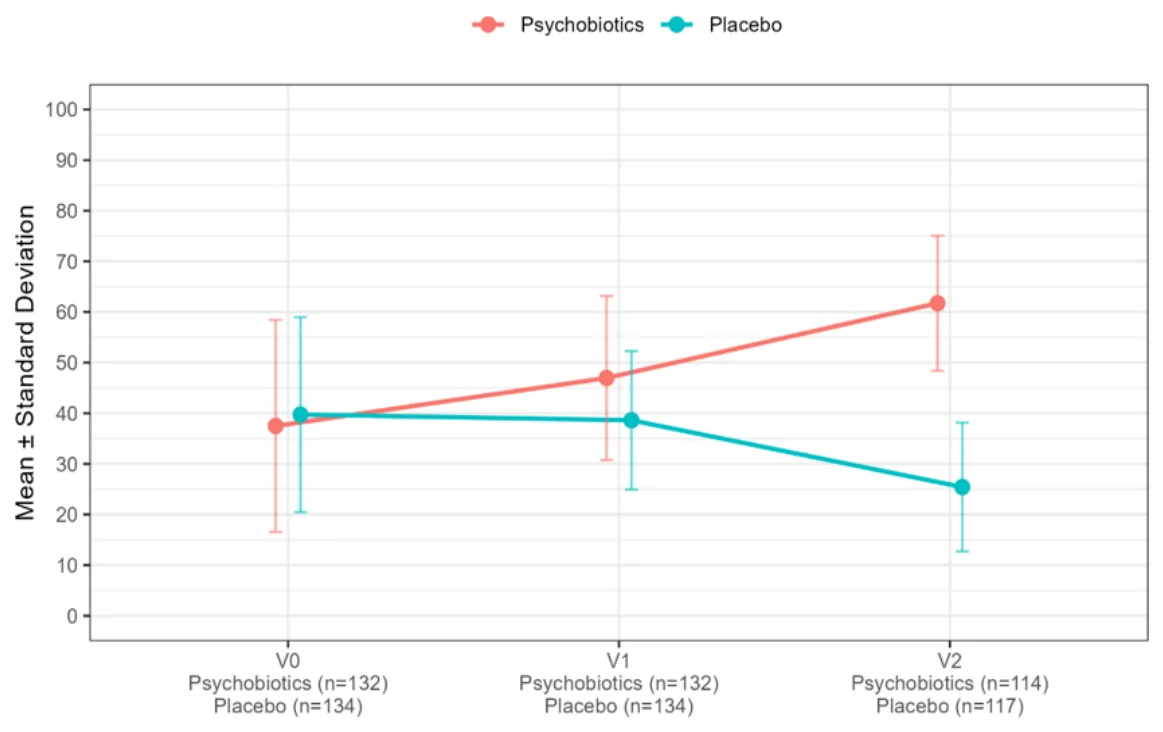
Social Functioning [SF] by Visit and Group.

At V0, the mean ± SD SF score was 37.50 ± 20.95 in the psychobiotics group and 39.74 ± 19.26 in the placebo group. Within the psychobiotics group, the mean ± SD change from V0 to V1 was 9.47 ± 13.65 and that from V0 to V2 was 25.44 ± 15.68, whereas the corresponding changes in the placebo group were −1.12 ± 20.07 and −14.74 ± 19.60, respectively (Supplementary Table S1.6; Figure 12). Longitudinal analysis showed a significant group-by-visit interaction for SF (*p* < 0.001; Supplementary Table S2.6). Interactions between baseline characteristics and treatment group were not statistically significant.

**Figure 13:**
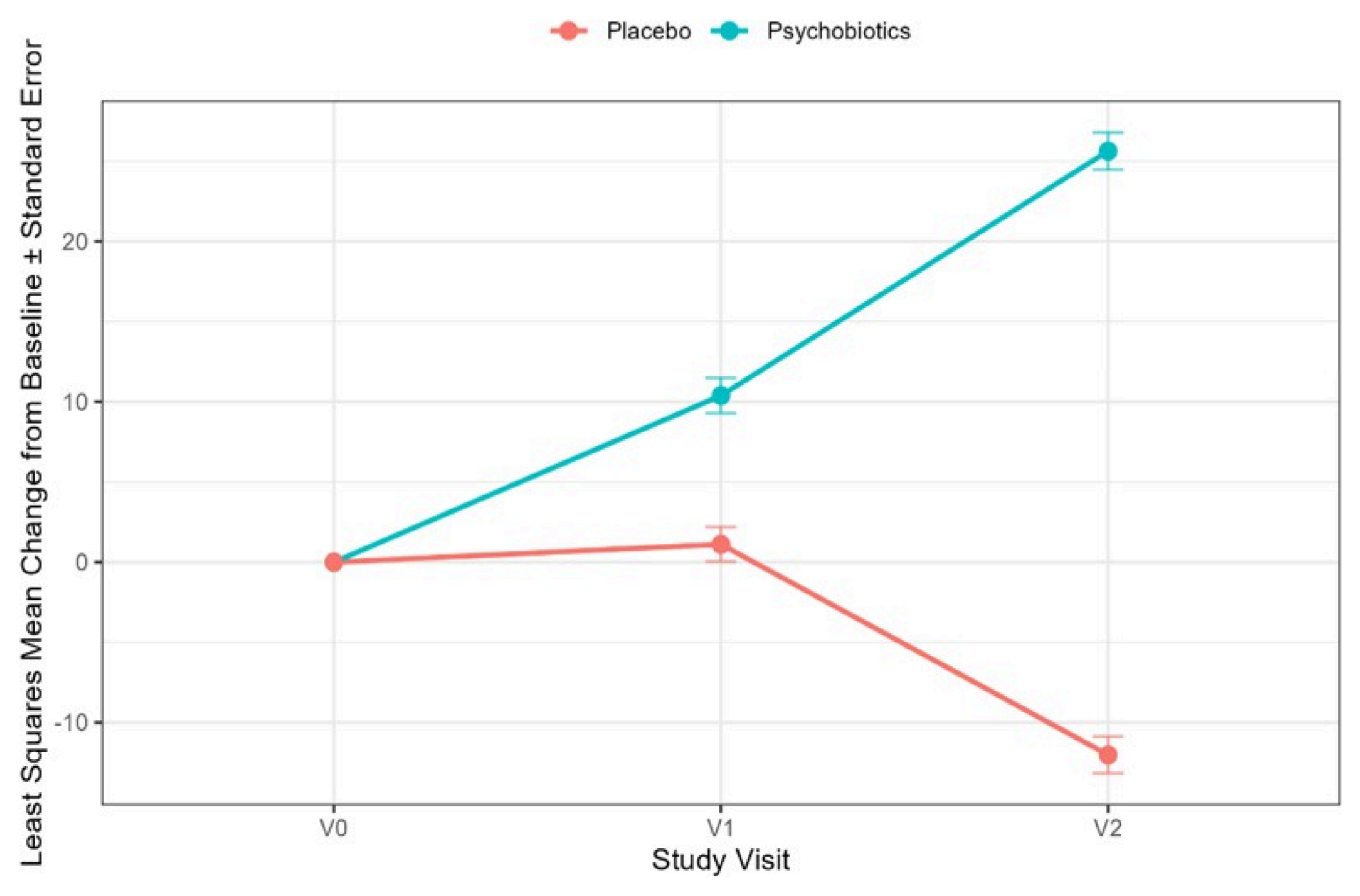
Least Squares Mean Change from Baseline in Social Functioning [SF].

At V1, participants receiving psychobiotics demonstrated a higher adjusted LS mean change in SF than those receiving placebo by 9.27 points (95% CI: 6.45–12.10; *p* < 0.001). At V2, the corresponding difference was 37.64 points (95% CI: 34.64–40.64; *p* < 0.001) (Table 6 and Figure 13). Exploratory adjusted subgroup estimates are presented in Supplementary Table S3.6.

### 3.7. Role Emotional [RE]

**Figure 14:**
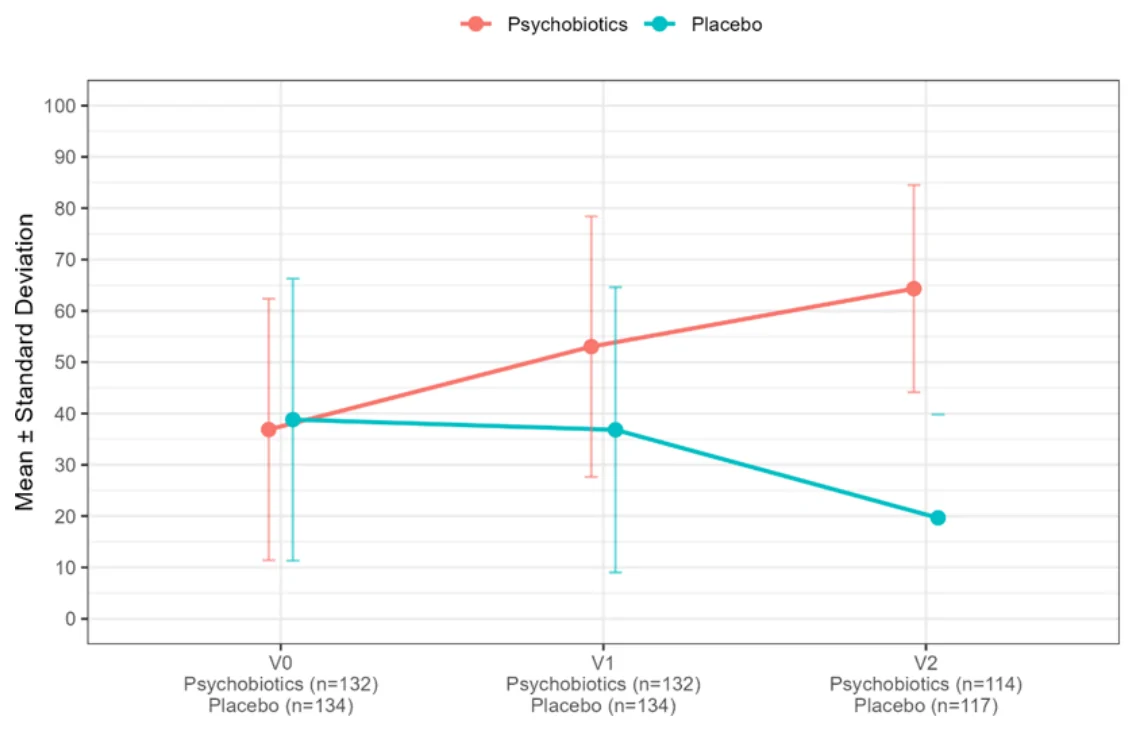
Role Emotional [RE] by Visit and Group.

At V0, the mean ± SD RE score was 36.87 ± 25.47 in the psychobiotics group and 38.81 ± 27.48 in the placebo group. Within the psychobiotics group, the mean ± SD change from V0 to V1 was 16.16 ± 20.38 and that from V0 to V2 was 27.78 ± 24.66, whereas the corresponding changes in the placebo group were −1.99 ± 35.58 and −19.09 ± 26.01, respectively (Supplementary Table S1.7; Figure 14). Longitudinal analysis showed a significant group-by-visit interaction for RE (*p* < 0.001; Supplementary Table S2.7). Interactions between baseline characteristics and treatment group were not statistically significant.

**Figure 15:**
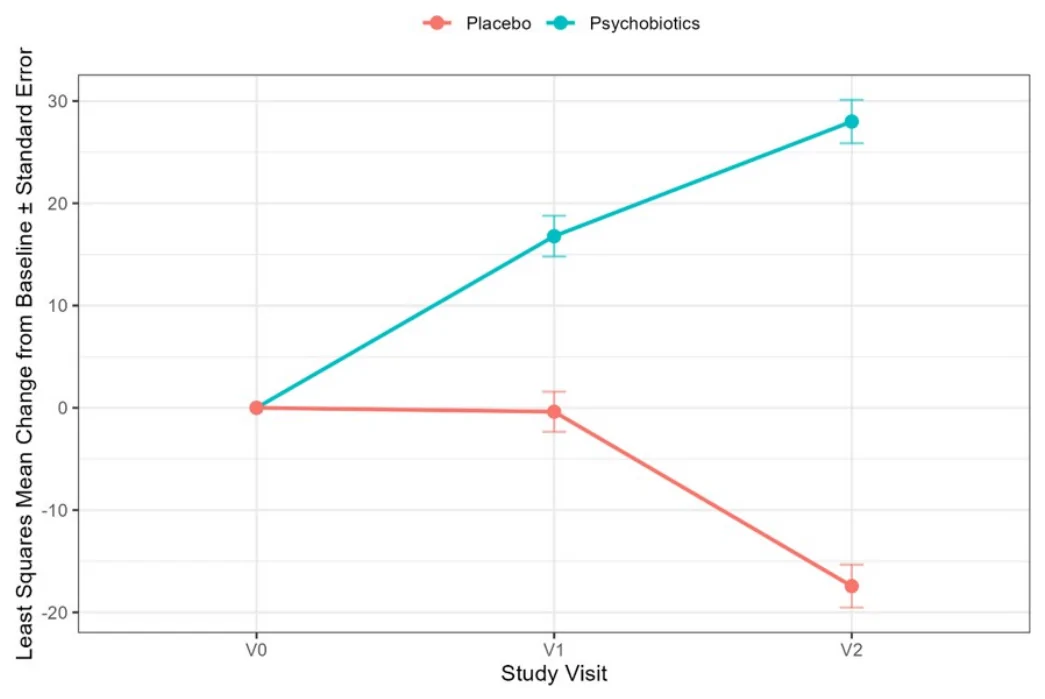
Least Squares Mean Change from Baseline in Role Emotional [RE].

At V1, participants receiving psychobiotics demonstrated a higher adjusted LS mean change in RE than those receiving placebo by 17.17 points (95% CI: 11.96–22.38; *p* < 0.001). At V2, the corresponding difference was 45.43 points (95% CI: 39.85–51.02; *p* < 0.001), indicating progressively greater separation between the two treatment groups over time (Table 7 and Figure 15). Exploratory adjusted subgroup estimates are presented in Supplementary Table S3.7.

### 3.8. Mental Health [MH]

**Figure 16:**
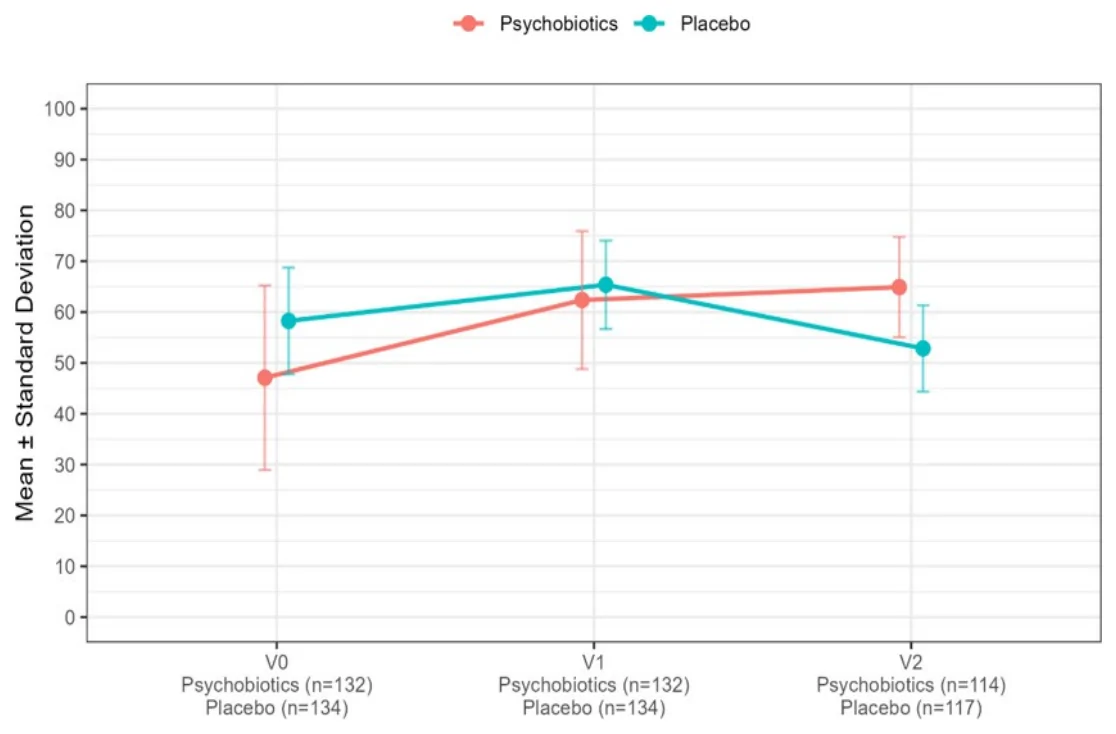
Mental Health [MH] by Visit and Group.

At V0, the mean ± SD MH score was 47.09 ± 18.12 in the psychobiotics group and 58.27 ± 10.48 in the placebo group. Within the psychobiotics group, the mean ± SD change from V0 to V1 was 15.27 ± 15.91 and that from V0 to V2 was 18.63 ± 14.04, whereas the corresponding changes in the placebo group were 7.10 ± 14.04 and −4.62 ± 12.17, respectively (Supplementary Table S1.8; Figure 16). Longitudinal analysis showed a significant group-by-visit interaction for MH (*p* < 0.001; Supplementary Table S2.8).Interactions between baseline characteristics and treatment group were not statistically significant.

**Figure 17:**
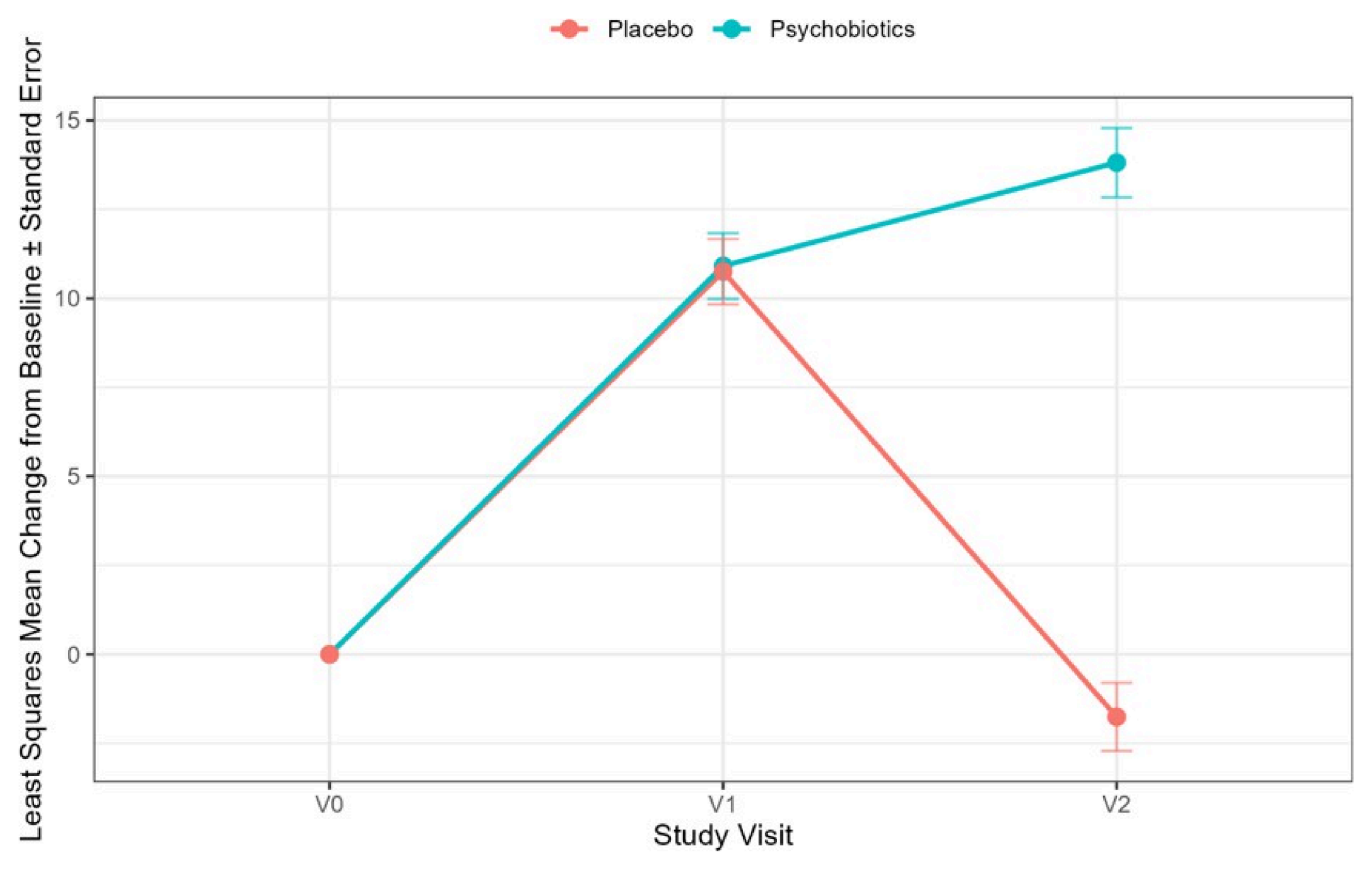
Least Squares Mean Change from Baseline in Mental Health [MH].

At V1, no statistically significant difference between the psychobiotic and placebo groups was observed in adjusted LS mean change in MH. At V2, participants receiving psychobiotics demonstrated a higher adjusted LS mean change in MH than those receiving placebo by 15.57 points (95% CI: 12.97–18.17; *p* < 0.001) (Table 8 and Figure 17). Exploratory adjusted subgroup estimates are presented in Supplementary Table S3.8.

### 3.9. Physical Component Summary [PCS]

**Figure 18:**
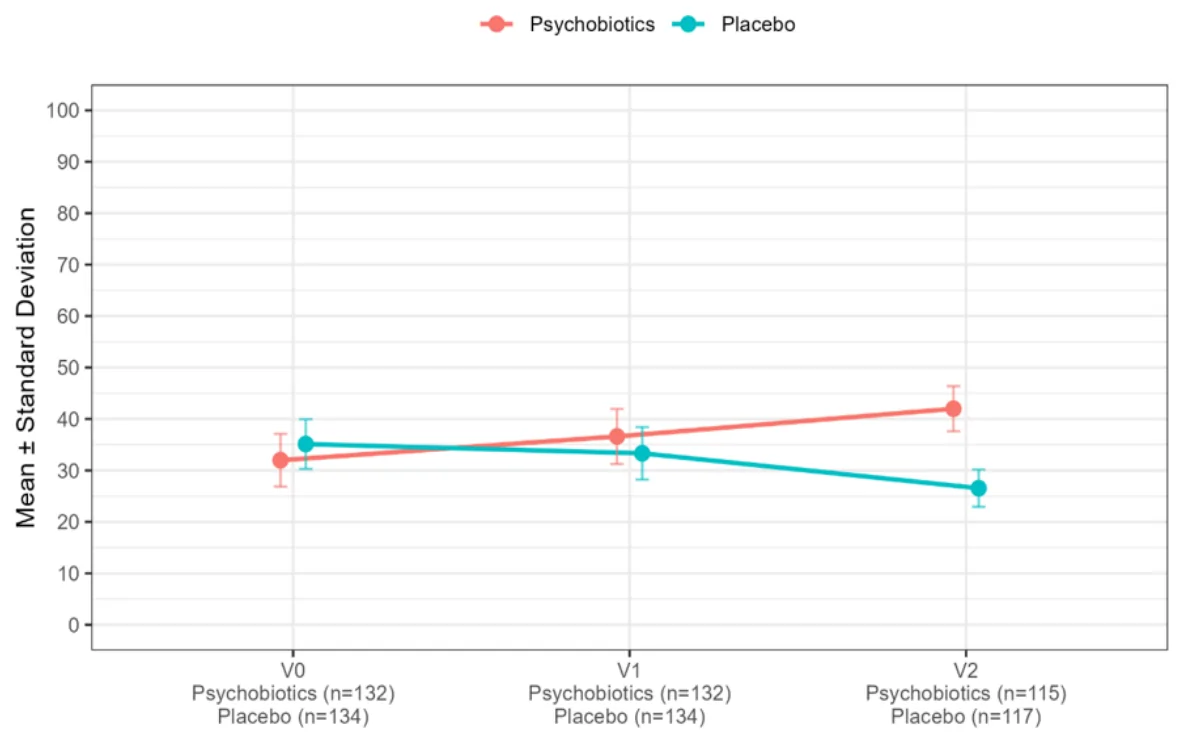
Physical Component Summary [PCS] by Visit and Group.

At V0, the mean ± SD PCS score was 31.98 ± 5.12 in the psychobiotics group and 35.12 ± 4.83 in the placebo group. Within the psychobiotics group, the mean ± SD change from V0 to V1 was 4.62 ± 3.86 and that from V0 to V2 was 10.00 ± 4.20, whereas the corresponding changes in the placebo group were −1.80 ± 6.03 and −8.76 ± 4.84, respectively (Supplementary Table S1.9; Figure 18). Longitudinal analysis showed a significant group-by-visit interaction for PCS (*p* < 0.001; Supplementary Table S2.9). Interactions between baseline characteristics and treatment group were not statistically significant.

**Figure 19:**
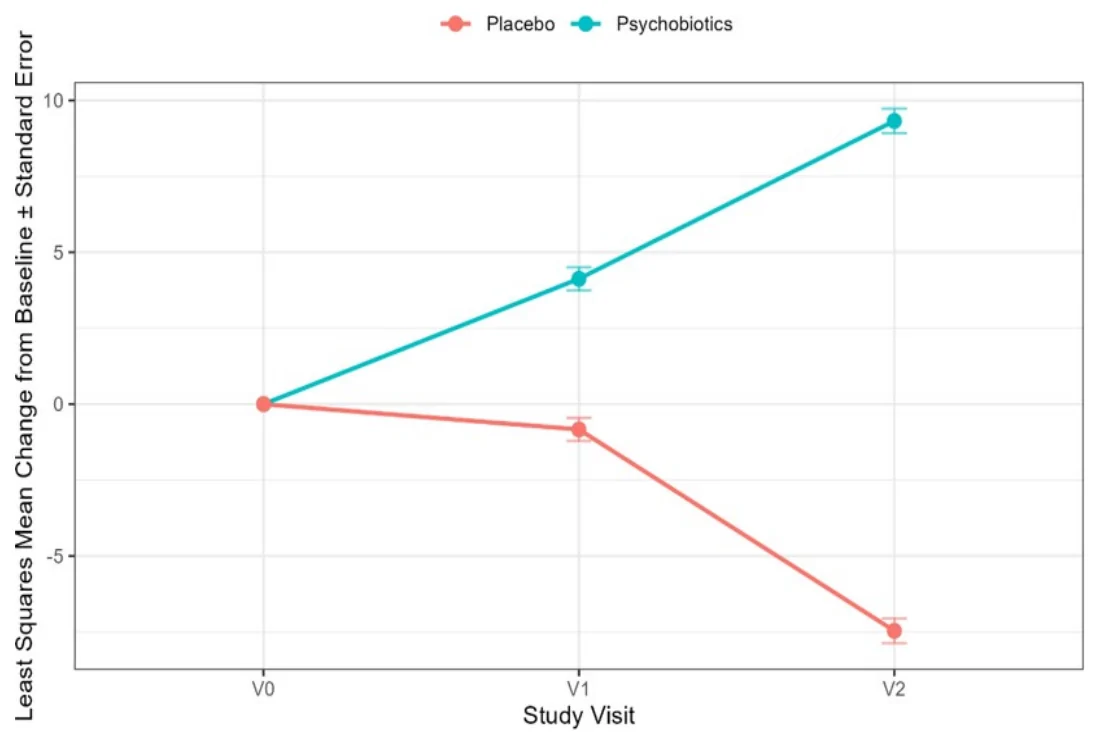
Least Squares Mean Change from Baseline in Physical Component Summary [PCS].

At V1, participants receiving psychobiotics demonstrated a higher adjusted LS mean change in PCS than those receiving placebo by 4.96 points (95% CI: 3.94–5.98; *p* < 0.001). At V2, the corresponding difference was 16.79 points (95% CI: 15.69–17.88; *p* < 0.001) (Table 9 and Figure 19). Exploratory adjusted subgroup estimates are presented in Supplementary Table S3.9.

### 3.10. Mental Component Summary [MCS]

**Figure 20:**
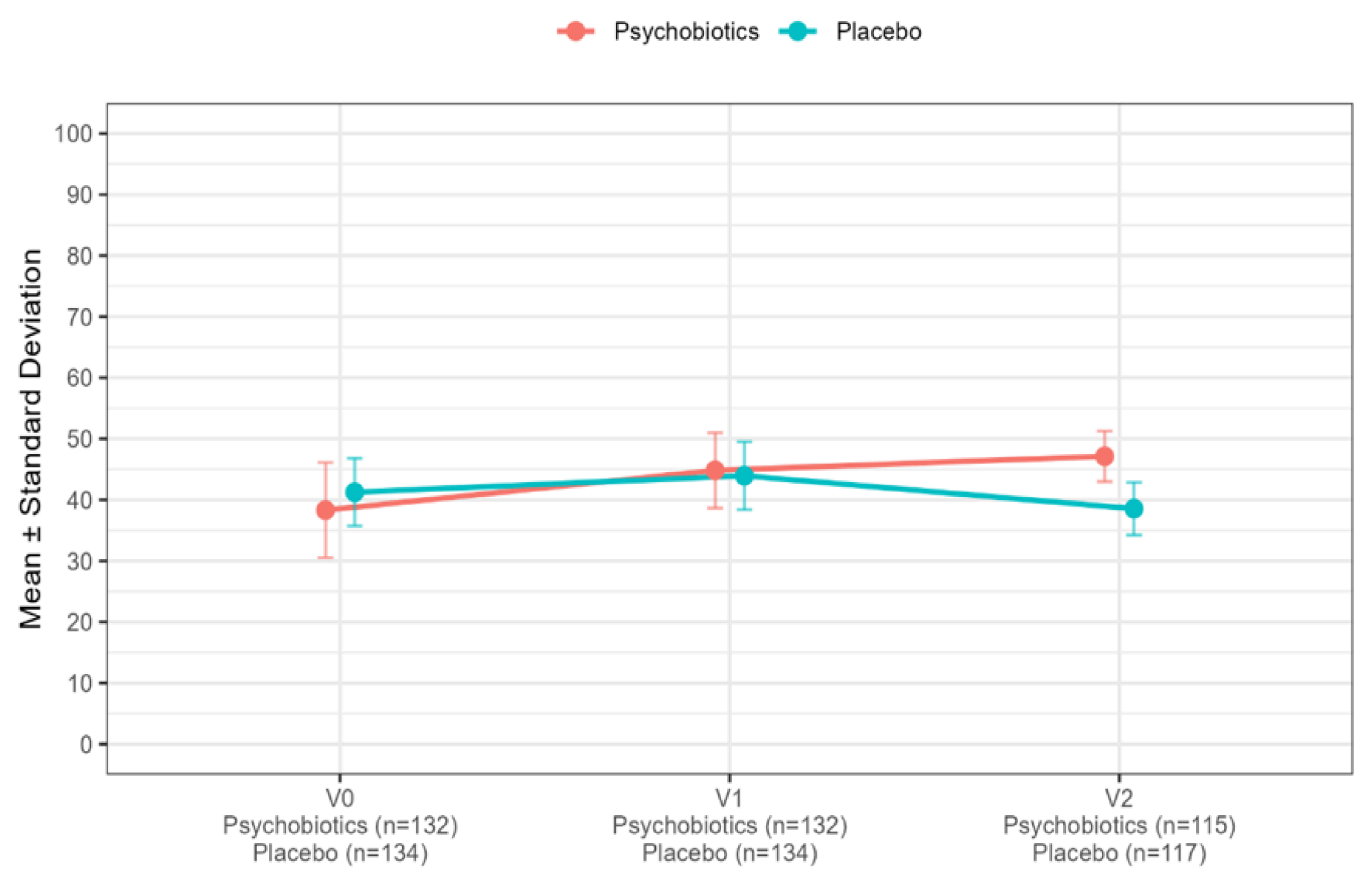
Mental Component Summary [MCS] by Visit and Group.

At V0, the mean ± SD MCS score was 38.32 ± 7.78 in the psychobiotics group and 41.26 ± 5.53 in the placebo group. Within the psychobiotics group, the mean ± SD change from V0 to V1 was 6.50 ± 5.86 and that from V0 to V2 was 9.17 ± 6.07, whereas the corresponding changes in the placebo group were 2.69 ± 6.85 and −2.48 ± 5.17, respectively (Supplementary Table S1.10; Figure 20). Longitudinal analysis showed a significant group-by-visit interaction for MCS (*p* < 0.001; Supplementary Table S2.10). Interactions between baseline characteristics and treatment group were not statistically significant.

**Figure 21:**
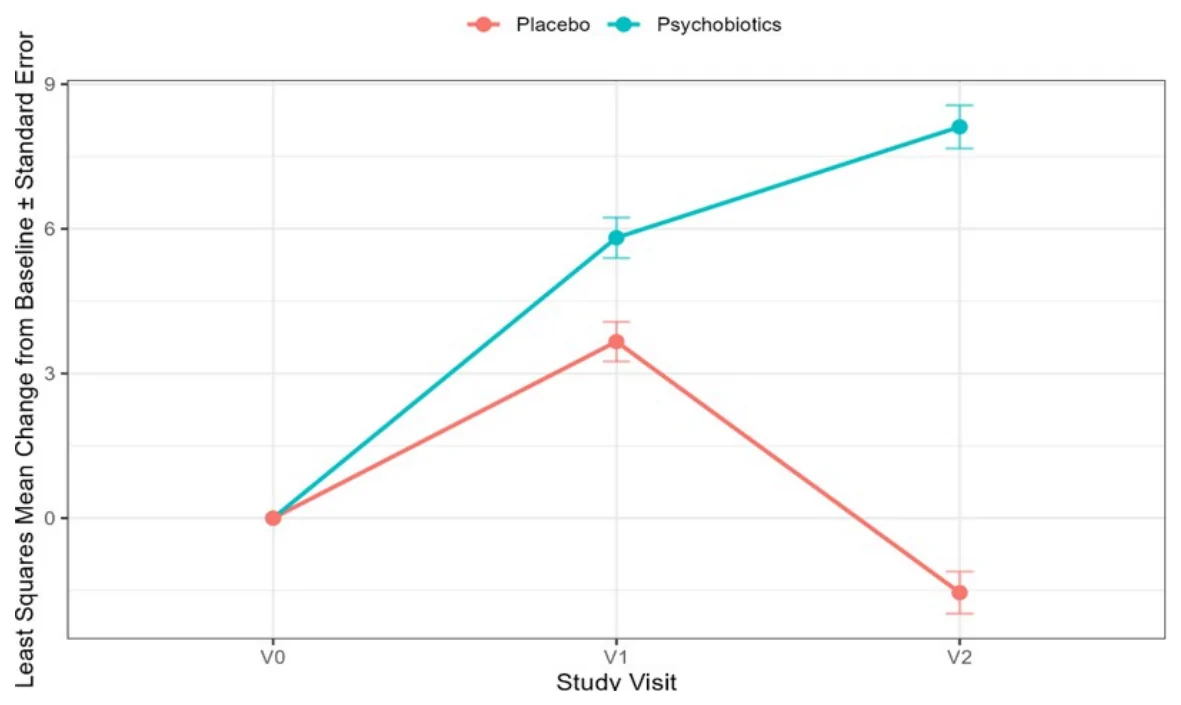
Least Squares Mean Change from Baseline in Mental Component Summary [MCS].

At V1, participants receiving psychobiotics demonstrated a higher adjusted LS mean change in MCS than those receiving placebo by 2.16 points (95% CI: 1.06–3.25; *p* < 0.001). At V2, the corresponding difference was 9.66 points (95% CI: 8.49–10.84; *p* < 0.001) (Table 10 and Figure 21). Exploratory adjusted subgroup estimates are presented in Supplementary Table S3.10.

## 4. Discussion

Cancer survivors may experience persistent physical and psychosocial burdens related to both the disease and its treatment, with HR-QoL frequently remaining impaired even after therapy [24,38,45,46,47,48,49,50]. These impairments are particularly relevant in gastrointestinal malignancies, where surgery and multimodal oncological treatment may contribute to persistent gastrointestinal symptoms through altered anatomy and digestive function, impaired mucosal integrity, and treatment-related dysbiosis [51,52,53,54,55,56,57].

Multimodal cancer treatment is associated with a broad range of toxicities that may adversely affect physical, emotional, cognitive, and social functioning. Chemotherapy-related mucositis, peripheral neuropathy, fatigue, sleep and nutritional disturbances, together with gastrointestinal and functional complications of surgery, may contribute substantially to impaired HR-QoL during and after treatment [7,11,12,20,58,59,60,61,62].

Experimental and clinical evidence further supports the association between chemotherapy and disturbances relevant to HR-QoL. Chemotherapy has been associated with anxiety-like behavior, fatigue, cognitive impairment, inflammatory signaling, dysbiosis-related pain, and, in clinical studies, deterioration in cognitive performance, depression, anxiety, pain perception, and emotional HR-QoL [24,35,36,63,64,65,66]. These findings provide a biologically plausible context for HR-QoL deterioration during systemic cancer treatment.

HR-QoL is increasingly recognized as an important patient-reported outcome in oncology, complementing traditional endpoints such as tumor response and survival and informing the balance between treatment benefit and burden [2,3,4,67,68,69,70]. Its incorporation into cancer clinical trials is also supported by regulatory guidance [68]. In this context, the present analysis focused specifically on longitudinal changes in patient-reported HR-QoL.

In the present secondary analysis of prospectively collected data from the ProDeCa randomized trial [35,36], psychobiotic treatment was associated with more favorable longitudinal HR-QoL outcomes than placebo among patients undergoing adjuvant chemotherapy after gastrointestinal cancer surgery. HR-QoL was assessed across the eight SF-36 domains and the Physical and Mental Component Summary scores, providing a multidimensional evaluation of physical and mental health throughout follow-up [6,12,37,38,39,40,41,42,43,44].

The principal finding of the present study should not be interpreted simply as higher HR-QoL scores in the psychobiotic group. Rather, the most important observation was the progressive divergence in HR-QoL trajectories between the two randomized groups over time. Mixed models for repeated measures evaluate longitudinal changes rather than isolated cross-sectional differences, and the significant treatment-by-visit interactions observed across most SF-36 domains indicate that the magnitude of the treatment effect increased progressively throughout follow-up. Thus, psychobiotic treatment was associated not merely with more favorable HR-QoL at individual time points, but with progressively divergent longitudinal trajectories of patient-reported physical and mental health compared with placebo. An equally important observation was the progressive deterioration observed among placebo-treated participants. Although this pattern may initially appear unexpected, it is entirely consistent with the clinical course frequently observed following major gastrointestinal cancer surgery. During postoperative recovery, patients remain exposed to cumulative chemotherapy-related toxicities, nutritional disturbances, persistent gastrointestinal symptoms, fatigue, peripheral neuropathy, sleep disturbances, chronic pain, and continuing psychological stress associated with fear of recurrence and uncertainty regarding treatment outcomes. The combined burden of these factors provides a biologically and clinically plausible explanation for the gradual deterioration in HR-QoL observed in placebo-treated participants. Conversely, the progressive improvement observed in the psychobiotic group is consistent with the hypothesis that modulation of the gut–brain axis may be associated with more favorable HR-QoL trajectories during postoperative recovery and adjuvant therapy; however, the present analysis cannot establish the underlying mechanism.

Baseline scores were low across all eight SF-36 domains in both groups, indicating substantial HR-QoL impairment before initiation of the study intervention. As baseline assessment occurred after radical surgery and during adjuvant chemotherapy, this impairment likely reflects the combined burden of cancer and multimodal treatment rather than chemotherapy alone, consistent with previous reports in gastrointestinal and other oncological populations [12,13,37,48,49,71].

In gastrointestinal oncology, HR-QoL impairment may reflect the cumulative burden of cancer diagnosis, major surgery, and adjuvant chemotherapy rather than a single event [70,72,73,74,75]. This sequential “multiple-hit” model provides a useful clinical framework for understanding persistent or progressive HR-QoL impairment and highlights the potential importance of supportive interventions throughout the cancer trajectory.

The intestinal microbiota may influence emotional, cognitive, immune, and neuroendocrine function through the gut–brain axis, providing a rationale for microbiota-targeted interventions [55,60,76]. Psychobiotics have shown potentially beneficial effects on depression, anxiety, perceived stress, and pain in gastrointestinal oncology patients, including in the ProDeCa cohort [35,36,62,77,78,79,80,81]. These findings provide relevant context for the present results, as psychological distress, pain, sleep quality, and physical functioning are closely related to HR-QoL.

Regarding the effects of psychobiotic treatment on HR-QoL, longitudinal mixed-model analyses demonstrated significant group-by-visit interactions across the eight SF-36 domains (Supplementary Tables S2.1–S2.10; all *p* < 0.001). After adjustment for baseline score and clinically relevant covariates, psychobiotic-treated participants generally demonstrated improvements throughout follow-up, whereas placebo-treated participants generally exhibited deterioration across individual domains and the Physical and Mental Component Summary scores. The increasing between-group differences over follow-up, particularly at Visit 2, therefore reflect divergent longitudinal HR-QoL trajectories rather than an isolated end-of-study difference.

The progressive deterioration observed among placebo-treated participants should be interpreted cautiously. Although the longitudinal models adjusted for baseline HR-QoL and covariates and handled missing observations under the Missing At Random assumption, detailed longitudinal data on disease progression, hospitalization, and other clinical events were not systematically available for the present analysis. Therefore, the factors underlying the observed deterioration in the placebo group cannot be determined from the present data.

Given the clinical heterogeneity of the study population, exploratory subgroup analyses were conducted according to gender, tumor location, depression status, and persistent pain [35,36,81,82,83,84,85,86,87,88]. However, interactions between these baseline characteristics and treatment group were not statistically significant. Accordingly, the subgroup estimates should be considered descriptive and hypothesis-generating and should not be interpreted as evidence of differential treatment effects.

Depression may nevertheless be relevant to the interpretation of patient-reported outcomes because mood can influence the cognitive and emotional processing of bodily sensations and symptom reporting. This consideration is consistent with our previous findings regarding pain perception in the ProDeCa cohort [36]. The Gate-control theory of pain provides a conceptual framework whereby emotional and cognitive processes may modulate the transmission and conscious interpretation of somatic stimuli [81,89]. Accordingly, depression-related differences in subjective symptom perception may provide useful context when interpreting exploratory HR-QoL estimates but should not be considered evidence of differential treatment effects in the absence of significant treatment-by-depression interactions.

HR-QoL may vary across populations because it is influenced by sociocultural, socioeconomic, healthcare, and lifestyle factors. In the present cohort, some of these sources of variability were relatively limited because participants shared broadly similar cultural and socioeconomic backgrounds, received treatment within the same publicly funded healthcare system and institution, and generally had strong family support. Nevertheless, financial difficulties, stoma formation, unemployment, comorbidity burden, and lifestyle factors have been associated with poorer HR-QoL in gastrointestinal cancer populations [9,78,88,90,91]. These observations emphasize the multifactorial nature of HR-QoL and should be considered when extrapolating the present findings to populations with different sociocultural and healthcare characteristics.

Several limitations should be considered when interpreting the findings of the present study. First, this was a post hoc analysis of the ProDeCa randomized trial, and the trial was not specifically powered to detect differences across individual SF-36 domains or within exploratory subgroup analyses. Findings regarding gender, depression, pain status, and tumor location should therefore be considered exploratory and hypothesis-generating. Moreover, multiple HR-QoL domains, summary scores, follow-up comparisons, and subgroup analyses were evaluated without formal adjustment for multiplicity, increasing the possibility of type I error; individual *p*-values should therefore be interpreted alongside the magnitude and precision of the estimated effects. Estimates from highly stratified subgroups with small sample sizes should be interpreted cautiously and were not considered evidence of differential treatment effects. Second, the single-center design and relatively homogeneous study population may limit the generalizability of the findings to populations with different cultural, socioeconomic, healthcare, or oncological treatment characteristics. Third, as a patient-reported outcome, HR-QoL may be influenced by individual psychosocial factors that cannot be fully quantified or controlled, despite the use of a validated assessment instrument. Fourth, HR-QoL was assessed using the generic SF-36 rather than a cancer-specific instrument such as the EORTC QLQ-C30, which may have limited the assessment of cancer- and treatment-specific symptoms and direct comparison with studies using cancer-specific measures. Nevertheless, the SF-36 was aligned with the study objective of evaluating broader physical and mental dimensions of HR-QoL. Fifth, follow-up was limited to three months, precluding assessment of the longer-term persistence of the observed HR-QoL changes. Finally, the study did not include biological measurements of gut microbiota composition, inflammatory biomarkers, microbial metabolites, neuroendocrine mediators, or other indicators of gut–brain axis activity; therefore, the mechanisms underlying the observed HR-QoL changes cannot be determined. Although the longitudinal models adjusted for the corresponding baseline SF-36 score and included a baseline-by-visit interaction, baseline imbalances observed in several domains should also be considered when interpreting the adjusted between-group estimates. In addition, although several relevant clinical characteristics were comparable between treatment groups and tumor location was included in the adjusted models, other potentially relevant factors, including specific surgical procedure, stoma status, chemotherapy cycle, and time since surgery, were not incorporated into the present analysis. Detailed longitudinal information on chemotherapy toxicity, disease progression, hospitalization, treatment modifications, and other clinical events was also not incorporated; therefore, the potential contribution of these factors to the observed HR-QoL trajectories, particularly the deterioration in the placebo group, cannot be determined.

Despite these limitations, the study has several strengths, including its placebo-controlled design, prospectively collected HR-QoL data from the ProDeCa randomized trial, and longitudinal assessment using a validated instrument. The use of mixed models for repeated measures allowed evaluation of HR-QoL trajectories while accounting for within-subject correlations and incomplete follow-up data.

## 5. Conclusions

In gastrointestinal oncology patients undergoing adjuvant chemotherapy after radical tumor resection, psychobiotic supplementation was associated with more favorable longitudinal HR-QoL outcomes across multiple physical and mental dimensions assessed by the SF-36. Psychobiotic-treated participants generally demonstrated improvement over follow-up, whereas placebo-treated participants generally exhibited deterioration, resulting in increasingly divergent HR-QoL trajectories between groups. These findings support further investigation of psychobiotic supplementation as a non-invasive adjunctive strategy for HR-QoL support in gastrointestinal oncology. Exploratory subgroup estimates should be interpreted cautiously and do not provide evidence of differential treatment effects in the absence of significant formal interaction tests. More broadly, comprehensive cancer care should consider not only disease control and survival but also patients’ physical functioning, emotional well-being, and social participation throughout treatment.

## Figures and Tables

**Table 1:** HR-QoL: Physical Function [PF].

Least Squares Mean Estimates for Change from Baseline by Visit and Group					
			95% Confidence Interval		
Visit	Group	LS Mean	Lower Bound	Upper Bound	*p*-Value
V1	Psychobiotics	9.72	7.52	11.93	
	Placebo	−3.86	−6.03	−1.68	
	Difference	13.58	10.33	16.83	<0.001
V2	Psychobiotics	19.60	17.27	21.93	
	Placebo	−23.05	−25.36	−20.73	
	Difference	42.64	39.18	46.11	<0.001

LS: Least Squares. Difference: LS mean change from baseline in the Psychobiotics group minus LS mean change from baseline in the Placebo group. Refer to Supplementary Table S2.1. Estimates are adjusted for visit, group, baseline, gender, depression status, pain status, and cancer location, including visit-by-group and visit-by-baseline interactions.

**Table 2:** HR-QoL: Role Physical [RP].

Least Squares Mean Estimates for Change from Baseline by Visit and Group					
			95% Confidence Interval		
Visit	Group	LS Mean	Lower Bound	Upper Bound	*p*-Value
V1	Psychobiotics	21.05	17.54	24.57	
	Placebo	2.46	−0.98	5.89	
	Difference	18.60	13.96	23.24	<0.001
V2	Psychobiotics	29.97	26.25	33.70	
	Placebo	−17.60	−21.27	−13.93	
	Difference	47.58	42.60	52.55	<0.001

LS: Least Squares. Difference: LS mean change from baseline in the Psychobiotics group minus LS mean change from baseline in the Placebo group. Refer to Supplementary Table S2.2. Estimates are adjusted for visit, group, baseline, gender, depression status, pain status, and cancer location, including visit-by-group and visit-by-baseline interactions.

**Table 3:** HR-QoL: Bodily Pain [BP].

Least Squares Mean Estimates for Change from Baseline by Visit and Group					
			95% Confidence Interval		
Visit	Group	LS Mean	Lower Bound	Upper Bound	*p*-Value
V1	Psychobiotics	9.97	7.38	12.55	
	Placebo	4.67	2.12	7.21	
	Difference	5.30	1.89	8.71	0.002
V2	Psychobiotics	20.22	17.52	22.92	
	Placebo	−7.83	−10.51	−5.14	
	Difference	28.05	24.44	31.65	<0.001

LS: Least Squares. Difference: LS mean change from baseline in the Psychobiotics group minus LS mean change from baseline in the Placebo group. Refer to Supplementary Table S2.3. Estimates are adjusted for visit, group, baseline, gender, depression status, pain status, and cancer location, including visit-by-group and visit-by-baseline interactions.

**Table 4:** HR-QoL: General Health [GH].

Least Squares Mean Estimates for Change from Baseline by Visit and Group					
Visit	Group	LS Mean	95% Lower CI	95% Upper CI	*p*-Value
	Psychobiotics	8.68	7.09	10.27	
	Placebo	0.61	−0.95	2.17	
V1	Difference	8.07	6.01	10.13	<0.001
	Psychobiotics	25.60	23.94	27.25	
	Placebo	−12.83	−14.46	−11.19	
V2	Difference	38.42	36.26	40.58	<0.001

LS: Least Squares. Difference: LS mean change from baseline in the Psychobiotics group minus LS mean change from baseline in the Placebo group. Refer to Supplementary Table S2.4. Estimates are adjusted for visit, group, baseline, gender, depression status, pain status, and cancer location, including visit-by-group and visit-by-baseline interactions.

**Table 5:** HR-QoL: Vitality [VT].

Least Squares Mean Estimates for Change from Baseline by Visit and Group					
			95% Confidence Interval		
Visit	Group	LS Mean	Lower Bound	Upper Bound	*p*-Value
V1	Psychobiotics	10.03	8.10	11.97	
	Placebo	9.54	7.67	11.41	
	Difference	0.49	−2.13	3.12	0.7125
V2	Psychobiotics	12.92	10.89	14.95	
	Placebo	−4.37	−6.34	−2.41	
	Difference	17.30	14.53	20.07	<0.001

LS: Least Squares. Difference: LS mean change from baseline in the Psychobiotics group minus LS mean change from baseline in the Placebo group. Refer to Supplementary Table S2.5. Estimates are adjusted for visit, group, baseline, gender, depression status, pain status, and cancer location, including visit-by-group and visit-by-baseline interactions.

**Table 6:** HR-QoL: Social Functioning [SF].

Least Squares Mean Estimates for Change from Baseline by Visit and Group					
			95% Confidence Interval		
Visit	Group	LS Mean	Lower Bound	Upper Bound	*p*-Value
V1	Psychobiotics	10.38	8.22	12.55	
	Placebo	1.11	−1.01	3.23	
	Difference	9.27	6.45	12.10	<0.001
V2	Psychobiotics	25.62	23.35	27.90	
	Placebo	−12.02	−14.26	−9.78	
	Difference	37.64	34.64	40.64	<0.001

LS: Least Squares. Difference: LS mean change from baseline in the Psychobiotics group minus LS mean change from baseline in the Placebo group. Refer to Supplementary Table S6.2. Estimates are adjusted for visit, group, baseline, gender, depression status, pain status, and cancer location, including visit-by-group and visit-by-baseline interactions.

**Table 7:** HR-QoL: Role Emotional [RE].

Least Squares Mean Estimates for Change from Baseline by Visit and Group					
			95% Confidence Interval		
Visit	Group	LS Mean	Lower Bound	Upper Bound	*p*-Value
V1	Psychobiotics	16.79	12.87	20.70	
	Placebo	−0.38	−4.23	3.47	
	Difference	17.17	11.96	22.38	<0.001
V2	Psychobiotics	28.00	23.83	32.17	
	Placebo	−17.44	−21.56	−13.32	
	Difference	45.43	39.85	51.02	<0.001

LS: Least Squares. Difference: LS mean change from baseline in the Psychobiotics group minus LS mean change from baseline in the Placebo group. Refer to Supplementary Table S2.7. Estimates are adjusted for visit, group, baseline, gender, depression status, pain status, and cancer location, including visit-by-group and visit-by-baseline interactions.

**Table 8:** HR-QoL: Mental Health [MH].

Least Squares Mean Estimates for Change from Baseline by Visit and Group					
			95% Confidence Interval		
Visit	Group	LS Mean	Lower Bound	Upper Bound	*p*-Value
V1	Psychobiotics	10.91	9.10	12.72	
	Placebo	10.75	8.95	12.55	
	Difference	0.16	−2.31	2.63	0.8983
V2	Psychobiotics	13.81	11.90	15.73	
	Placebo	−1.76	−3.64	0.12	
	Difference	15.57	12.97	18.17	<0.001

LS: Least Squares. Difference: LS mean change from baseline in the Psychobiotics group minus LS mean change from baseline in the Placebo group. Refer to Supplementary Table S2.8. Estimates are adjusted for visit, group, baseline, gender, depression status, pain status, and cancer location, including visit-by-group and visit-by-baseline interactions.

**Table 9:** HR-QoL: Physical Component Summary [PCS].

Least Squares Mean Estimates for Change from Baseline by Visit and Group					
Visit	Group	LS Mean	95% Lower CI	95% Upper CI	*p*-Value
	Psychobiotics	4.13	3.38	4.88	
	Placebo	−0.83	−1.58	−0.09	
V1	Difference	4.96	3.94	5.98	<0.001
	Psychobiotics	9.32	8.53	10.12	
	Placebo	−7.46	−8.26	−6.66	
V2	Difference	16.79	15.69	17.88	<0.001

LS: Least Squares. Difference: LS mean change from baseline in the Psychobiotics group minus LS mean change from baseline in the Placebo group. Refer to Supplementary Table S2.9. Estimates are adjusted for visit, group, baseline, gender, depression status, pain status, and cancer location, including visit-by-group and visit-by-baseline interactions.

**Table 10:** HR-QoL: Mental Component Summary [MCS].

Least Squares Mean Estimates for Change from Baseline by Visit and Group					
			95% Confidence Interval		
Visit	Group	LS Mean	Lower Bound	Upper Bound	*p*-Value
	Psychobiotics	5.82	4.99	6.64	
	Placebo	3.66	2.85	4.47	
V1	Difference	2.16	1.06	3.25	<0.001
	Psychobiotics	8.12	7.24	9.00	
	Placebo	−1.55	−2.40	−0.69	
V2	Difference	9.66	8.49	10.84	<0.001

LS: Least Squares. Difference: LS mean change from baseline in the Psychobiotics group minus LS mean change from baseline in the Placebo group. Refer to Supplementary Table S2.10. Estimates are adjusted for visit, group, baseline, gender, depression status, pain status, and cancer location, including visit-by-group and visit-by-baseline interactions.

## Data Availability

The data that support the findings of this study are available on request from the corresponding author, K.K.

## Supplementary Materials

The following supporting information can be downloaded at: https://www.mdpi.com/article/10.3390/nu18193254/s1, Table S1.1: HR-QoL: Physical Function [PF] Changes from Baseline, by Visit and Group; Table S1.2: HR-QoL: Role Physical [RP] Changes from Baseline, by Visit and Group; Table S1.3: HR-QoL: Bodily Pain [BP] Changes from Baseline, by Visit and Group; Table S1.4: HR-QoL: General Health [GH] Changes from Baseline, by Visit and Group; Table S1.5: HR-QoL: Vitality [VT] Changes from Baseline, by Visit and Group; Table S1.6: HR-QoL: Social Functioning [SF] Changes from Baseline, by Visit and Group; Table S1.7: HR-QoL: Role Emotional [RE] Changes from Baseline, by Visit and Group; Table S1.8: HR-QoL: Mental Health [MH] Changes from Baseline, by Visit and Group; Table S1.9: HR-QoL: Physical Component Summary [PCS] Changes from Baseline, by Visit and Group; Table S1.10: HR-QoL: Mental Component Summary [MCS] Changes from Baseline, by Visit and Group; Table S2.1: Physical Function [PF]: Mixed Model for Repeated Measures for the Change from Baseline; Table S2.2: Role Physical [RP]: Mixed Model for Repeated Measures for the Change from Baseline; Table S2.3: Bodily Pain [BP]: Mixed Model for Repeated Measures for the Change from Baseline; Table S2.4: General Health [GH]: Mixed Model for Repeated Measures for the Change from Baseline; Table S2.5: Vitality [VT]: Mixed Model for Repeated Measures for the Change from Baseline; Table S2.6: Social Functioning [SF]: Mixed Model for Repeated Measures for the Change from Baseline; Table S2.7: Role Emotional [RE]: Mixed Model for Repeated Measures for the Change from Baseline; Table S2.8: Mental Health [MH]: Mixed Model for Repeated Measures for the Change from Baseline; Table S2.9: Physical Component Summary [PCS]: Mixed Model for Repeated Measures for the Change from Baseline; Table S2.10: Mental Component Summary [MCS]: Mixed Model for Repeated Measures for the Change from Baseline; Table S3.1: Physical Function [PF]: Least Squares Mean Estimates for Change from Baseline by Visit and Group Across Covariate Levels; Table S3.2: Role Physical [RP]: Least Squares Mean Estimates for Change from Baseline by Visit and Group Across Covariate Levels; Table S3.3: Bodily Pain [BP]: Least Squares Mean Estimates for Change from Baseline by Visit and Group Across Covariate Levels; Table S3.4: General Health [GH]: Least Squares Mean Estimates for Change from Baseline by Visit and Group Across Covariate Levels; Table S3.5: Vitality [VT]: Least Squares Mean Estimates for Change from Baseline by Visit and Group Across Covariate Levels; Table S3.6: Social Functioning [SF]: Least Squares Mean Estimates for Change from Baseline by Visit and Group Across Covariate Levels; Table S3.7: Role Emotional [RE]: Least Squares Mean Estimates for Change from Baseline by Visit and Group Across Covariate Levels; Table S3.8: Mental Health [MH]: Least Squares Mean Estimates for Change from Baseline by Visit and Group Across Covariate Levels; Table S3.9: Physical Component Summary [PCS]: Least Squares Mean Estimates for Change from Baseline by Visit and Group Across Covariate Levels; Table S3.10: Mental Component Summary [MCS]: Least Squares Mean Estimates for Change from Baseline by Visit and Group Across Covariate Levels; Table S3.11: Baseline SF-36 domain and component summary scores by treatment group; Table S3.12: Participant numbers across categories included in the exploratory subgroup analyses.

## Abbreviations

The following abbreviations are used in this manuscript: HR-QoL: Health-Related Quality of Life; SF-36: Short Form-36 Health Survey.

## References

[1] Mansoor W., Joo S., Norquist J.M., Kato K., Sun J.M., Shah M.A., Enzinger P., Adenis A., Doi T., Kojima T. (2024). Health-related quality-of-life analysis from KEYNOTE-590: Pembrolizumab plus chemotherapy versus chemotherapy for advanced esophageal cancer. Oncologist.

[2] Marta G.N., Del Nero L.G., Marta G.N., Mangabeira A., Critchi G., Kovács M.J., Silva J.L., Saad E.D. (2014). Treatment priorities in oncology: Do we want to live longer or better?. Clinics.

[3] Kopec J.A., Yothers G., Ganz P.A., Land S.R., Cecchini R.S., Wieand H.S., Lembersky B.C., Wolmark N. (2007). Quality of life in operable colon cancer patients receiving oral compared with intravenous chemotherapy: Results from National Surgical Adjuvant Breast and Bowel Project Trial C-06. J. Clin. Oncol..

[4] Tanaka T., Morishita S., Nakano J., Inoue J., Okayama T., Suzuki K., Osaki K., Fukushima T. (2025). Relationship between patient-reported health-related quality of life as measured with the SF-36 or SF-12 and their mortality risk in patients with diverse cancer type: A meta-analysis. Int. J. Clin. Oncol..

[5] Theodoropoulos G.E., Karantanos T., Stamopoulos P., Zografos G. (2013). Prospective evaluation of health-related quality of life after laparoscopic colectomy for cancer. Tech. Coloproctol..

[6] Montazeri A. (2008). Health-related quality of life in breast cancer patients: A bibliographic review of the literature from 1974 to 2007. J. Exp. Clin. Cancer Res..

[7] Thong M.S.Y., Doege D., Weißer L., Koch-Gallenkamp L., Jansen L., Bertram H., Eberle A., Holleczek B., Nennecke A., Waldmann A. (2023). Persisting Deficits in Health-Related Quality of Life of Colorectal Cancer Survivors 14–24 Years Post-Diagnosis: A Population-Based Study. Curr. Oncol..

[8] Kunitake H., Russell M.M., Zheng P., Yothers G., Land S.R., Petersen L., Fehrenbacher L., Giguere J.K., Wickerham D.L., Ko C.Y. (2017). Quality of life and symptoms in long-term survivors of colorectal cancer: Results from NSABP protocol LTS-01. J. Cancer Surviv..

[9] Lau J., Peh C.H., Ng A., Koh W.L., Luo N., Tan K.K. (2025). Does adjuvant chemotherapy result in poorer health-related quality of life among colorectal cancer patients? A longitudinal multisite observational study in Singapore. Health Qual. Life Outcomes.

[10] Malik Y.G., Benth J., Hamre H.M., Faerden A.E., Ignjatovic D., Schultz J.K. (2024). Chemotherapy reduces long-term quality of life in recurrence-free colon cancer survivors (LaTE study)—A nationwide inverse probability of treatment-weighted registry-based cohort study and survey. Colorectal Dis..

[11] van Velzen M.J.M., Pape M., Sprangers M.A.G., van Kleef J.J., Mostert B., Beerepoot L.V., Slingerland M., Gootjes E.C., Hoekstra R., van de Poll-Franse L.V. (2024). Chemotherapy-Induced Peripheral Neuropathy in Patients With Gastroesophageal Cancer. J. Natl. Compr. Cancer Netw..

[12] Obulapuram R., Narapaka P.K., Esuru C., Singh M., Dhingra S. (2025). Association of GI symptoms with health-related quality of life in cancer patients undergoing chemotherapy: A prospective study. Support Care Cancer.

[13] Dahouri A., Sahebihagh M.H., Gilani N. (2024). Predictive factors associated with health-related quality of life in patients with colorectal cancer in Iran: A cross-sectional study. BMJ Open.

[14] Cryan J.F., Dinan T.G. (2012). Mind-altering microorganisms: The impact of the gut microbiota on brain and behaviour. Nat. Rev. Neurosci..

[15] Foster J.A., McVey Neufeld K.A. (2013). Gut-brain axis: How the microbiome influences anxiety and depression. Trends Neurosci..

[16] Sarkar A., Lehto S.M., Harty S., Dinan T.G., Cryan J.F., Burnet P.W.J. (2016). Psychobiotics and the Manipulation of Bacteria-Gut-Brain Signals. Trends Neurosci..

[17] Dinan T.G., Stanton C., Cryan J.F. (2013). Psychobiotics: A novel class of psychotropic. Biol. Psychiatry.

[18] Mörkl S., Butler M.I., Holl A., Cryan J.F., Dinan T.G. (2020). Probiotics and the Microbiota-Gut-Brain Axis: Focus on Psychiatry. Curr. Nutr. Rep..

[19] Andersson H., Tullberg C., Ahrné S., Hamberg K., Lazou Ahrén I., Molin G., Sonesson M., Håkansson Å. (2016). Oral Administration of Lactobacillus plantarum 299v Reduces Cortisol Levels in Human Saliva during Examination Induced Stress: A Randomized, Double-Blind Controlled Trial. Int. J. Microbiol..

[20] Morais L.H., Schreiber H.L.t., Mazmanian S.K. (2021). The gut microbiota-brain axis in behaviour and brain disorders. Nat. Rev. Microbiol..

[21] O’Mahony S.M., Clarke G., Borre Y.E., Dinan T.G., Cryan J.F. (2015). Serotonin, tryptophan metabolism and the brain-gut-microbiome axis. Behav. Brain Res..

[22] Breit S., Kupferberg A., Rogler G., Hasler G. (2018). Vagus Nerve as Modulator of the Brain-Gut Axis in Psychiatric and Inflammatory Disorders. Front. Psychiatry.

[23] Menni A.E., Theodorou H., Tzikos G., Theodorou I.M., Semertzidou E., Stelmach V., Shrewsbury A.D., Stavrou G., Kotzampassi K. (2025). Rewiring Mood: Precision Psychobiotics as Adjunct or Stand-Alone Therapy in Depression Using Insights from 19 Randomized Controlled Trials in Adults. Nutrients.

[24] Lee J.Y., Chu S.H., Jeon J.Y., Lee M.K., Park J.H., Lee D.C., Lee J.W., Kim N.K. (2014). Effects of 12 weeks of probiotic supplementation on quality of life in colorectal cancer survivors: A double-blind, randomized, placebo-controlled trial. Dig. Liver Dis..

[25] Zainal Abidin Z., Hein Z.M., Che Mohd Nassir C.M.N., Shari N., Che Ramli M.D. (2025). Pharmacological modulation of the gut-brain axis: Psychobiotics in focus for depression therapy. Front. Pharmacol..

[26] Khaleel S.M., Shanshal S.A., Khalaf M.M. (2023). The Role of Probiotics in Colorectal Cancer: A Review. J. Gastrointest. Cancer.

[27] Ait-Belgnaoui A., Payard I., Rolland C., Harkat C., Braniste V., Théodorou V., Tompkins T.A. (2018). Bifidobacterium longum and Lactobacillus helveticus Synergistically Suppress Stress-related Visceral Hypersensitivity Through Hypothalamic-Pituitary-Adrenal Axis Modulation. J. Neurogastroenterol. Motil..

[28] Day R., Friedman D., Cardoso A., Naghibi M., Pont A., Martinez-Blanch J., Lamelas A., Chenoll E., Kakilla C., Rea K. (2026). A Randomised, Double-Blind, Placebo-Controlled Trial of Probiotic and Postbiotic Strains in Healthy Adults with Self-Reported Anxiety: Effects on Mood, Vitality, Quality of Life and Perceived Stress. Brain Sci..

[29] Kelly J.R., Kennedy P.J., Cryan J.F., Dinan T.G., Clarke G., Hyland N.P. (2015). Breaking down the barriers: The gut microbiome, intestinal permeability and stress-related psychiatric disorders. Front. Cell. Neurosci..

[30] Deleemans J.M., Gajtani Z., Baydoun M., Reimer R.A., Piedalue K.A., Carlson L.E. (2021). The Use of Prebiotic and Probiotic Interventions for Treating Gastrointestinal and Psychosocial Health Symptoms in Cancer Patients and Survivors: A Systematic Review. Integr. Cancer Ther..

[31] Magalhães-Guedes K.T. (2022). Psychobiotic Therapy: Method to Reinforce the Immune System. Clin. Psychopharmacol. Neurosci..

[32] Strandwitz P. (2018). Neurotransmitter modulation by the gut microbiota. Brain Res..

[33] Park J.C., Chang L., Kwon H.K., Im S.H. (2025). Beyond the gut: Decoding the gut-immune-brain axis in health and disease. Cell. Mol. Immunol..

[34] Martin C.R., Osadchiy V., Kalani A., Mayer E.A. (2018). The Brain-Gut-Microbiome Axis. Cell. Mol. Gastroenterol. Hepatol..

[35] Tzikos G., Chamalidou E., Christopoulou D., Apostolopoulou A., Gkarmiri S., Pertsikapa M., Menni A.E., Theodorou I.M., Stavrou G., Doutsini N.D. (2025). Psychobiotics Ameliorate Depression and Anxiety Status in Surgical Oncology Patients: Results from the ProDeCa Study. Nutrients.

[36] Tzikos G., Menni A.E., Theodorou H., Chamalidou E., Theodorou I.M., Stavrou G., Shrewsbury A.D., Amaniti A., Konsta A., Tsetis J.K. (2025). Beyond Analgesia: Psychobiotics as an Adjunctive Approach to Pain Management in Gastrointestinal Oncology-A Post Hoc Analysis from the ProDeCa Study. Nutrients.

[37] Bjordal K., Mastekaasa A., Kaasa S. (1995). Self-reported satisfaction with life and physical health in long-term cancer survivors and a matched control group. Eur. J. Cancer B Oral. Oncol..

[38] Koller M., Klinkhammer-Schalke M., Lorenz W. (2005). Outcome and quality of life in medicine: A conceptual framework to put quality of life research into practice. Urol. Oncol..

[39] Ware J.E., Gandek B. (1998). Overview of the SF-36 Health Survey and the International Quality of Life Assessment (IQOLA) Project. J. Clin. Epidemiol..

[40] Garratt A., Schmidt L., Mackintosh A., Fitzpatrick R. (2002). Quality of life measurement: Bibliographic study of patient assessed health outcome measures. BMJ.

[41] Hernández-Segura N., Marcos-Delgado A., Pinto-Carral A., Fernández-Villa T., Molina A.J. (2022). Health-Related Quality of Life (HRQOL) Instruments and Mobility: A Systematic Review. Int. J. Environ. Res. Public Health.

[42] Barbos V., Feciche B., Latcu S., Croitor A., Dema V., Bardan R., Faur F.I., Mateescu T., Novacescu D., Bogdan G. (2024). The Assessment of SF-36 Survey for Quality-of-Life Measurement after Radical Cystectomy for Muscle-Invasive Bladder Cancer: A Systematic Review. Diseases.

[43] Ware J., Kosinski M.A., Dewey J. (2000). How to Score Version 2 of the SF-36^®^ Health Survey.

[44] Ware J.E., Kosinski M., Bayliss M.S., McHorney C.A., Rogers W.H., Raczek A. (1995). Comparison of Methods for the Scoring and Statistical Analysis of SF-36 Health Profile and Summary Measures: Summary of Results from the Medical Outcomes Study. Med. Care.

[45] Wu W., Qiu R. (2025). The Effect of Nursing-Based Enhanced Recovery After Surgery on Quality of Life in Lung Cancer Patients Receiving Surgery: A Systematic Review and Meta-Analysis. Nurs. Health Sci..

[46] Michael Y.L., Kawachi I., Berkman L.F., Holmes M.D., Colditz G.A. (2000). The persistent impact of breast carcinoma on functional health status: Prospective evidence from the Nurses’ Health Study. Cancer.

[47] Sweeney C., Schmitz K.H., Lazovich D., Virnig B.A., Wallace R.B., Folsom A.R. (2006). Functional limitations in elderly female cancer survivors. J. Natl. Cancer Inst..

[48] Phipps E., Braitman L.E., Stites S., Leighton J.C. (2008). Quality of life and symptom attribution in long-term colon cancer survivors. J. Eval. Clin. Pract..

[49] Vironen J.H., Kairaluoma M., Aalto A.M., Kellokumpu I.H. (2006). Impact of functional results on quality of life after rectal cancer surgery. Dis. Colon Rectum.

[50] Ho Y.H., Low D., Goh H.S. (1996). Bowel function survey after segmental colorectal resections. Dis. Colon Rectum.

[51] You Y.N., Chua H.K., Nelson H., Hassan I., Barnes S.A., Harrington J. (2008). Segmental vs. extended colectomy: Measurable differences in morbidity, function, and quality of life. Dis. Colon Rectum.

[52] Bregendahl S., Emmertsen K.J., Lous J., Laurberg S. (2013). Bowel dysfunction after low anterior resection with and without neoadjuvant therapy for rectal cancer: A population-based cross-sectional study. Colorectal Dis..

[53] Ketelaers S.H.J., van Heinsbergen M., Orsini R.G., Vogelaar F.J., Konsten J.L.M., Nieuwenhuijzen G.A.P., Rutten H.J.T., Burger J.W.A., Bloemen J.G. (2022). Functional Bowel Complaints and the Impact on Quality of Life After Colorectal Cancer Surgery in the Elderly. Front. Oncol..

[54] Sampsell K., Hao D., Reimer R.A. (2020). The Gut Microbiota: A Potential Gateway to Improved Health Outcomes in Breast Cancer Treatment and Survivorship. Int. J. Mol. Sci..

[55] Hattori Y., Fujii T., Tochio T., Kumamoto T., Hiro J., Matsuoka H., Masumori K., Funasaka K., Ohno E., Hirooka Y. (2025). Postoperative Gut Microbiota Changes after Colorectal Cancer Surgery: A Comparative Study Based on Resection Sites. J. Anus Rectum Colon.

[56] Kohn J., Troester A., Ziegert Z., Frebault J., Boatman S., Martell M., Nalluri-Butz H., Bobel M.C., Goffredo P., Johnson A.J. (2025). The Role of Surgical and Perioperative Factors in Shaping Gut Microbiome Recovery After Colorectal Surgery. Antibiotics.

[57] Xu L., Li X., Chen L., Ma H., Wang Y., Liu W., Liao A., Tan L., Gao X., Xiao W. (2025). Gut microbiome and plasma metabolome alterations in ileostomy and after closure of ileostomy. Microbiol. Spectr..

[58] Bajic J.E., Johnston I.N., Howarth G.S., Hutchinson M.R. (2018). From the Bottom-Up: Chemotherapy and Gut-Brain Axis Dysregulation. Front. Behav. Neurosci..

[59] Sonis S.T. (2004). The pathobiology of mucositis. Nat. Rev. Cancer.

[60] Touchefeu Y., Montassier E., Nieman K., Gastinne T., Potel G., Bruley des Varannes S., Le Vacon F., de La Cochetière M.F. (2014). Systematic review: The role of the gut microbiota in chemotherapy- or radiation-induced gastrointestinal mucositis—Current evidence and potential clinical applications. Aliment. Pharmacol. Ther..

[61] André T., Boni C., Mounedji-Boudiaf L., Navarro M., Tabernero J., Hickish T., Topham C., Zaninelli M., Clingan P., Bridgewater J. (2004). Oxaliplatin, fluorouracil, and leucovorin as adjuvant treatment for colon cancer. N. Engl. J. Med..

[62] Levinsen A.K.G., Dalton S.O., Jakobsen E., Gögenur I., Borre M., Zachariae R., Christensen P., Laurberg S., de Nully Brown P., Hölmich L.R. (2025). Prevalence of and factors associated with clinically important levels of fatigue, pain, and insomnia in survivors of cancer: A population-based cross-sectional study. J. Cancer Surviv..

[63] Wefel J.S., Saleeba A.K., Buzdar A.U., Meyers C.A. (2010). Acute and late onset cognitive dysfunction associated with chemotherapy in women with breast cancer. Cancer.

[64] von Gruenigen V.E., Huang H.Q., Gil K.M., Gibbons H.E., Monk B.J., Rose P.G., Armstrong D.K., Cella D., Wenzel L. (2010). A comparison of quality-of-life domains and clinical factors in ovarian cancer patients: A Gynecologic Oncology Group study. J. Pain Symptom Manag..

[65] Grant C.V., Loman B.R., Bailey M.T., Pyter L.M. (2021). Manipulations of the gut microbiome alter chemotherapy-induced inflammation and behavioral side effects in female mice. Brain Behav. Immun..

[66] Halle J.L., Zhang Q., Baumfalk D.R., Puppa M.J., Mohamed J.S., Glazer E.S., Smuder A.J., Alway S.E., Carson J.A. (2026). Sex impacts inflammatory signaling, body composition, and physical function in tumor-bearing mice receiving chemotherapy. Am. J. Physiol. Cell Physiol..

[67] Campbell A. (1976). Subjective measures of well-being. Am. Psychol..

[68] The WHOQOL Group (1995). The World Health Organization Quality of Life assessment (WHOQOL): Position paper from the World Health Organization. Soc. Sci. Med..

[69] Matsuyama R., Reddy S., Smith T.J. (2006). Why do patients choose chemotherapy near the end of life? A review of the perspective of those facing death from cancer. J. Clin. Oncol..

[70] McQuellon R.P., Muss H.B., Hoffman S.L., Russell G., Craven B., Yellen S.B. (1995). Patient preferences for treatment of metastatic breast cancer: A study of women with early-stage breast cancer. J. Clin. Oncol..

[71] Caro A., Olona C., Jiménez A., Olona M., Vicente V. (2012). Quality of life in patients operated on for digestive neoplasia: A prospective study. Colorectal Dis..

[72] Crawford E.D., Bennett C.L., Stone N.N., Knight S.J., DeAntoni E., Sharp L., Garnick M.B., Porterfield H.A. (1997). Comparison of perspectives on prostate cancer: Analyses of survey data. Urology.

[73] Wu H.S., Harden J.K. (2015). Symptom burden and quality of life in survivorship: A review of the literature. Cancer Nurs..

[74] Kristensen H., Thyø A., Christensen P. (2019). Systematic review of the impact of demographic and socioeconomic factors on quality of life in ostomized colorectal cancer survivors. Acta Oncol..

[75] Flyum I.R., Mahic S., Grov E.K., Joranger P. (2021). Health-related quality of life in patients with colorectal cancer in the palliative phase: A systematic review and meta-analysis. BMC Palliat. Care.

[76] Ciernikova S., Mego M., Chovanec M. (2021). Exploring the Potential Role of the Gut Microbiome in Chemotherapy-Induced Neurocognitive Disorders and Cardiovascular Toxicity. Cancers.

[77] Harms J., Kunzmann B., Bredereke J., Harms L., Jungbluth T., Zimmermann T. (2023). Anxiety in patients with gastrointestinal cancer undergoing primary surgery. J. Cancer Res. Clin. Oncol..

[78] Moldes-Moro R., de Dios-Duarte M.J. (2023). Colorectal Cancer Surgery: Influence of Psychosocial Factors. Cancers.

[79] Hasan E.M., Calma C.L., Tudor A., Oancea C., Tudorache V., Petrache I.A., Tudorache E., Papava I. (2021). Coping, Anxiety, and Pain Intensity in Patients Requiring Thoracic Surgery. J. Pers. Med..

[80] Halfaker D.A., Akeson S.T., Hathcock D.R., Mattson C., Wunderlich T.L. (2011). Psychological aspects of pain. Pain Procedures in Clinical Practice.

[81] Geisser M.E., Roth R.S., Theisen M.E., Robinson M.E., Riley J.L. (2000). Negative affect, self-report of depressive symptoms, and clinical depression: Relation to the experience of chronic pain. Clin. J. Pain.

[82] Zutshi M., Hull T., Shedda S., Lavery I., Hammel J. (2013). Gender differences in mortality, quality of life and function after restorative procedures for rectal cancer. Colorectal Dis..

[83] Hendren S.K., O’Connor B.I., Liu M., Asano T., Cohen Z., Swallow C.J., Macrae H.M., Gryfe R., McLeod R.S. (2005). Prevalence of male and female sexual dysfunction is high following surgery for rectal cancer. Ann. Surg..

[84] Daniels I.R., Woodward S., Taylor F.G., Raja A., Toomey P. (2006). Female urogenital dysfunction following total mesorectal excision for rectal cancer. World J. Surg. Oncol..

[85] Kristensen H., Thyø A., Emmertsen K.J., Smart N.J., Pinkney T., Warwick A.M., Pang D., Elfeki H., Shalaby M., Emile S.H. (2022). Surviving rectal cancer at the cost of a colostomy: Global survey of long-term health-related quality of life in 10 countries. BJS Open.

[86] Näsvall P., Dahlstrand U., Löwenmark T., Rutegård J., Gunnarsson U., Strigård K. (2017). Quality of life in patients with a permanent stoma after rectal cancer surgery. Qual. Life Res..

[87] Breen S.J., Baravelli C.M., Schofield P.E., Jefford M., Yates P.M., Aranda S.K. (2009). Is symptom burden a predictor of anxiety and depression in patients with cancer about to commence chemotherapy?. Med. J. Aust..

[88] Ferreira E.D., Barbosa M.H., Sonobe H.M., Barichello E. (2017). Self-esteem and health-related quality of life in ostomized patients. Rev. Bras. Enferm..

[89] Thompson T., Correll C.U., Gallop K., Vancampfort D., Stubbs B. (2016). Is Pain Perception Altered in People with Depression? A Systematic Review and Meta-Analysis of Experimental Pain Research. J. Pain.

[90] Rand K.L., Cripe L.D., Monahan P.O., Tong Y., Schmidt K., Rawl S.M. (2012). Illness appraisal, religious coping, and psychological responses in men with advanced cancer. Support Care Cancer.

[91] Tiselius C., Rosenblad A., Strand E., Smedh K. (2021). Risk factors for poor health-related quality of life in patients with colon cancer include stoma and smoking habits. Health Qual. Life Outcomes.

