# Supplementary material for: Longitudinal Trajectories of Physical and Mental Health-Related Quality of Life Following Psychobiotic Supplementation in Gastrointestinal Oncology Patients: A Post Hoc Analysis of the ProDeCa Study
Source: Nutrients. 2026 Oct 2;18(19):3254. doi: 10.3390/nu18193254 (PMC13666839; doi:10.3390/nu18193254)
Supplement: Supplementary file 1 [file nutrients-18-03254-s001.zip › nutrients-4535030-supplementary.pdf]

Tables S1

Table S1.1. HR-QoL: Physical Function [PF] Changes from Baseline, by Visit and Group

Group	Visit	Value	n	Mean	SD	Q1	Median	Q3	Min	Max
**Overall**	**Baseline**	Base	266	38.65	17.311	25.00	35.0	50.00	0.00	80.00
		Base	266	38.65	17.311	25.00	35.0	50.00	0.00	80.00
	**Visit 1**	Post	266	41.20	13.404	30.00	40.0	50.00	15.00	75.00
		Change	266	2.56	17.715	-10.00	5.0	15.00	-35.00	45.00
		Base	231	38.72	17.137	25.00	35.0	50.00	0.00	80.00
	**Visit 2**	Post	231	36.32	20.342	20.00	35.0	50.00	5.00	85.00
		Change	231	-2.40	29.642	-30.00	5.0	25.00	-55.00	60.00
**Placebo**	**Baseline**	Base	134	48.88	15.795	35.00	50.0	63.75	0.00	80.00
		Base	134	48.88	15.795	35.00	50.0	63.75	0.00	80.00
	**Visit 1**	Post	134	39.78	13.557	30.00	40.0	50.00	15.00	75.00
		Change	134	-9.10	15.142	-20.00	-10.0	0.00	-35.00	40.00
		Base	117	48.80	15.969	35.00	50.0	65.00	0.00	80.00
	**Visit 2**	Post	117	19.91	9.261	15.00	20.0	25.00	5.00	50.00
		Change	117	-28.89	13.836	-40.00	-30.0	-20.00	-55.00	10.00
**Psychobiotics**	**Baseline**	Base	132	28.26	11.705	20.00	25.0	35.00	10.00	60.00
		Base	132	28.26	11.705	20.00	25.0	35.00	10.00	60.00
	**Visit 1**	Post	132	42.65	13.141	30.00	42.5	55.00	15.00	70.00
		Change	132	14.39	11.035	10.00	15.0	20.00	-15.00	45.00
		Base	114	28.38	11.081	20.00	25.0	35.00	10.00	60.00
	**Visit 2**	Post	114	53.16	13.766	45.00	50.0	63.75	25.00	85.00
		Change	114	24.78	10.958	16.25	25.0	30.00	5.00	60.00

SD: Standard Deviation; Q1: 25th percentile; Q3: 75th percentile

Change= Post-Base

Table S1.2. HR-QoL: Role Physical [RP] Changes from Baseline, by Visit and Group

Group	Visit	Value	n	Mean	SD	Q1	Median	Q3	Min	Max
**Overall**	**Baseline**	Base	266	35.24	21.628	25.00	25.00	50.00	0.00	100.00
		Base	266	35.24	21.628	25.00	25.00	50.00	0.00	100.00
	**Visit 1**	Post	266	45.86	25.547	25.00	50.00	75.00	0.00	100.00
		Change	266	10.62	25.374	0.00	12.50	25.00	-50.00	75.00
		Base	231	34.74	21.299	25.00	25.00	50.00	0.00	100.00
	**Visit 2**	Post	231	39.72	32.047	25.00	25.00	75.00	0.00	100.00
		Change	231	4.98	29.984	-25.00	0.00	25.00	-75.00	75.00
**Placebo**	**Baseline**	Base	134	33.21	19.576	25.00	25.00	50.00	0.00	75.00
		Base	134	33.21	19.576	25.00	25.00	50.00	0.00	75.00
	**Visit 1**	Post	134	35.82	24.226	25.00	25.00	50.00	0.00	100.00
		Change	134	2.61	29.765	-25.00	0.00	25.00	-50.00	75.00
		Base	117	32.91	19.323	25.00	25.00	50.00	0.00	75.00
	**Visit 2**	Post	117	15.17	15.382	0.00	25.00	25.00	0.00	75.00
		Change	117	-17.74	19.984	-25.00	-25.00	0.00	-75.00	25.00
**Psychobiotics**	**Baseline**	Base	132	37.31	23.423	25.00	25.00	50.00	0.00	100.00
		Base	132	37.31	23.423	25.00	25.00	50.00	0.00	100.00
	**Visit 1**	Post	132	56.06	22.725	50.00	50.00	75.00	0.00	100.00
		Change	132	18.75	16.455	0.00	25.00	25.00	-25.00	50.00
		Base	114	36.62	23.086	25.00	25.00	50.00	0.00	100.00
	**Visit 2**	Post	114	64.91	24.149	50.00	75.00	75.00	25.00	100.00
		Change	114	28.29	18.372	25.00	25.00	50.00	0.00	75.00

SD: Standard Deviation; Q1: 25th percentile; Q3: 75th percentile

Change= Post-Base

Table S1.3. HR-QoL: Bodily Pain [BP] Changes from Baseline, by Visit and Group

Group	Visit	Value	n	Mean	SD	Q1	Median	Q3	Min	Max
**Overall**	**Baseline**	Base	266	45.00	20.005	30.00	45.00	57.50	0.00	100.00
		Base	266	45.00	20.005	30.00	45.00	57.50	0.00	100.00
	**Visit 1**	Post	266	51.94	17.574	42.50	52.50	65.00	10.00	100.00
		Change	266	6.94	19.992	-5.00	2.50	22.50	-45.00	80.00
		Base	232	45.16	20.281	30.00	45.00	57.50	0.00	100.00
	**Visit 2**	Post	232	50.79	18.101	40.00	51.25	65.00	10.00	100.00
		Change	232	5.62	24.910	-10.00	10.00	25.00	-57.50	57.50
**Placebo**	**Baseline**	Base	134	48.99	18.106	32.50	52.50	62.50	10.00	87.50
		Base	134	48.99	18.106	32.50	52.50	62.50	10.00	87.50
	**Visit 1**	Post	134	50.93	15.858	42.50	52.50	65.00	10.00	90.00
		Change	134	1.94	22.246	-10.00	0.00	12.50	-45.00	80.00
		Base	117	49.64	18.325	32.50	52.50	62.50	10.00	87.50
	**Visit 2**	Post	117	38.23	12.230	32.50	42.50	42.50	10.00	67.50
		Change	117	-11.41	19.947	-25.00	-10.00	7.50	-57.50	57.50
**Psychobiotics**	**Baseline**	Base	132	40.95	21.068	25.00	40.00	55.00	0.00	100.00
		Base	132	40.95	21.068	25.00	40.00	55.00	0.00	100.00
	**Visit 1**	Post	132	52.95	19.166	42.50	55.00	65.00	10.00	100.00
		Change	132	12.01	15.957	0.00	10.00	25.00	-20.00	42.50
		Base	115	40.61	21.220	25.00	40.00	52.50	0.00	100.00
	**Visit 2**	Post	115	63.57	13.605	55.00	65.00	68.75	35.00	100.00
		Change	115	22.96	15.862	12.50	22.50	33.75	-10.00	52.50

SD: Standard Deviation; Q1: 25th percentile; Q3: 75th percentile

Change= Post-Base

Table S1.4. HR-QoL: General Health [GH] Changes from Baseline, by Visit and Group

Group	Visit	Value	n	Mean	SD	Q1	Median	Q3	Min	Max
**Overall**	**Baseline**	Base	266	37.22	12.353	30.00	35.0	45.00	0.00	65.00
		Base	266	37.22	12.353	30.00	35.0	45.00	0.00	65.00
	**Visit 1**	Post	266	41.50	10.885	35.00	40.0	50.00	10.00	75.00
		Change	266	4.29	12.040	-5.00	5.0	10.00	-35.00	35.00
		Base	232	37.35	12.317	30.00	35.0	45.00	0.00	65.00
	**Visit 2**	Post	232	43.21	21.155	23.75	45.0	65.00	10.00	85.00
		Change	232	5.86	22.628	-15.00	10.0	25.00	-45.00	45.00
**Placebo**	**Baseline**	Base	134	37.01	12.716	30.00	35.0	45.00	15.00	65.00
		Base	134	37.01	12.716	30.00	35.0	45.00	15.00	65.00
	**Visit 1**	Post	134	37.46	8.985	30.00	35.0	45.00	10.00	60.00
		Change	134	0.45	13.785	-8.75	0.0	10.00	-35.00	30.00
		Base	117	37.39	12.415	30.00	35.0	45.00	15.00	65.00
	**Visit 2**	Post	117	24.27	9.452	20.00	25.0	30.00	10.00	50.00
		Change	117	-13.12	13.985	-25.00	-15.0	-5.00	-45.00	20.00
**Psychobiotics**	**Baseline**	Base	132	37.42	12.019	30.00	37.5	45.00	0.00	65.00
		Base	132	37.42	12.019	30.00	37.5	45.00	0.00	65.00
	**Visit 1**	Post	132	45.61	11.138	40.00	45.0	50.00	10.00	75.00
		Change	132	8.18	8.364	5.00	10.0	15.00	-15.00	35.00
		Base	115	37.30	12.270	30.00	40.0	45.00	0.00	65.00
	**Visit 2**	Post	115	62.48	8.568	55.00	65.0	65.00	40.00	85.00
		Change	115	25.17	9.618	20.00	25.0	30.00	10.00	45.00

SD: Standard Deviation; Q1: 25th percentile; Q3: 75th percentile

Change= Post-Base

Table S1.5. HR-QoL: Vitality [VT] Changes from Baseline, by Visit and Group

Group	Visit	Value	n	Mean	SD	Q1	Median	Q3	Min	Max
**Overall**	**Baseline**	Base	266	52.74	18.382	40.00	55.00	65.00	5.00	90.00
	**Visit 1**	Base	266	52.74	18.382	40.00	55.00	65.00	5.00	90.00
		Post	266	62.67	12.294	55.00	65.00	70.00	20.00	100.00
		Change	266	9.92	18.642	-5.00	10.00	20.00	-40.00	50.00
	**Visit 2**	Base	231	52.68	18.272	40.00	55.00	65.00	5.00	90.00
		Post	231	57.06	11.439	50.00	60.00	65.00	30.00	85.00
		Change	231	4.37	20.756	-10.00	5.00	15.00	-45.00	60.00
**Placebo**	**Baseline**	Base	134	60.00	13.847	50.00	65.00	70.00	30.00	85.00
	**Visit 1**	Base	134	60.00	13.847	50.00	65.00	70.00	30.00	85.00
		Post	134	63.88	10.143	56.25	65.00	70.00	35.00	90.00
		Change	134	3.88	17.639	-10.00	5.00	15.00	-40.00	40.00
	**Visit 2**	Base	117	59.74	14.185	50.00	65.00	70.00	30.00	85.00
		Post	117	50.17	8.782	45.00	50.00	55.00	30.00	80.00
		Change	117	-9.57	13.827	-20.00	-10.00	0.00	-45.00	20.00
**Psychobiotics**	**Baseline**	Base	132	45.38	19.504	35.00	45.00	60.00	5.00	90.00
	**Visit 1**	Base	132	45.38	19.504	35.00	45.00	60.00	5.00	90.00
		Post	132	61.44	14.082	50.00	65.00	70.00	20.00	100.00
		Change	132	16.06	17.659	5.00	15.00	25.00	-20.00	50.00
	**Visit 2**	Base	114	45.44	19.193	35.00	45.00	58.75	5.00	90.00
		Post	114	64.12	9.366	60.00	65.00	70.00	40.00	85.00
		Change	114	18.68	16.510	10.00	15.00	30.00	-15.00	60.00

SD: Standard Deviation; Q1: 25th percentile; Q3: 75th percentile

Change= Post-Base

Table S1.6 HR-QoL: Social Functioning [SF] Changes from Baseline, by Visit and Group

Group	Visit	Value	n	Mean	SD	Q1	Median	Q3	Min	Max
**Overall**	**Baseline**	Base	266	38.63	20.109	25.00	37.50	50.00	0.00	87.50
		Base	266	38.63	20.109	25.00	37.50	50.00	0.00	87.50
	**Visit 1**	Post	266	42.76	15.526	37.50	37.50	50.00	12.50	87.50
		Change	266	4.14	17.957	-12.50	12.50	12.50	-50.00	50.00
		Base	231	38.26	20.074	25.00	37.50	50.00	0.00	87.50
	**Visit 2**	Post	231	43.34	22.353	25.00	50.00	62.50	12.50	100.00
		Change	231	5.09	26.832	-12.50	0.00	25.00	-62.50	50.00
**Placebo**	**Baseline**	Base	134	39.74	19.259	25.00	37.50	50.00	0.00	75.00
		Base	134	39.74	19.259	25.00	37.50	50.00	0.00	75.00
	**Visit 1**	Post	134	38.62	13.664	25.00	37.50	50.00	12.50	75.00
		Change	134	-1.12	20.072	-12.50	0.00	12.50	-50.00	50.00
		Base	117	40.17	19.821	25.00	37.50	50.00	0.00	75.00
	**Visit 2**	Post	117	25.43	12.706	12.50	25.00	25.00	12.50	62.50
		Change	117	-14.74	19.601	-25.00	-12.50	0.00	-62.50	25.00
**Psychobiotics**	**Baseline**	Base	132	37.50	20.951	25.00	37.50	50.00	0.00	87.50
		Base	132	37.50	20.951	25.00	37.50	50.00	0.00	87.50
	**Visit 1**	Post	132	46.97	16.208	37.50	50.00	62.50	12.50	87.50
		Change	132	9.47	13.651	0.00	12.50	12.50	-25.00	37.50
		Base	114	36.29	20.229	25.00	37.50	50.00	0.00	87.50
	**Visit 2**	Post	114	61.73	13.333	50.00	62.50	62.50	37.50	100.00
		Change	114	25.44	15.682	12.50	25.00	37.50	-12.50	50.00

SD: Standard Deviation; Q1: 25th percentile; Q3: 75th percentile

Change= Post-Base

Table S1.7 HR-QoL: Role Emotional [RE] Changes from Baseline, by Visit and Group

Group	Visit	Value	n	Mean	SD	Q1	Median	Q3	Min	Max
**Overall**	**Baseline**	Base	266	37.84	26.472	33.33	33.33	66.67	0.00	100.00
		Base	266	37.84	26.472	33.33	33.33	66.67	0.00	100.00
	**Visit 1**	Post	266	44.86	27.789	33.33	33.33	66.67	0.00	100.00
		Change	266	7.02	30.386	0.00	0.00	33.33	-66.67	66.67
		Base	231	37.66	26.567	33.33	33.33	66.67	0.00	100.00
	**Visit 2**	Post	231	41.70	30.091	33.33	33.33	66.67	0.00	100.00
		Change	231	4.04	34.516	-33.33	0.00	33.33	-66.67	100.00
**Placebo**	**Baseline**	Base	134	38.81	27.479	33.33	33.33	66.67	0.00	100.00
		Base	134	38.81	27.479	33.33	33.33	66.67	0.00	100.00
	**Visit 1**	Post	134	36.82	27.804	8.33	33.33	66.67	0.00	100.00
		Change	134	-1.99	35.579	-33.33	0.00	33.33	-66.67	66.67
		Base	117	38.75	27.667	33.33	33.33	66.67	0.00	100.00
	**Visit 2**	Post	117	19.66	20.131	0.00	33.33	33.33	0.00	66.67
		Change	117	-19.09	26.011	-33.33	-33.33	0.00	-66.67	33.33
**Psychobiotics**	**Baseline**	Base	132	36.87	25.475	33.33	33.33	66.67	0.00	100.00
		Base	132	36.87	25.475	33.33	33.33	66.67	0.00	100.00
	**Visit 1**	Post	132	53.03	25.374	33.33	66.67	66.67	0.00	100.00
		Change	132	16.16	20.380	0.00	0.00	33.33	0.00	66.67
		Base	114	36.55	25.463	33.33	33.33	66.67	0.00	100.00
	**Visit 2**	Post	114	64.33	20.186	66.67	66.67	66.67	33.33	100.00
		Change	114	27.78	24.658	0.00	33.33	33.33	0.00	100.00

SD: Standard Deviation; Q1: 25th percentile; Q3: 75th percentile

Change= Post-Base

Table S1.8. HR-QoL: Mental Health [MH] Changes from Baseline, by Visit and Group

Group	Visit	Value	n	Mean	SD	Q1	Median	Q3	Min	Max
**Overall**	**Baseline**	Base	266	52.72	15.771	44.00	56.00	64.00	12.00	80.00
		Base	266	52.72	15.771	44.00	56.00	64.00	12.00	80.00
	**Visit 1**	Post	266	63.88	11.440	56.00	64.00	72.00	28.00	92.00
		Change	266	11.16	15.518	0.00	12.00	20.00	-20.00	48.00
		Base	231	51.95	15.717	44.00	52.00	64.00	12.00	80.00
	**Visit 2**	Post	231	58.81	10.991	52.00	60.00	68.00	36.00	84.00
		Change	231	6.86	17.525	-4.00	4.00	18.00	-24.00	56.00
**Placebo**	**Baseline**	Base	134	58.27	10.480	49.00	60.00	68.00	32.00	76.00
		Base	134	58.27	10.480	49.00	60.00	68.00	32.00	76.00
	**Visit 1**	Post	134	65.37	8.679	60.00	68.00	72.00	40.00	84.00
		Change	134	7.10	14.043	-4.00	4.00	16.00	-20.00	44.00
		Base	117	57.47	10.473	48.00	60.00	64.00	32.00	76.00
	**Visit 2**	Post	117	52.85	8.499	48.00	52.00	60.00	36.00	76.00
		Change	117	-4.62	12.167	-12.00	-4.00	4.00	-24.00	28.00
**Psychobiotics**	**Baseline**	Base	132	47.09	18.116	36.00	48.00	64.00	12.00	80.00
		Base	132	47.09	18.116	36.00	48.00	64.00	12.00	80.00
	**Visit 1**	Post	132	62.36	13.553	52.00	64.00	72.00	28.00	92.00
		Change	132	15.27	15.907	4.00	12.00	28.00	-16.00	48.00
		Base	114	46.28	18.062	33.00	46.00	60.00	12.00	80.00
	**Visit 2**	Post	114	64.91	9.870	60.00	68.00	72.00	36.00	84.00
		Change	114	18.63	14.037	8.00	16.00	28.00	-4.00	56.00

SD: Standard Deviation; Q1: 25th percentile; Q3: 75th percentile

Change= Post-Base

Table S1.9. HR-QoL: Physical Component Summary [PCS] Changes from Baseline, by Visit and Group

Group	Visit	Value	n	Mean	SD	Q1	Median	Q3	Min	Max
**Overall**	**Baseline**	Base	266	33.56	5.210	29.84	33.73	37.02	20.32	47.09
		Base	266	33.56	5.210	29.84	33.73	37.02	20.32	47.09
	**Visit 1**	Post	266	34.95	5.471	31.16	34.64	38.83	22.87	47.26
		Change	266	1.39	5.998	-2.33	1.61	6.02	-13.74	15.42
		Base	231	33.67	5.294	30.16	33.74	37.28	20.32	47.09
	**Visit 2**	Post	231	34.17	8.715	26.11	33.46	41.43	17.56	53.66
		Change	231	0.50	10.433	-8.73	1.90	10.19	-18.54	21.49
**Placebo**	**Baseline**	Base	134	35.12	4.829	32.23	34.93	38.67	22.94	47.09
		Base	134	35.12	4.829	32.23	34.93	38.67	22.94	47.09
	**Visit 1**	Post	134	33.32	5.096	29.97	33.19	35.80	22.87	45.53
		Change	134	-1.80	6.035	-6.50	-1.91	2.53	-13.74	15.42
		Base	117	35.30	4.886	32.31	35.02	38.81	22.94	47.09
	**Visit 2**	Post	117	26.54	3.625	24.21	26.26	28.95	17.56	35.29
		Change	117	-8.76	4.836	-12.30	-8.73	-5.43	-18.54	3.38
**Psychobiotics**	**Baseline**	Base	132	31.98	5.122	28.24	32.41	35.55	20.32	45.34
		Base	132	31.98	5.122	28.24	32.41	35.55	20.32	45.34
	**Visit 1**	Post	132	36.60	5.359	32.92	36.26	41.00	26.00	47.26
		Change	132	4.62	3.859	1.41	5.38	7.28	-3.15	11.53
		Base	114	31.98	5.187	28.16	32.68	35.65	20.32	45.34
	**Visit 2**	Post	114	41.99	4.381	38.89	41.48	44.89	28.54	53.66
		Change	114	10.00	4.195	6.80	10.37	12.80	1.59	21.49

SD: Standard Deviation; Q1: 25th percentile; Q3: 75th percentile

Table S1.10. HR-QoL: Mental Component Summary [MCS] Changes from Baseline, by Visit and Group

Group	Visit	Value	n	Mean	SD	Q1	Median	Q3	Min	Max
**Overall**	**Baseline**	Base	266	39.80	6.886	35.26	40.29	44.94	19.28	53.98
		Base	266	39.80	6.886	35.26	40.29	44.94	19.28	53.98
	**Visit 1**	Post	266	44.39	5.862	40.09	44.50	48.43	27.09	56.84
		Change	266	4.58	6.646	0.18	4.38	9.33	-12.32	20.27
		Base	231	39.52	6.975	34.83	39.66	44.80	19.28	53.98
	**Visit 2**	Post	231	42.79	6.015	37.85	43.38	47.21	28.61	58.87
		Change	231	3.27	8.105	-2.73	3.14	8.97	-18.25	22.39
**Placebo**	**Baseline**	Base	134	41.26	5.531	36.95	41.25	45.02	29.52	52.91
		Base	134	41.26	5.531	36.95	41.25	45.02	29.52	52.91
	**Visit 1**	Post	134	43.95	5.550	39.70	44.04	47.51	30.73	56.84
		Change	134	2.69	6.849	-2.29	2.49	7.31	-12.32	16.49
		Base	117	41.05	5.688	36.58	41.20	44.96	29.52	52.91
	**Visit 2**	Post	117	38.57	4.318	36.14	38.23	40.98	28.61	49.21
		Change	117	-2.48	5.169	-5.77	-2.66	1.35	-18.25	8.27
**Psychobiotics**	**Baseline**	Base	132	38.32	7.778	32.87	39.28	43.83	19.28	53.98
		Base	132	38.32	7.778	32.87	39.28	43.83	19.28	53.98
	**Visit 1**	Post	132	44.83	6.152	40.24	45.35	49.19	27.09	56.79
		Change	132	6.50	5.861	2.12	5.81	10.73	-4.70	20.27
		Base	114	37.95	7.805	32.04	37.89	43.55	19.28	53.98
	**Visit 2**	Post	114	47.12	4.134	44.84	46.94	49.80	34.44	58.87
		Change	114	9.17	6.073	5.19	9.22	13.48	-3.45	22.39

SD: Standard Deviation; Q1: 25th percentile; Q3: 75th percentile

Tables S2

Table S2.1. Physical Function [PF]: Mixed Model for Repeated Measures for the Change from Baseline

Covariate	Estimate	Standard Error	p-value
Intercept	27.414	1.8909	<0.001
Visit			
V1 (reference category)			
V2	12.255	2.0265	<0.001
Group			
Psychobiotics (reference category)			
Placebo	-13.579	1.6533	<0.001
Baseline PF	-0.486	0.0480	<0.001
Gender			
Male (reference category)			
Female	-0.446	1.1915	0.708
Depression			
No (reference category)			
Yes	1.914	1.1385	0.094
Pain			
No (reference category)			
Yes	1.015	1.1641	0.384
Cancer Location			
Colon-Rectum (reference category)			
Other	-0.291	1.1651	0.803
Visit * Group			
V1 * Psychobiotics (reference category)			
V2 * Placebo	-29.064	2.0186	<0.001
Visit * Baseline PF			
V1 * Baseline PF (reference category)			
V2 * Baseline PF	-0.062	0.0588	0.297

Mixed Model for Repeated Measures with change from baseline in PF as dependent variable. Model independent covariates include visit, group, baseline PF, gender, presence of depression, presence of pain, cancer location, group by visit interaction and baseline PF by visit interaction. Patients were fitted at random and an unstructured covariance matrix was used.

Table S2.2. Role Physical [RP]: Mixed Model for Repeated Measures for the Change from Baseline

Covariate	Estimate	Standard Error	p-value
Intercept	39.150	3.0457	<0.001
Visit			
V1(reference category)			
V2	6.272	3.6272	0.085
Group			
Psychobiotics (reference category)			
Placebo	-18.598	2.3624	<0.001
Baseline RP	-0.536	0.0550	<0.001
Gender			
Male (reference category)			
Female	-1.860	1.9671	0.345
Depression			
No (reference category)			
Yes	2.663	1.9095	0.164
Pain			
No (reference category)			
Yes	3.555	1.9219	0.066
Cancer Location			
Colon-Rectum (reference category)			
Other	-2.983	1.9392	0.125
Visit*Group			
V1 * Psychobiotics (reference category)			
V2 * Placebo	-28.977	3.2510	<0.001
Visit * Baseline RP			
V1 * Baseline RP (reference category)			
V2 * Baseline RP	0.076	0.0760	0.321

Mixed Model for Repeated Measures with change from baseline in RP as dependent variable. Model independent covariates include visit, group, baseline RP, gender, presence of depression, presence of pain, cancer location, group by visit interaction and baseline RP by visit interaction. Patients were fitted at random and an unstructured covariance matrix was used.

Table S2.3 Bodily Pain [BP]: Mixed Model for Repeated Measures for the Change from Baseline

Covariate	Estimate	Standard Error	p-value
Intercept	33.586	2.4679	<0.001
Visit			
V1(reference category)			
V2	15.351	2.2676	<0.001
Group			
Psychobiotics (reference category)			
Placebo	-5.301	1.7340	0.002
Baseline BP	-0.584	0.0439	<0.001
Gender			
Male (reference category)			
Female	3.063	1.6072	0.058
Depression			
No (reference category)			
Yes	2.516	1.5588	0.108
Pain			
No (reference category)			
Yes	-1.619	1.5996	0.312
Cancer Location			
Colon-Rectum (reference category)			
Other	1.479	1.5895	0.353
Visit*Group			
V1 * Psychobiotics (reference category)			
V2 * Placebo	-22.746	1.8537	<0.001
Visit * Baseline BP			
V1 * Baseline BP (reference category)			
V2 * Baseline BP	-0.113	0.0459	0.015

Mixed Model for Repeated Measures with change from baseline in BP as dependent variable. Model independent covariates include visit, group, baseline BP, gender, presence of depression, presence of pain, cancer location, group by visit interaction and baseline BP by visit interaction. Patients were fitted at random and an unstructured covariance matrix was use.

Table S2.4. General Health [GH]: Mixed Model for Repeated Measures for the Change from Baseline

Covariate	Estimate	Standard Error	p-value
Intercept	28.900	2.0290	<0.001
Visit			
V1(reference category)			
V2	20.666	1.7174	<0.001
Group			
Psychobiotics (reference category)			
Placebo	-8.072	1.0489	<0.001
Baseline GH	-0.584	0.0433	<0.001
Gender			
Male (reference category)			
Female	0.304	1.0146	0.765
Depression			
No (reference category)			
Yes	1.433	0.9839	0.147
Pain			
No (reference category)			
Yes	1.208	1.0198	0.237
Cancer Location			
Colon-Rectum (reference category)			
Other	0.141	1.0046	0.888
Visit*Group			
V1 * Psychobiotics (reference category)			
V2 * Placebo	-30.349	1.0249	<0.001
Visit * Baseline GH			
V1 * Baseline GH (reference category)			
V2 * Baseline GH	-0.101	0.0417	0.016

Mixed Model for Repeated Measures with change from baseline in GH as dependent variable. Model independent covariates include visit, group, baseline GH, gender, presence of depression, presence of pain, cancer location, group by visit interaction and baseline GH by visit interaction. Patients were fitted at random and an unstructured covariance matrix was use.

Table S2.5.Vitality [VT]: Mixed Model for Repeated Measures for the Change from Baseline

Covariate	Estimate	Standard Error	p-value
Intercept	50.585	2.1024	<0.001
Visit			
V1(reference category)			
V2	1.596	2.0453	0.436
Group			
Psychobiotics (reference category)			
Placebo	-0.493	1.3358	0.712
Baseline VT	-0.799	0.0365	<0.001
Gender			
Male (reference category)			
Female	-1.588	1.1504	0.169
Depression			
No (reference category)			
Yes	4.654	1.1092	<0.001
Pain			
No (reference category)			
Yes	-1.724	1.1281	0.128
Cancer Location			
Colon-Rectum (reference category)			
Other	1.749	1.1351	0.125
Visit*Group			
V1 * Psychobiotics (reference category)			
V2 * Placebo	-16.804	1.4557	<0.001
Visit * Baseline VT			
V1 * Baseline VT (reference category)			
V2 * Baseline VT	0.025	0.0399	0.538

Mixed Model for Repeated Measures with change from baseline in VT as dependent variable. Model independent covariates include visit, group, baseline VT, gender, presence of depression, presence of pain, cancer location, group by visit interaction and baseline VT by visit interaction. Patients were fitted at random and an unstructured covariance matrix was used.

Table S2.6. Social Functioning [SF]: Mixed Model for Repeated Measures for the Change from Baseline

Covariate	Estimate	Standard Error	p-value
Intercept	32.052	2.1300	<0.001
Visit			
V1(reference category)			
V2	18.707	1.8651	<0.001
Group			
Psychobiotics (reference category)			
Placebo	-9.272	1.4363	<0.001
Baseline SF	-0.600	0.0366	<0.001
Gender			
Male (reference category)			
Female	5.177	1.3309	<0.001
Depression			
No (reference category)			
Yes	-0.371	1.3195	0.779
Pain			
No (reference category)			
Yes	1.224	1.3092	0.351
Cancer Location			
Colon-Rectum (reference category)			
Other	-3.181	1.3213	0.017
Visit*Group			
V1 * Psychobiotics (reference category)			
V2 * Placebo	-28.371	1.6139	<0.001
Visit * Baseline SF			
V1 * Baseline SF (reference category)			
V2 * Baseline SF	-0.090	0.0403	0.026

Mixed Model for Repeated Measures with change from baseline in SF as dependent variable. Model independent covariates include visit, group, baseline SF, gender, presence of depression, presence of pain, cancer location, group by visit interact ion and baseline SF by visit interaction. Patients were fitted at random and an unstructured covariance matrix was use

Table S2.7. Role Emotional [RE]: Mixed Model for Repeated Measures for the Change from Baseline

Covariate	Estimate	Standard Error	p-value
Intercept	37.355	3.1201	<0.001
Visit			
V1(reference category)			
V2	13.935	3.8533	<0.001
Group			
Psychobiotics (reference category)			
Placebo	-17.169	2.6499	<0.001
Baseline RE	-0.581	0.0505	<0.001
Gender			
Male (reference category)			
Female	-1.418	2.0882	0.498
Depression			
No (reference category)			
Yes	6.198	2.0221	0.002
Pain			
No (reference category)			
Yes	0.727	2.0601	0.724
Cancer Location			
Colon-Rectum (reference category)			
Other	-2.766	2.0871	0.186
Visit*Group			
V1 * Psychobiotics (reference category)			
V2 * Placebo	-28.266	3.8833	<0.001
Visit * Baseline RE			
V1 * Baseline RE (reference category)			
V2 * Baseline RE	-0.072	0.0733	0.325

Mixed Model for Repeated Measures with change from baseline in RE as dependent variable. Model independent covariates include visit, group, baseline RE, gender, presence of depression, presence of pain, cancer location, group by visit interaction and baseline RE by visit interaction. Patients were fitted at random and an unstructured covariance matrix was used

Table S2.8. Mental Health [MH]: Mixed Model for Repeated Measures for the Change from Baseline

**Covariate**	**Estimate**	**Standard Error**	**p-value**
Intercept	49.653	2.1457	<0.001
Visit			
V1(reference category)			
V2	1.745	2.1180	0.411
Group			
Psychobiotics (reference category)			
Placebo	-0.160	1.2540	0.898
Baseline EWB	-0.714	0.0403	<0.001
Gender			
Male (reference category)			
Female	-1.336	1.1230	0.235
Depression			
No (reference category)			
Yes	-1.907	1.0792	0.078
Pain			
No (reference category)			
Yes	-0.139	1.0939	0.899
Cancer Location			
Colon-Rectum (reference category)			
Other	0.632	1.1112	0.570
Visit*Group			
V1 * Psychobiotics (reference category)			
V2 * Placebo	-15.412	1.3057	<0.001
Visit * Baseline EWB			
V1 * Baseline EWB (reference category)			
V2 * Baseline EWB	0.022	0.0416	0.595

Mixed Model for Repeated Measures with change from baseline in EWB as dependent variable. Model independent covariates include visit, group, baseline EWB, gender, presence of depression, presence of pain, cancer location, group by visit interaction and baseline EWB by visit interaction. Patients were fitted at random and an unstructured covariance matrix was use

Table S2.9. Physical Component Summary [PCS]: Mixed Model for Repeated Measures for the Change from Baseline

Covariate	Estimate	Standard Error	p-value
Intercept	19.124	1.6847	<0.001
Visit			
V1(reference category)			
V2	9.244	2.1306	<0.001
Group			
Psychobiotics (reference category)			
Placebo	-4.960	0.5190	<0.001
Baseline PCS	-0.477	0.0502	<0.001
Gender			
Male (reference category)			
Female	0.547	0.4255	0.200
Depression			
No (reference category)			
Yes	1.062	0.4138	0.011
Pain			
No (reference category)			
Yes	0.380	0.4176	0.364
Cancer Location			
Colon-Rectum (reference category)			
Other	0.100	0.4232	0.814
Visit*Group			
V1 * Psychobiotics (reference category)			
V2 * Placebo	-11.825	0.6834	<0.001
Visit * Baseline PCS			
V1 * Baseline PCS (reference category)			
V2 * Baseline PCS	-0.120	0.0650	0.065

Mixed Model for Repeated Measures with change from baseline in PCS as dependent variable. Model independent covariates include visit, group, baseline PCS, gender, presence of depression, presence of pain, cancer location, group by visit interaction and baseline PCS by visit interaction. Patients were fitted at random and an unstructured covariance matrix was used

Table S2.10. Mental Component Summary [MCS]: Mixed Model for Repeated Measures for the Change from Baseline

Covariate	Estimate	Standard Error	p-value
Intercept	28.373	1.6522	<0.001
Visit			
V1(reference category)			
V2	5.355	2.0273	0.009
Group			
Psychobiotics (reference category)			
Placebo	-2.155	0.5592	<0.001
Baseline MCS	-0.564	0.0410	<0.001
Gender			
Male (reference category)			
Female	-0.206	0.4765	0.666
Depression			
No (reference category)			
Yes	0.325	0.4630	0.483
Pain			
No (reference category)			
Yes	-0.032	0.4698	0.946
Cancer Location			
Colon-Rectum (reference category)			
Other	-0.455	0.4761	0.34
Visit*Group			
V1 * Psychobiotics (reference category)			
V2 * Placebo	-7.506	0.7156	<0.001
Visit * Baseline MCS			
V1 * Baseline MCS (reference category)			
V2 * Baseline MCS	-0.077	0.0516	0.137

Mixed Model for Repeated Measures with change from baseline in MCS as dependent variable. Model independent covariates include visit, group, baseline MCS, gender, presence of depression, presence of pain, cancer location, group by visit interaction and baseline MCS by visit interaction. Patients were fitted at random and an unstructured covariance matrix was used

Tables 3S

Table S3.1. Physical Function [PF]: Least Squares Mean Estimates for Change from Baseline by Visit and Group Across Covariate Levels

							95% Confidence Interval	
Visit	Group	Gender	Depression	Pain	Cancer Location	LS Mean	Lower Bound	Upper Bound
V1	Psychobiotics	Male	No	No	Colon-Rectum	8.63	5.80	11.45
V1	Placebo	Male	No	No	Colon-Rectum	-4.95	-7.77	-2.13
V2	Psychobiotics	Male	No	No	Colon-Rectum	18.50	15.58	21.43
V2	Placebo	Male	No	No	Colon-Rectum	-24.14	-27.06	-21.22
V1	Psychobiotics	Female	No	No	Colon-Rectum	8.18	5.15	11.21
V1	Placebo	Female	No	No	Colon-Rectum	-5.40	-8.62	-2.17
V2	Psychobiotics	Female	No	No	Colon-Rectum	18.06	14.94	21.17
V2	Placebo	Female	No	No	Colon-Rectum	-24.59	-27.92	-21.25
V1	Psychobiotics	Male	Yes	No	Colon-Rectum	10.54	7.45	13.63
V1	Placebo	Male	Yes	No	Colon-Rectum	-3.04	-6.08	0.00
V2	Psychobiotics	Male	Yes	No	Colon-Rectum	20.42	17.25	23.59
V2	Placebo	Male	Yes	No	Colon-Rectum	-22.23	-25.35	-19.10
V1	Psychobiotics	Female	Yes	No	Colon-Rectum	10.10	6.64	13.55
V1	Placebo	Female	Yes	No	Colon-Rectum	-3.48	-7.07	0.11
V2	Psychobiotics	Female	Yes	No	Colon-Rectum	19.97	16.45	23.49
V2	Placebo	Female	Yes	No	Colon-Rectum	-22.67	-26.36	-18.99
V1	Psychobiotics	Male	No	Yes	Colon-Rectum	9.64	6.20	13.09
V1	Placebo	Male	No	Yes	Colon-Rectum	-3.94	-7.16	-0.71
V2	Psychobiotics	Male	No	Yes	Colon-Rectum	19.52	16.00	23.04
V2	Placebo	Male	No	Yes	Colon-Rectum	-23.13	-26.46	-19.80
V1	Psychobiotics	Female	No	Yes	Colon-Rectum	9.20	5.77	12.62
V1	Placebo	Female	No	Yes	Colon-Rectum	-4.38	-7.78	-0.99
V2	Psychobiotics	Female	No	Yes	Colon-Rectum	19.07	15.57	22.57
V2	Placebo	Female	No	Yes	Colon-Rectum	-23.57	-27.09	-20.05
V1	Psychobiotics	Male	Yes	Yes	Colon-Rectum	11.56	7.97	15.14
V1	Placebo	Male	Yes	Yes	Colon-Rectum	-2.02	-5.35	1.31
V2	Psychobiotics	Male	Yes	Yes	Colon-Rectum	21.43	17.78	25.08
V2	Placebo	Male	Yes	Yes	Colon-Rectum	-21.21	-24.64	-17.79
V1	Psychobiotics	Female	Yes	Yes	Colon-Rectum	11.11	7.38	14.84
V1	Placebo	Female	Yes	Yes	Colon-Rectum	-2.47	-6.13	1.20
V2	Psychobiotics	Female	Yes	Yes	Colon-Rectum	20.98	17.19	24.78
V2	Placebo	Female	Yes	Yes	Colon-Rectum	-21.66	-25.43	-17.89
V1	Psychobiotics	Male	No	No	Other	8.34	5.68	10.99
V1	Placebo	Male	No	No	Other	-5.24	-7.94	-2.55
V2	Psychobiotics	Male	No	No	Other	18.21	15.43	20.99
V2	Placebo	Male	No	No	Other	-24.43	-27.21	-21.66
V1	Psychobiotics	Female	No	No	Other	7.89	5.00	10.78
V1	Placebo	Female	No	No	Other	-5.69	-8.82	-2.56
V2	Psychobiotics	Female	No	No	Other	17.77	14.76	20.77

Visit	Group	Gender	Depression	Pain	Cancer Location	LS Mean	95% Confidence Interval	
							Lower Bound	Upper Bound
V2	Placebo	Female	No	No	Other	-24.88	-28.11	-21.65
V1	Psychobiotics	Male	Yes	No	Other	10.25	7.40	13.10
V1	Placebo	Male	Yes	No	Other	-3.33	-6.17	-0.49
V2	Psychobiotics	Male	Yes	No	Other	20.13	17.17	23.08
V2	Placebo	Male	Yes	No	Other	-22.52	-25.43	-19.61
V1	Psychobiotics	Female	Yes	No	Other	9.80	6.55	13.06
V1	Placebo	Female	Yes	No	Other	-3.77	-7.21	-0.34
V2	Psychobiotics	Female	Yes	No	Other	19.68	16.33	23.03
V2	Placebo	Female	Yes	No	Other	-22.96	-26.48	-19.45
V1	Psychobiotics	Male	No	Yes	Other	9.35	6.33	12.38
V1	Placebo	Male	No	Yes	Other	-4.23	-7.04	-1.41
V2	Psychobiotics	Male	No	Yes	Other	19.23	16.09	22.36
V2	Placebo	Male	No	Yes	Other	-23.42	-26.33	-20.50
V1	Psychobiotics	Female	No	Yes	Other	8.90	5.88	11.93
V1	Placebo	Female	No	Yes	Other	-4.67	-7.70	-1.64
V2	Psychobiotics	Female	No	Yes	Other	18.78	15.65	21.91
V2	Placebo	Female	No	Yes	Other	-23.86	-27.01	-20.72
V1	Psychobiotics	Male	Yes	Yes	Other	11.27	8.16	14.37
V1	Placebo	Male	Yes	Yes	Other	-2.31	-5.17	0.54
V2	Psychobiotics	Male	Yes	Yes	Other	21.14	17.94	24.34
V2	Placebo	Male	Yes	Yes	Other	-21.50	-24.45	-18.56
V1	Psychobiotics	Female	Yes	Yes	Other	10.82	7.52	14.11
V1	Placebo	Female	Yes	Yes	Other	-2.76	-6.02	0.50
V2	Psychobiotics	Female	Yes	Yes	Other	20.69	17.31	24.08
V2	Placebo	Female	Yes	Yes	Other	-21.95	-25.31	-18.59

LS: Least Squares; Reference Table 1.2S

Table S3.2 Role Physical [RP]: Least Squares Mean Estimates for Change from Baseline by Visit and Group Across Covariate Levels

							95% Confidence Interval	
Visit	Group	Gender	Depression	Pain	Cancer Location	LS Mean	Lower Bound	Upper Bound
V1	Psychobiotics	Male	No	No	Colon-Rectum	20.37	15.83	24.91
V1	Placebo	Male	No	No	Colon-Rectum	1.77	-2.83	6.37
V2	Psychobiotics	Male	No	No	Colon-Rectum	29.29	24.58	34.00
V2	Placebo	Male	No	No	Colon-Rectum	-18.29	-23.05	-13.53
V1	Psychobiotics	Female	No	No	Colon-Rectum	18.51	13.48	23.54
V1	Placebo	Female	No	No	Colon-Rectum	-0.09	-5.17	4.99
V2	Psychobiotics	Female	No	No	Colon-Rectum	27.43	22.22	32.63
V2	Placebo	Female	No	No	Colon-Rectum	-20.15	-25.39	-14.90
V1	Psychobiotics	Male	Yes	No	Colon-Rectum	23.03	18.04	28.02
V1	Placebo	Male	Yes	No	Colon-Rectum	4.43	-0.55	9.41
V2	Psychobiotics	Male	Yes	No	Colon-Rectum	31.95	26.83	37.07
V2	Placebo	Male	Yes	No	Colon-Rectum	-15.62	-20.74	-10.51
V1	Psychobiotics	Female	Yes	No	Colon-Rectum	21.17	15.39	26.95
V1	Placebo	Female	Yes	No	Colon-Rectum	2.57	-3.20	8.34
V2	Psychobiotics	Female	Yes	No	Colon-Rectum	30.09	24.18	36.00
V2	Placebo	Female	Yes	No	Colon-Rectum	-17.48	-23.39	-11.58
V1	Psychobiotics	Male	No	Yes	Colon-Rectum	23.92	18.46	29.39
V1	Placebo	Male	No	Yes	Colon-Rectum	5.32	-0.03	10.68
V2	Psychobiotics	Male	No	Yes	Colon-Rectum	32.84	27.26	38.42
V2	Placebo	Male	No	Yes	Colon-Rectum	-14.73	-20.29	-9.18
V1	Psychobiotics	Female	No	Yes	Colon-Rectum	22.06	16.44	27.68
V1	Placebo	Female	No	Yes	Colon-Rectum	3.46	-2.05	8.98
V2	Psychobiotics	Female	No	Yes	Colon-Rectum	30.98	25.23	36.73
V2	Placebo	Female	No	Yes	Colon-Rectum	-16.59	-22.32	-10.87
V1	Psychobiotics	Male	Yes	Yes	Colon-Rectum	26.59	20.88	32.29
V1	Placebo	Male	Yes	Yes	Colon-Rectum	7.99	2.45	13.53
V2	Psychobiotics	Male	Yes	Yes	Colon-Rectum	35.51	29.71	41.30
V2	Placebo	Male	Yes	Yes	Colon-Rectum	-12.07	-17.79	-6.35
V1	Psychobiotics	Female	Yes	Yes	Colon-Rectum	24.72	18.55	30.90
V1	Placebo	Female	Yes	Yes	Colon-Rectum	6.13	0.11	12.15
V2	Psychobiotics	Female	Yes	Yes	Colon-Rectum	33.65	27.38	39.91
V2	Placebo	Female	Yes	Yes	Colon-Rectum	-13.93	-20.13	-7.73
V1	Psychobiotics	Male	No	No	Other	17.38	13.11	21.66
V1	Placebo	Male	No	No	Other	-1.21	-5.58	3.16
V2	Psychobiotics	Male	No	No	Other	26.30	21.81	30.80
V2	Placebo	Male	No	No	Other	-21.27	-25.77	-16.77
V1	Psychobiotics	Female	No	No	Other	15.52	10.72	20.33
V1	Placebo	Female	No	No	Other	-3.07	-7.96	1.82
V2	Psychobiotics	Female	No	No	Other	24.44	19.42	29.47

Visit	Group	Gender	Depression	Pain	Cancer Location	LS Mean	95% Confidence Interval	
							Lower Bound	Upper Bound
V2	Placebo	Female	No	No	Other	-23.13	-28.16	-18.10
V1	Psychobiotics	Male	Yes	No	Other	20.05	15.46	24.64
V1	Placebo	Male	Yes	No	Other	1.45	-3.15	6.05
V2	Psychobiotics	Male	Yes	No	Other	28.97	24.21	33.73
V2	Placebo	Male	Yes	No	Other	-18.61	-23.32	-13.90
V1	Psychobiotics	Female	Yes	No	Other	18.19	12.74	23.64
V1	Placebo	Female	Yes	No	Other	-0.41	-5.87	5.05
V2	Psychobiotics	Female	Yes	No	Other	27.11	21.49	32.72
V2	Placebo	Female	Yes	No	Other	-20.47	-26.04	-14.90
V1	Psychobiotics	Male	No	Yes	Other	20.94	16.17	25.71
V1	Placebo	Male	No	Yes	Other	2.34	-2.33	7.01
V2	Psychobiotics	Male	No	Yes	Other	29.86	24.92	34.80
V2	Placebo	Male	No	Yes	Other	-17.72	-22.58	-12.85
V1	Psychobiotics	Female	No	Yes	Other	19.08	14.12	24.04
V1	Placebo	Female	No	Yes	Other	0.48	-4.39	5.35
V2	Psychobiotics	Female	No	Yes	Other	28.00	22.86	33.14
V2	Placebo	Female	No	Yes	Other	-19.58	-24.65	-14.50
V1	Psychobiotics	Male	Yes	Yes	Other	23.60	18.71	28.49
V1	Placebo	Male	Yes	Yes	Other	5.00	0.28	9.73
V2	Psychobiotics	Male	Yes	Yes	Other	32.52	27.50	37.54
V2	Placebo	Male	Yes	Yes	Other	-15.05	-19.95	-10.15
V1	Psychobiotics	Female	Yes	Yes	Other	21.74	16.30	27.18
V1	Placebo	Female	Yes	Yes	Other	3.14	-2.15	8.44
V2	Psychobiotics	Female	Yes	Yes	Other	30.66	25.09	36.24
V2	Placebo	Female	Yes	Yes	Other	-16.91	-22.38	-11.45

LS=Least Squares

Estimates are adjusted for visit, group, baseline role physical, gender, depression status, pain status, and cancer location, including visit-by-group and visit-by-baseline role physical interactions.

Reference Table 2.2S

Table S3.3 Bodily Pain [BP]: Least Squares Mean Estimates for Change from Baseline by Visit and Group Across Covariate Levels

							95% Confidence Interval	
Visit	Group	Gender	Depression	Pain	Cancer Location	LS Mean	Lower Bound	Upper Bound
V1	Psychobiotics	Male	No	No	Colon-Rectum	7.25	3.71	10.79
V1	Placebo	Male	No	No	Colon-Rectum	1.95	-1.60	5.49
V2	Psychobiotics	Male	No	No	Colon-Rectum	17.50	13.88	21.12
V2	Placebo	Male	No	No	Colon-Rectum	-10.55	-14.17	-6.92
V1	Psychobiotics	Female	No	No	Colon-Rectum	10.31	6.33	14.29
V1	Placebo	Female	No	No	Colon-Rectum	5.01	1.02	9.00
V2	Psychobiotics	Female	No	No	Colon-Rectum	20.56	16.49	24.64
V2	Placebo	Female	No	No	Colon-Rectum	-7.48	-11.55	-3.41
V1	Psychobiotics	Male	Yes	No	Colon-Rectum	9.76	5.87	13.65
V1	Placebo	Male	Yes	No	Colon-Rectum	4.46	0.57	8.36
V2	Psychobiotics	Male	Yes	No	Colon-Rectum	20.02	16.07	23.97
V2	Placebo	Male	Yes	No	Colon-Rectum	-8.03	-12.00	-4.06
V1	Psychobiotics	Female	Yes	No	Colon-Rectum	12.83	8.28	17.37
V1	Placebo	Female	Yes	No	Colon-Rectum	7.53	2.97	12.08
V2	Psychobiotics	Female	Yes	No	Colon-Rectum	23.08	18.47	27.69
V2	Placebo	Female	Yes	No	Colon-Rectum	-4.97	-9.59	-0.34
V1	Psychobiotics	Male	No	Yes	Colon-Rectum	5.63	1.32	9.94
V1	Placebo	Male	No	Yes	Colon-Rectum	0.33	-3.91	4.57
V2	Psychobiotics	Male	No	Yes	Colon-Rectum	15.88	11.52	20.24
V2	Placebo	Male	No	Yes	Colon-Rectum	-12.16	-16.52	-7.81
V1	Psychobiotics	Female	No	Yes	Colon-Rectum	8.69	4.26	13.12
V1	Placebo	Female	No	Yes	Colon-Rectum	3.39	-0.98	7.76
V2	Psychobiotics	Female	No	Yes	Colon-Rectum	18.95	14.45	23.44
V2	Placebo	Female	No	Yes	Colon-Rectum	-9.10	-13.58	-4.62
V1	Psychobiotics	Male	Yes	Yes	Colon-Rectum	8.14	3.65	12.64
V1	Placebo	Male	Yes	Yes	Colon-Rectum	2.84	-1.59	7.28
V2	Psychobiotics	Male	Yes	Yes	Colon-Rectum	18.40	13.87	22.93
V2	Placebo	Male	Yes	Yes	Colon-Rectum	-9.65	-14.19	-5.11
V1	Psychobiotics	Female	Yes	Yes	Colon-Rectum	11.21	6.36	16.06
V1	Placebo	Female	Yes	Yes	Colon-Rectum	5.91	1.11	10.70
V2	Psychobiotics	Female	Yes	Yes	Colon-Rectum	21.46	16.57	26.36
V2	Placebo	Female	Yes	Yes	Colon-Rectum	-6.59	-11.48	-1.69
V1	Psychobiotics	Male	No	No	Other	8.73	5.42	12.03
V1	Placebo	Male	No	No	Other	3.43	0.09	6.76
V2	Psychobiotics	Male	No	No	Other	18.98	15.56	22.40
V2	Placebo	Male	No	No	Other	-9.07	-12.47	-5.67
V1	Psychobiotics	Female	No	No	Other	11.79	8.00	15.58
V1	Placebo	Female	No	No	Other	6.49	2.67	10.30
V2	Psychobiotics	Female	No	No	Other	22.04	18.13	25.95
V2	Placebo	Female	No	No	Other	-6.00	-9.88	-2.12
V1	Psychobiotics	Male	Yes	No	Other	11.24	7.69	14.80
V1	Placebo	Male	Yes	No	Other	5.94	2.36	9.52
V2	Psychobiotics	Male	Yes	No	Other	21.50	17.85	25.14

Visit	Group	Gender	Depression	Pain	Cancer Location	LS Mean	95% Confidence Interval	
							Lower Bound	Upper Bound
V2	Placebo	Male	Yes	No	Other	-6.55	-10.20	-2.91
V1	Psychobiotics	Female	Yes	No	Other	14.31	10.03	18.58
V1	Placebo	Female	Yes	No	Other	9.00	4.70	13.31
V2	Psychobiotics	Female	Yes	No	Other	24.56	20.19	28.93
V2	Placebo	Female	Yes	No	Other	-3.49	-7.85	0.87
V1	Psychobiotics	Male	No	Yes	Other	7.11	3.40	10.82
V1	Placebo	Male	No	Yes	Other	1.81	-1.84	5.45
V2	Psychobiotics	Male	No	Yes	Other	17.36	13.57	21.15
V2	Placebo	Male	No	Yes	Other	-10.69	-14.44	-6.93
V1	Psychobiotics	Female	No	Yes	Other	10.17	6.30	14.04
V1	Placebo	Female	No	Yes	Other	4.87	1.06	8.68
V2	Psychobiotics	Female	No	Yes	Other	20.42	16.46	24.39
V2	Placebo	Female	No	Yes	Other	-7.62	-11.54	-3.70
V1	Psychobiotics	Male	Yes	Yes	Other	9.62	5.81	13.43
V1	Placebo	Male	Yes	Yes	Other	4.32	0.57	8.07
V2	Psychobiotics	Male	Yes	Yes	Other	19.88	16.00	23.75
V2	Placebo	Male	Yes	Yes	Other	-8.17	-12.03	-4.31
V1	Psychobiotics	Female	Yes	Yes	Other	12.69	8.45	16.92
V1	Placebo	Female	Yes	Yes	Other	7.39	3.20	11.57
V2	Psychobiotics	Female	Yes	Yes	Other	22.94	18.63	27.25
V2	Placebo	Female	Yes	Yes	Other	-5.11	-9.39	-0.82

LS=Least Square

Estimates are adjusted for visit, group, baseline BP, gender, depression status, pain status, and cancer location, including visit-by-group and visit-by-baseline BP interactions.

Reference Table 3.2S

Table S3.4. General Health [GH]: Least Squares Mean Estimates for Change from Baseline by Visit and Group Across Covariate Levels

							95% Confidence Interval	
Visit	Group	Gender	Depression	Pain	Cancer Location	LS Mean	Lower Bound	Upper Bound
V1	Psychobiotics	Male	No	No	Colon-Rectum	7.14	4.95	9.33
V1	Placebo	Male	No	No	Colon-Rectum	-0.93	-3.15	1.29
V2	Psychobiotics	Male	No	No	Colon-Rectum	24.05	21.82	26.29
V2	Placebo	Male	No	No	Colon-Rectum	-14.37	-16.63	-12.10
V1	Psychobiotics	Female	No	No	Colon-Rectum	7.44	4.99	9.90
V1	Placebo	Female	No	No	Colon-Rectum	-0.63	-3.12	1.87
V2	Psychobiotics	Female	No	No	Colon-Rectum	24.36	21.86	26.86
V2	Placebo	Female	No	No	Colon-Rectum	-14.06	-16.61	-11.52
V1	Psychobiotics	Male	Yes	No	Colon-Rectum	8.57	6.13	11.02
V1	Placebo	Male	Yes	No	Colon-Rectum	0.50	-1.95	2.95
V2	Psychobiotics	Male	Yes	No	Colon-Rectum	25.48	23.01	27.96
V2	Placebo	Male	Yes	No	Colon-Rectum	-12.94	-15.42	-10.45
V1	Psychobiotics	Female	Yes	No	Colon-Rectum	8.88	6.03	11.72
V1	Placebo	Female	Yes	No	Colon-Rectum	0.81	-2.06	3.67
V2	Psychobiotics	Female	Yes	No	Colon-Rectum	25.79	22.91	28.67
V2	Placebo	Female	Yes	No	Colon-Rectum	-12.63	-15.53	-9.73
V1	Psychobiotics	Male	No	Yes	Colon-Rectum	8.35	5.64	11.06
V1	Placebo	Male	No	Yes	Colon-Rectum	0.28	-2.37	2.93
V2	Psychobiotics	Male	No	Yes	Colon-Rectum	25.26	22.52	28.00
V2	Placebo	Male	No	Yes	Colon-Rectum	-13.16	-15.86	-10.46
V1	Psychobiotics	Female	No	Yes	Colon-Rectum	8.65	5.86	11.45
V1	Placebo	Female	No	Yes	Colon-Rectum	0.58	-2.17	3.33
V2	Psychobiotics	Female	No	Yes	Colon-Rectum	25.56	22.74	28.39
V2	Placebo	Female	No	Yes	Colon-Rectum	-12.86	-15.66	-10.05
V1	Psychobiotics	Male	Yes	Yes	Colon-Rectum	9.78	6.96	12.60
V1	Placebo	Male	Yes	Yes	Colon-Rectum	1.71	-1.03	4.45
V2	Psychobiotics	Male	Yes	Yes	Colon-Rectum	26.69	23.85	29.53
V2	Placebo	Male	Yes	Yes	Colon-Rectum	-11.73	-14.52	-8.94
V1	Psychobiotics	Female	Yes	Yes	Colon-Rectum	10.09	7.04	13.13
V1	Placebo	Female	Yes	Yes	Colon-Rectum	2.01	-0.98	5.00
V2	Psychobiotics	Female	Yes	Yes	Colon-Rectum	27.00	23.92	30.07
V2	Placebo	Female	Yes	Yes	Colon-Rectum	-11.42	-14.46	-8.38
V1	Psychobiotics	Male	No	No	Other	7.28	5.24	9.32
V1	Placebo	Male	No	No	Other	-0.79	-2.87	1.29
V2	Psychobiotics	Male	No	No	Other	24.19	22.10	26.29
V2	Placebo	Male	No	No	Other	-14.23	-16.34	-12.11
V1	Psychobiotics	Female	No	No	Other	7.59	5.25	9.92
V1	Placebo	Female	No	No	Other	-0.49	-2.87	1.90
V2	Psychobiotics	Female	No	No	Other	24.50	22.11	26.89
V2	Placebo	Female	No	No	Other	-13.92	-16.34	-11.50
V1	Psychobiotics	Male	Yes	No	Other	8.71	6.49	10.94
V1	Placebo	Male	Yes	No	Other	0.64	-1.60	2.88
V2	Psychobiotics	Male	Yes	No	Other	25.63	23.35	27.90

							95% Confidence Interval	
Visit	Group	Gender	Depression	Pain	Cancer Location	LS Mean	Lower Bound	Upper Bound
V2	Placebo	Male	Yes	No	Other	-12.79	-15.07	-10.52
V1	Psychobiotics	Female	Yes	No	Other	9.02	6.35	11.69
V1	Placebo	Female	Yes	No	Other	0.95	-1.75	3.65
V2	Psychobiotics	Female	Yes	No	Other	25.93	23.21	28.65
V2	Placebo	Female	Yes	No	Other	-12.49	-15.22	-9.76
V1	Psychobiotics	Male	No	Yes	Other	8.49	6.15	10.83
V1	Placebo	Male	No	Yes	Other	0.42	-1.86	2.69
V2	Psychobiotics	Male	No	Yes	Other	25.40	23.02	27.79
V2	Placebo	Male	No	Yes	Other	-13.02	-15.34	-10.69
V1	Psychobiotics	Female	No	Yes	Other	8.79	6.35	11.24
V1	Placebo	Female	No	Yes	Other	0.72	-1.68	3.12
V2	Psychobiotics	Female	No	Yes	Other	25.71	23.21	28.20
V2	Placebo	Female	No	Yes	Other	-12.71	-15.17	-10.26
V1	Psychobiotics	Male	Yes	Yes	Other	9.92	7.54	12.31
V1	Placebo	Male	Yes	Yes	Other	1.85	-0.45	4.15
V2	Psychobiotics	Male	Yes	Yes	Other	26.83	24.41	29.26
V2	Placebo	Male	Yes	Yes	Other	-11.59	-13.94	-9.24
V1	Psychobiotics	Female	Yes	Yes	Other	10.23	7.56	12.89
V1	Placebo	Female	Yes	Yes	Other	2.15	-0.45	4.76
V2	Psychobiotics	Female	Yes	Yes	Other	27.14	24.43	29.84
V2	Placebo	Female	Yes	Yes	Other	-11.28	-13.94	-8.63

LS=Least Square

Estimates are adjusted for visit, group, baseline GH, gender, depression status, pain status, and cancer location, including visit-by-group and visit-by-baseline GH interactions.

Reference Table 4.2S

Table S3.5. Vitality [VT]: Least Squares Mean Estimates for Change from Baseline by Visit and Group Across Covariate Levels

							95% Confidence Interval	
Visit	Group	Gender	Depression	Pain	Cancer Location	LS Mean	Lower Bound	Upper Bound
V1	Psychobiotics	Male	No	No	Colon-Rectum	8.49	5.93	11.04
V1	Placebo	Male	No	No	Colon-Rectum	7.99	5.40	10.59
V2	Psychobiotics	Male	No	No	Colon-Rectum	11.38	8.75	14.00
V2	Placebo	Male	No	No	Colon-Rectum	-5.92	-8.58	-3.26
V1	Psychobiotics	Female	No	No	Colon-Rectum	6.90	4.01	9.79
V1	Placebo	Female	No	No	Colon-Rectum	6.41	3.53	9.28
V2	Psychobiotics	Female	No	No	Colon-Rectum	9.79	6.83	12.75
V2	Placebo	Female	No	No	Colon-Rectum	-7.51	-10.44	-4.57
V1	Psychobiotics	Male	Yes	No	Colon-Rectum	13.14	10.34	15.94
V1	Placebo	Male	Yes	No	Colon-Rectum	12.65	9.80	15.50
V2	Psychobiotics	Male	Yes	No	Colon-Rectum	16.03	13.17	18.89
V2	Placebo	Male	Yes	No	Colon-Rectum	-1.27	-4.16	1.63
V1	Psychobiotics	Female	Yes	No	Colon-Rectum	11.55	8.26	14.84
V1	Placebo	Female	Yes	No	Colon-Rectum	11.06	7.78	14.34
V2	Psychobiotics	Female	Yes	No	Colon-Rectum	14.44	11.10	17.79
V2	Placebo	Female	Yes	No	Colon-Rectum	-2.85	-6.18	0.48
V1	Psychobiotics	Male	No	Yes	Colon-Rectum	6.76	3.62	9.90
V1	Placebo	Male	No	Yes	Colon-Rectum	6.27	3.23	9.31
V2	Psychobiotics	Male	No	Yes	Colon-Rectum	9.65	6.46	12.84
V2	Placebo	Male	No	Yes	Colon-Rectum	-7.64	-10.76	-4.53
V1	Psychobiotics	Female	No	Yes	Colon-Rectum	5.17	1.90	8.45
V1	Placebo	Female	No	Yes	Colon-Rectum	4.68	1.56	7.80
V2	Psychobiotics	Female	No	Yes	Colon-Rectum	8.07	4.74	11.39
V2	Placebo	Female	No	Yes	Colon-Rectum	-9.23	-12.43	-6.03
V1	Psychobiotics	Male	Yes	Yes	Colon-Rectum	11.42	8.16	14.67
V1	Placebo	Male	Yes	Yes	Colon-Rectum	10.92	7.77	14.08
V2	Psychobiotics	Male	Yes	Yes	Colon-Rectum	14.31	11.01	17.60
V2	Placebo	Male	Yes	Yes	Colon-Rectum	-2.99	-6.21	0.23
V1	Psychobiotics	Female	Yes	Yes	Colon-Rectum	9.83	6.28	13.37
V1	Placebo	Female	Yes	Yes	Colon-Rectum	9.34	5.92	12.75
V2	Psychobiotics	Female	Yes	Yes	Colon-Rectum	12.72	9.13	16.31
V2	Placebo	Female	Yes	Yes	Colon-Rectum	-4.58	-8.06	-1.10
V1	Psychobiotics	Male	No	No	Other	10.24	7.86	12.61
V1	Placebo	Male	No	No	Other	9.74	7.28	12.21
V2	Psychobiotics	Male	No	No	Other	13.13	10.66	15.59
V2	Placebo	Male	No	No	Other	-4.17	-6.69	-1.65
V1	Psychobiotics	Female	No	No	Other	8.65	5.91	11.38
V1	Placebo	Female	No	No	Other	8.15	5.39	10.92
V2	Psychobiotics	Female	No	No	Other	11.54	8.72	14.36
V2	Placebo	Female	No	No	Other	-5.76	-8.57	-2.94
V1	Psychobiotics	Male	Yes	No	Other	14.89	12.33	17.45
V1	Placebo	Male	Yes	No	Other	14.40	11.74	17.05
V2	Psychobiotics	Male	Yes	No	Other	17.78	15.14	20.41
V2	Placebo	Male	Yes	No	Other	0.48	-2.21	3.18
V1	Psychobiotics	Female	Yes	No	Other	13.30	10.21	16.39
V1	Placebo	Female	Yes	No	Other	12.81	9.69	15.92
V2	Psychobiotics	Female	Yes	No	Other	16.19	13.03	19.35
V2	Placebo	Female	Yes	No	Other	-1.10	-4.26	2.05

Visit	Group	Gender	Depression	Pain	Cancer Location	LS Mean	95% Confidence Interval	
							Lower Bound	Upper Bound
V1	Psychobiotics	Male	No	Yes	Other	8.51	5.81	11.22
V1	Placebo	Male	No	Yes	Other	8.02	5.39	10.64
V2	Psychobiotics	Male	No	Yes	Other	11.40	8.63	14.18
V2	Placebo	Male	No	Yes	Other	-5.89	-8.60	-3.19
V1	Psychobiotics	Female	No	Yes	Other	6.92	4.06	9.78
V1	Placebo	Female	No	Yes	Other	6.43	3.70	9.16
V2	Psychobiotics	Female	No	Yes	Other	9.81	6.88	12.75
V2	Placebo	Female	No	Yes	Other	-7.48	-10.29	-4.67
V1	Psychobiotics	Male	Yes	Yes	Other	13.16	10.41	15.92
V1	Placebo	Male	Yes	Yes	Other	12.67	9.99	15.36
V2	Psychobiotics	Male	Yes	Yes	Other	16.06	13.24	18.87
V2	Placebo	Male	Yes	Yes	Other	-1.24	-3.99	1.51
V1	Psychobiotics	Female	Yes	Yes	Other	11.58	8.48	14.68
V1	Placebo	Female	Yes	Yes	Other	11.08	8.10	14.07
V2	Psychobiotics	Female	Yes	Yes	Other	14.47	11.30	17.63
V2	Placebo	Female	Yes	Yes	Other	-2.83	-5.88	0.22

LS=Least Square

Estimates are adjusted for visit, group, baseline vitality, gender, depression status, pain status, and cancer location, including visit-by-group and visit-by-baseline vitality interactions.

Reference Table 5.2S

Table S3.6. Social Functioning [SF]: Least Squares Mean Estimates for Change from Baseline by Visit and Group Across Covariate Levels

							95% Confidence Interval	
Visit	Group	Gender	Depression	Pain	Cancer Location	LS Mean	Lower Bound	Upper Bound
V1	Psychobiotics	Male	No	No	Colon-Rectum	8.96	6.02	11.90
V1	Placebo	Male	No	No	Colon-Rectum	-0.31	-3.30	2.68
V2	Psychobiotics	Male	No	No	Colon-Rectum	24.20	21.18	27.22
V2	Placebo	Male	No	No	Colon-Rectum	-13.44	-16.51	-10.38
V1	Psychobiotics	Female	No	No	Colon-Rectum	14.14	10.85	17.43
V1	Placebo	Female	No	No	Colon-Rectum	4.86	1.50	8.23
V2	Psychobiotics	Female	No	No	Colon-Rectum	29.38	26.01	32.74
V2	Placebo	Female	No	No	Colon-Rectum	-8.27	-11.71	-4.83
V1	Psychobiotics	Male	Yes	No	Colon-Rectum	8.59	5.35	11.83
V1	Placebo	Male	Yes	No	Colon-Rectum	-0.68	-3.92	2.56
V2	Psychobiotics	Male	Yes	No	Colon-Rectum	23.83	20.52	27.14
V2	Placebo	Male	Yes	No	Colon-Rectum	-13.82	-17.12	-10.51
V1	Psychobiotics	Female	Yes	No	Colon-Rectum	13.77	10.00	17.53
V1	Placebo	Female	Yes	No	Colon-Rectum	4.49	0.71	8.28
V2	Psychobiotics	Female	Yes	No	Colon-Rectum	29.01	25.18	32.83
V2	Placebo	Female	Yes	No	Colon-Rectum	-8.64	-12.49	-4.79
V1	Psychobiotics	Male	No	Yes	Colon-Rectum	10.18	6.60	13.77
V1	Placebo	Male	No	Yes	Colon-Rectum	0.91	-2.60	4.43
V2	Psychobiotics	Male	No	Yes	Colon-Rectum	25.42	21.78	29.07
V2	Placebo	Male	No	Yes	Colon-Rectum	-12.22	-15.83	-8.61
V1	Psychobiotics	Female	No	Yes	Colon-Rectum	15.36	11.67	19.05
V1	Placebo	Female	No	Yes	Colon-Rectum	6.09	2.45	9.73
V2	Psychobiotics	Female	No	Yes	Colon-Rectum	30.60	26.85	34.35
V2	Placebo	Female	No	Yes	Colon-Rectum	-7.04	-10.79	-3.30
V1	Psychobiotics	Male	Yes	Yes	Colon-Rectum	9.81	6.06	13.57
V1	Placebo	Male	Yes	Yes	Colon-Rectum	0.54	-3.10	4.18
V2	Psychobiotics	Male	Yes	Yes	Colon-Rectum	25.05	21.25	28.86
V2	Placebo	Male	Yes	Yes	Colon-Rectum	-12.59	-16.32	-8.87
V1	Psychobiotics	Female	Yes	Yes	Colon-Rectum	14.99	10.95	19.03
V1	Placebo	Female	Yes	Yes	Colon-Rectum	5.72	1.76	9.67
V2	Psychobiotics	Female	Yes	Yes	Colon-Rectum	30.23	26.14	34.32
V2	Placebo	Female	Yes	Yes	Colon-Rectum	-7.41	-11.46	-3.37
V1	Psychobiotics	Male	No	No	Other	5.78	3.05	8.51
V1	Placebo	Male	No	No	Other	-3.49	-6.28	-0.70
V2	Psychobiotics	Male	No	No	Other	21.02	18.18	23.86
V2	Placebo	Male	No	No	Other	-16.63	-19.48	-13.77
V1	Psychobiotics	Female	No	No	Other	10.96	7.84	14.07
V1	Placebo	Female	No	No	Other	1.68	-1.51	4.88
V2	Psychobiotics	Female	No	No	Other	26.19	22.98	29.41
V2	Placebo	Female	No	No	Other	-11.45	-14.71	-8.19
V1	Psychobiotics	Male	Yes	No	Other	5.41	2.42	8.40
V1	Placebo	Male	Yes	No	Other	-3.86	-6.86	-0.87
V2	Psychobiotics	Male	Yes	No	Other	20.65	17.56	23.73

Visit	Group	Gender	Depression	Pain	Cancer Location	LS Mean	95% Confidence Interval	
							Lower Bound	Upper Bound
V2	Placebo	Male	Yes	No	Other	-17.00	-20.04	-13.95
V1	Psychobiotics	Female	Yes	No	Other	10.59	7.02	14.15
V1	Placebo	Female	Yes	No	Other	1.31	-2.27	4.90
V2	Psychobiotics	Female	Yes	No	Other	25.82	22.18	29.47
V2	Placebo	Female	Yes	No	Other	-11.82	-15.46	-8.18
V1	Psychobiotics	Male	No	Yes	Other	7.00	3.91	10.09
V1	Placebo	Male	No	Yes	Other	-2.27	-5.28	0.74
V2	Psychobiotics	Male	No	Yes	Other	22.24	19.07	25.42
V2	Placebo	Male	No	Yes	Other	-15.40	-18.50	-12.31
V1	Psychobiotics	Female	No	Yes	Other	12.18	8.96	15.40
V1	Placebo	Female	No	Yes	Other	2.91	-0.26	6.07
V2	Psychobiotics	Female	No	Yes	Other	27.42	24.11	30.73
V2	Placebo	Female	No	Yes	Other	-10.22	-13.49	-6.96
V1	Psychobiotics	Male	Yes	Yes	Other	6.63	3.41	9.86
V1	Placebo	Male	Yes	Yes	Other	-2.64	-5.73	0.45
V2	Psychobiotics	Male	Yes	Yes	Other	21.87	18.57	25.17
V2	Placebo	Male	Yes	Yes	Other	-15.77	-18.94	-12.60
V1	Psychobiotics	Female	Yes	Yes	Other	11.81	8.25	15.37
V1	Placebo	Female	Yes	Yes	Other	2.54	-0.93	6.00
V2	Psychobiotics	Female	Yes	Yes	Other	27.05	23.41	30.68
V2	Placebo	Female	Yes	Yes	Other	-10.59	-14.14	-7.05

LS=Least Square

Estimates are adjusted for visit, group, baseline SF, gender, depression status, pain status, and cancer location, including visit-by-group and visit-by-baseline SF interactions.

Reference Table 6.2S

Table S3.7. Role Emotional [RE]: Least Squares Mean Estimates for Change from Baseline by Visit and Group Across Covariate Levels

							95% Confidence Interval	
Visit	Group	Gender	Depression	Pain	Cancer Location	LS Mean	Lower Bound	Upper Bound
V1	Psychobiotics	Male	No	No	Colon-Rectum	15.42	10.43	20.40
V1	Placebo	Male	No	No	Colon-Rectum	-1.75	-6.78	3.28
V2	Psychobiotics	Male	No	No	Colon-Rectum	26.63	21.44	31.82
V2	Placebo	Male	No	No	Colon-Rectum	-18.81	-24.02	-13.60
V1	Psychobiotics	Female	No	No	Colon-Rectum	14.00	8.52	19.48
V1	Placebo	Female	No	No	Colon-Rectum	-3.17	-8.71	2.37
V2	Psychobiotics	Female	No	No	Colon-Rectum	25.21	19.52	30.90
V2	Placebo	Female	No	No	Colon-Rectum	-20.23	-25.96	-14.49
V1	Psychobiotics	Male	Yes	No	Colon-Rectum	21.61	16.17	27.06
V1	Placebo	Male	Yes	No	Colon-Rectum	4.45	-0.99	9.89
V2	Psychobiotics	Male	Yes	No	Colon-Rectum	32.82	27.22	38.42
V2	Placebo	Male	Yes	No	Colon-Rectum	-12.61	-18.21	-7.01
V1	Psychobiotics	Female	Yes	No	Colon-Rectum	20.20	13.97	26.42
V1	Placebo	Female	Yes	No	Colon-Rectum	3.03	-3.22	9.27
V2	Psychobiotics	Female	Yes	No	Colon-Rectum	31.41	25.02	37.79
V2	Placebo	Female	Yes	No	Colon-Rectum	-14.03	-20.44	-7.62
V1	Psychobiotics	Male	No	Yes	Colon-Rectum	16.14	10.17	22.12
V1	Placebo	Male	No	Yes	Colon-Rectum	-1.03	-6.85	4.80
V2	Psychobiotics	Male	No	Yes	Colon-Rectum	27.35	21.24	33.46
V2	Placebo	Male	No	Yes	Colon-Rectum	-18.08	-24.14	-12.02
V1	Psychobiotics	Female	No	Yes	Colon-Rectum	14.73	8.60	20.85
V1	Placebo	Female	No	Yes	Colon-Rectum	-2.44	-8.46	3.57
V2	Psychobiotics	Female	No	Yes	Colon-Rectum	25.93	19.65	32.22
V2	Placebo	Female	No	Yes	Colon-Rectum	-19.50	-25.76	-13.24
V1	Psychobiotics	Male	Yes	Yes	Colon-Rectum	22.34	16.10	28.58
V1	Placebo	Male	Yes	Yes	Colon-Rectum	5.17	-0.89	11.24
V2	Psychobiotics	Male	Yes	Yes	Colon-Rectum	33.55	27.20	39.90
V2	Placebo	Male	Yes	Yes	Colon-Rectum	-11.88	-18.17	-5.60
V1	Psychobiotics	Female	Yes	Yes	Colon-Rectum	20.92	14.23	27.62
V1	Placebo	Female	Yes	Yes	Colon-Rectum	3.75	-2.79	10.30
V2	Psychobiotics	Female	Yes	Yes	Colon-Rectum	32.13	25.32	38.94
V2	Placebo	Female	Yes	Yes	Colon-Rectum	-13.30	-20.08	-6.53
V1	Psychobiotics	Male	No	No	Other	12.65	7.93	17.37
V1	Placebo	Male	No	No	Other	-4.52	-9.33	0.29
V2	Psychobiotics	Male	No	No	Other	23.86	18.88	28.84
V2	Placebo	Male	No	No	Other	-21.57	-26.52	-16.62
V1	Psychobiotics	Female	No	No	Other	11.23	5.97	16.50
V1	Placebo	Female	No	No	Other	-5.94	-11.30	-0.57
V2	Psychobiotics	Female	No	No	Other	22.44	16.92	27.97
V2	Placebo	Female	No	No	Other	-22.99	-28.51	-17.47
V1	Psychobiotics	Male	Yes	No	Other	18.85	13.83	23.87
V1	Placebo	Male	Yes	No	Other	1.68	-3.37	6.73
V2	Psychobiotics	Male	Yes	No	Other	30.06	24.83	35.29
V2	Placebo	Male	Yes	No	Other	-15.38	-20.55	-10.20

							95% Confidence Interval	
Visit	Group	Gender	Depression	Pain	Cancer Location	LS Mean	Lower Bound	Upper Bound
V1	Psychobiotics	Female	Yes	No	Other	17.43	11.55	23.31
V1	Placebo	Female	Yes	No	Other	0.26	-5.67	6.19
V2	Psychobiotics	Female	Yes	No	Other	28.64	22.56	34.72
V2	Placebo	Female	Yes	No	Other	-16.79	-22.86	-10.73
V1	Psychobiotics	Male	No	Yes	Other	13.38	8.17	18.59
V1	Placebo	Male	No	Yes	Other	-3.79	-8.88	1.29
V2	Psychobiotics	Male	No	Yes	Other	24.59	19.17	30.00
V2	Placebo	Male	No	Yes	Other	-20.85	-26.15	-15.54
V1	Psychobiotics	Female	No	Yes	Other	11.96	6.55	17.37
V1	Placebo	Female	No	Yes	Other	-5.21	-10.53	0.11
V2	Psychobiotics	Female	No	Yes	Other	23.17	17.54	28.80
V2	Placebo	Female	No	Yes	Other	-22.27	-27.82	-16.71
V1	Psychobiotics	Male	Yes	Yes	Other	19.58	14.23	24.92
V1	Placebo	Male	Yes	Yes	Other	2.41	-2.77	7.58
V2	Psychobiotics	Male	Yes	Yes	Other	30.78	25.28	36.29
V2	Placebo	Male	Yes	Yes	Other	-14.65	-20.03	-9.27
V1	Psychobiotics	Female	Yes	Yes	Other	18.16	12.27	24.04
V1	Placebo	Female	Yes	Yes	Other	0.99	-4.77	6.74
V2	Psychobiotics	Female	Yes	Yes	Other	29.37	23.31	35.42
V2	Placebo	Female	Yes	Yes	Other	-16.07	-22.04	-10.10

LS=Least Squares

Estimates are adjusted for visit, group, baseline role emotional, gender, depression status, pain status, and cancer location, including visit-by-group and visit-by-baseline role emotional interactions.

Reference Table 7.2S

Table S3.8. Mental Health [MH]: Least Squares Mean Estimates for Change from Baseline by Visit and Group Across Covariate Levels

Visit	Group	Gender	Depression	Pain	Cancer Location	LS Mean	95% Confidence Interval	
							Lower Bound	Upper Bound
V1	Psychobiotics	Male	No	No	Colon-Rectum	12.29	9.76	14.81
V1	Placebo	Male	No	No	Colon-Rectum	12.12	9.66	14.59
V2	Psychobiotics	Male	No	No	Colon-Rectum	15.19	12.59	17.79
V2	Placebo	Male	No	No	Colon-Rectum	-0.38	-2.89	2.13
V1	Psychobiotics	Female	No	No	Colon-Rectum	10.95	8.21	13.69
V1	Placebo	Female	No	No	Colon-Rectum	10.79	8.00	13.58
V2	Psychobiotics	Female	No	No	Colon-Rectum	13.85	11.03	16.67
V2	Placebo	Female	No	No	Colon-Rectum	-1.72	-4.56	1.12
V1	Psychobiotics	Male	Yes	No	Colon-Rectum	10.38	7.63	13.12
V1	Placebo	Male	Yes	No	Colon-Rectum	10.22	7.52	12.92
V2	Psychobiotics	Male	Yes	No	Colon-Rectum	13.28	10.48	16.09
V2	Placebo	Male	Yes	No	Colon-Rectum	-2.29	-5.04	0.45
V1	Psychobiotics	Female	Yes	No	Colon-Rectum	9.04	5.90	12.19
V1	Placebo	Female	Yes	No	Colon-Rectum	8.88	5.69	12.08
V2	Psychobiotics	Female	Yes	No	Colon-Rectum	11.95	8.75	15.15
V2	Placebo	Female	Yes	No	Colon-Rectum	-3.63	-6.86	-0.39
V1	Psychobiotics	Male	No	Yes	Colon-Rectum	12.15	9.08	15.21
V1	Placebo	Male	No	Yes	Colon-Rectum	11.99	9.07	14.90
V2	Psychobiotics	Male	No	Yes	Colon-Rectum	15.05	11.93	18.17
V2	Placebo	Male	No	Yes	Colon-Rectum	-0.52	-3.51	2.46
V1	Psychobiotics	Female	No	Yes	Colon-Rectum	10.81	7.72	13.90
V1	Placebo	Female	No	Yes	Colon-Rectum	10.65	7.61	13.69
V2	Psychobiotics	Female	No	Yes	Colon-Rectum	13.71	10.56	16.86
V2	Placebo	Female	No	Yes	Colon-Rectum	-1.86	-4.96	1.25
V1	Psychobiotics	Male	Yes	Yes	Colon-Rectum	10.24	7.08	13.40
V1	Placebo	Male	Yes	Yes	Colon-Rectum	10.08	7.05	13.11
V2	Psychobiotics	Male	Yes	Yes	Colon-Rectum	13.14	9.94	16.35
V2	Placebo	Male	Yes	Yes	Colon-Rectum	-2.43	-5.52	0.66
V1	Psychobiotics	Female	Yes	Yes	Colon-Rectum	8.90	5.54	12.27
V1	Placebo	Female	Yes	Yes	Colon-Rectum	8.74	5.41	12.07
V2	Psychobiotics	Female	Yes	Yes	Colon-Rectum	11.81	8.39	15.22
V2	Placebo	Female	Yes	Yes	Colon-Rectum	-3.77	-7.15	-0.38
V1	Psychobiotics	Male	No	No	Other	12.92	10.62	15.22
V1	Placebo	Male	No	No	Other	12.76	10.42	15.09
V2	Psychobiotics	Male	No	No	Other	15.82	13.43	18.21
V2	Placebo	Male	No	No	Other	0.25	-2.13	2.63
V1	Psychobiotics	Female	No	No	Other	11.58	9.00	14.17
V1	Placebo	Female	No	No	Other	11.42	8.69	14.15
V2	Psychobiotics	Female	No	No	Other	14.49	11.81	17.16
V2	Placebo	Female	No	No	Other	-1.09	-3.85	1.68
V1	Psychobiotics	Male	Yes	No	Other	11.01	8.54	13.48
V1	Placebo	Male	Yes	No	Other	10.85	8.33	13.37
V2	Psychobiotics	Male	Yes	No	Other	13.91	11.37	16.46

							95% Confidence Interval	
Visit	Group	Gender	Depression	Pain	Cancer Location	LS Mean	Lower Bound	Upper Bound
V2	Placebo	Male	Yes	No	Other	-1.66	-4.21	0.90
V1	Psychobiotics	Female	Yes	No	Other	9.67	6.73	12.62
V1	Placebo	Female	Yes	No	Other	9.51	6.43	12.60
V2	Psychobiotics	Female	Yes	No	Other	12.58	9.57	15.59
V2	Placebo	Female	Yes	No	Other	-2.99	-6.11	0.12
V1	Psychobiotics	Male	No	Yes	Other	12.78	10.17	15.38
V1	Placebo	Male	No	Yes	Other	12.62	10.10	15.14
V2	Psychobiotics	Male	No	Yes	Other	15.68	13.01	18.35
V2	Placebo	Male	No	Yes	Other	0.11	-2.48	2.69
V1	Psychobiotics	Female	No	Yes	Other	11.44	8.76	14.12
V1	Placebo	Female	No	Yes	Other	11.28	8.57	13.99
V2	Psychobiotics	Female	No	Yes	Other	14.35	11.60	17.10
V2	Placebo	Female	No	Yes	Other	-1.23	-3.99	1.54
V1	Psychobiotics	Male	Yes	Yes	Other	10.87	8.22	13.52
V1	Placebo	Male	Yes	Yes	Other	10.71	8.13	13.30
V2	Psychobiotics	Male	Yes	Yes	Other	13.78	11.07	16.48
V2	Placebo	Male	Yes	Yes	Other	-1.80	-4.44	0.84
V1	Psychobiotics	Female	Yes	Yes	Other	9.54	6.60	12.47
V1	Placebo	Female	Yes	Yes	Other	9.38	6.41	12.34
V2	Psychobiotics	Female	Yes	Yes	Other	12.44	9.45	15.43
V2	Placebo	Female	Yes	Yes	Other	-3.13	-6.16	-0.11

LS=Least Square

Estimates are adjusted for visit, group, baseline EWB, gender, depression status, pain status, and cancer location, including visit-by-group and visit-by-baseline EWB interactions.

Reference Table 8.2S

Table S3.9. Physical Component Summary [PCS]: Least Squares Mean Estimates for Change from Baseline by Visit and Group Across Covariate Levels

							95% Confidence Interval	
Visit	Group	Gender	Depression	Pain	Cancer Location	LS Mean	Lower Bound	Upper Bound
V1	Psychobiotics	Male	No	No	Colon-Rectum	3.08	2.10	4.06
V1	Placebo	Male	No	No	Colon-Rectum	-1.88	-2.87	-0.88
V2	Psychobiotics	Male	No	No	Colon-Rectum	8.28	7.26	9.30
V2	Placebo	Male	No	No	Colon-Rectum	-8.51	-9.54	-7.48
V1	Psychobiotics	Female	No	No	Colon-Rectum	3.63	2.55	4.71
V1	Placebo	Female	No	No	Colon-Rectum	-1.33	-2.44	-0.22
V2	Psychobiotics	Female	No	No	Colon-Rectum	8.82	7.71	9.94
V2	Placebo	Female	No	No	Colon-Rectum	-7.96	-9.10	-6.82
V1	Psychobiotics	Male	Yes	No	Colon-Rectum	4.14	3.08	5.21
V1	Placebo	Male	Yes	No	Colon-Rectum	-0.82	-1.91	0.28
V2	Psychobiotics	Male	Yes	No	Colon-Rectum	9.34	8.24	10.44
V2	Placebo	Male	Yes	No	Colon-Rectum	-7.45	-8.57	-6.32
V1	Psychobiotics	Female	Yes	No	Colon-Rectum	4.69	3.46	5.92
V1	Placebo	Female	Yes	No	Colon-Rectum	-0.27	-1.53	1.00
V2	Psychobiotics	Female	Yes	No	Colon-Rectum	9.89	8.63	11.15
V2	Placebo	Female	Yes	No	Colon-Rectum	-6.90	-8.20	-5.60
V1	Psychobiotics	Male	No	Yes	Colon-Rectum	3.46	2.28	4.65
V1	Placebo	Male	No	Yes	Colon-Rectum	-1.50	-2.65	-0.34
V2	Psychobiotics	Male	No	Yes	Colon-Rectum	8.66	7.45	9.87
V2	Placebo	Male	No	Yes	Colon-Rectum	-8.13	-9.33	-6.93
V1	Psychobiotics	Female	No	Yes	Colon-Rectum	4.01	2.80	5.22
V1	Placebo	Female	No	Yes	Colon-Rectum	-0.95	-2.15	0.25
V2	Psychobiotics	Female	No	Yes	Colon-Rectum	9.21	7.97	10.45
V2	Placebo	Female	No	Yes	Colon-Rectum	-7.58	-8.82	-6.34
V1	Psychobiotics	Male	Yes	Yes	Colon-Rectum	4.52	3.30	5.75
V1	Placebo	Male	Yes	Yes	Colon-Rectum	-0.44	-1.65	0.78
V2	Psychobiotics	Male	Yes	Yes	Colon-Rectum	9.72	8.48	10.96
V2	Placebo	Male	Yes	Yes	Colon-Rectum	-7.07	-8.32	-5.81
V1	Psychobiotics	Female	Yes	Yes	Colon-Rectum	5.07	3.75	6.39
V1	Placebo	Female	Yes	Yes	Colon-Rectum	0.11	-1.21	1.43
V2	Psychobiotics	Female	Yes	Yes	Colon-Rectum	10.27	8.93	11.61
V2	Placebo	Female	Yes	Yes	Colon-Rectum	-6.52	-7.88	-5.16
V1	Psychobiotics	Male	No	No	Other	3.18	2.25	4.12
V1	Placebo	Male	No	No	Other	-1.78	-2.71	-0.85
V2	Psychobiotics	Male	No	No	Other	8.38	7.40	9.36
V2	Placebo	Male	No	No	Other	-8.41	-9.37	-7.45
V1	Psychobiotics	Female	No	No	Other	3.73	2.68	4.77
V1	Placebo	Female	No	No	Other	-1.23	-2.28	-0.18
V2	Psychobiotics	Female	No	No	Other	8.92	7.83	10.01
V2	Placebo	Female	No	No	Other	-7.86	-8.94	-6.78

Visit	Group	Gender	Depression	Pain	Cancer Location	LS Mean	95% Confidence Interval	
							Lower Bound	Upper Bound
V1	Psychobiotics	Male	Yes	No	Other	4.24	3.25	5.23
V1	Placebo	Male	Yes	No	Other	-0.72	-1.71	0.28
V2	Psychobiotics	Male	Yes	No	Other	9.44	8.41	10.47
V2	Placebo	Male	Yes	No	Other	-7.35	-8.37	-6.32
V1	Psychobiotics	Female	Yes	No	Other	4.79	3.62	5.96
V1	Placebo	Female	Yes	No	Other	-0.17	-1.36	1.02
V2	Psychobiotics	Female	Yes	No	Other	9.99	8.79	11.19
V2	Placebo	Female	Yes	No	Other	-6.80	-8.01	-5.59
V1	Psychobiotics	Male	No	Yes	Other	3.56	2.52	4.61
V1	Placebo	Male	No	Yes	Other	-1.40	-2.39	-0.40
V2	Psychobiotics	Male	No	Yes	Other	8.76	7.68	9.84
V2	Placebo	Male	No	Yes	Other	-8.03	-9.07	-6.99
V1	Psychobiotics	Female	No	Yes	Other	4.11	3.03	5.19
V1	Placebo	Female	No	Yes	Other	-0.85	-1.90	0.20
V2	Psychobiotics	Female	No	Yes	Other	9.30	8.19	10.42
V2	Placebo	Female	No	Yes	Other	-7.48	-8.57	-6.39
V1	Psychobiotics	Male	Yes	Yes	Other	4.62	3.57	5.68
V1	Placebo	Male	Yes	Yes	Other	-0.34	-1.36	0.69
V2	Psychobiotics	Male	Yes	Yes	Other	9.82	8.74	10.90
V2	Placebo	Male	Yes	Yes	Other	-6.97	-8.03	-5.90
V1	Psychobiotics	Female	Yes	Yes	Other	5.17	4.01	6.34
V1	Placebo	Female	Yes	Yes	Other	0.21	-0.94	1.36
V2	Psychobiotics	Female	Yes	Yes	Other	10.37	9.17	11.56
V2	Placebo	Female	Yes	Yes	Other	-6.42	-7.61	-5.23

LS=Least Squares

Estimates are adjusted for visit, group, baseline PCS, gender, depression status, pain status, and cancer location, including visit-by-group and visit-by-baseline PCS interactions.

Reference Table 9.2S

Table S3.10. Mental Component Summary [MCS]: Least Squares Mean Estimates for Change from Baseline by Visit and Group Across Covariate Levels

Visit	Group	Gender	Depression	Pain	Cancer Location	LS Mean	95% Confidence Interval Lower Bound	95% Confidence Interval Upper Bound
V1	Psychobiotics	Male	No	No	Colon-Rectum	6.00	4.91	7.09
V1	Placebo	Male	No	No	Colon-Rectum	3.84	2.75	4.94
V2	Psychobiotics	Male	No	No	Colon-Rectum	8.30	7.17	9.43
V2	Placebo	Male	No	No	Colon-Rectum	-1.36	-2.49	-0.23
V1	Psychobiotics	Female	No	No	Colon-Rectum	5.79	4.59	7.00
V1	Placebo	Female	No	No	Colon-Rectum	3.64	2.42	4.86
V2	Psychobiotics	Female	No	No	Colon-Rectum	8.09	6.85	9.34
V2	Placebo	Female	No	No	Colon-Rectum	-1.57	-2.82	-0.31
V1	Psychobiotics	Male	Yes	No	Colon-Rectum	6.33	5.12	7.53
V1	Placebo	Male	Yes	No	Colon-Rectum	4.17	2.98	5.36
V2	Psychobiotics	Male	Yes	No	Colon-Rectum	8.62	7.39	9.86
V2	Placebo	Male	Yes	No	Colon-Rectum	-1.04	-2.25	0.18
V1	Psychobiotics	Female	Yes	No	Colon-Rectum	6.12	4.73	7.50
V1	Placebo	Female	Yes	No	Colon-Rectum	3.96	2.58	5.34
V2	Psychobiotics	Female	Yes	No	Colon-Rectum	8.42	7.00	9.84
V2	Placebo	Female	Yes	No	Colon-Rectum	-1.24	-2.65	0.17
V1	Psychobiotics	Male	No	Yes	Colon-Rectum	5.97	4.64	7.29
V1	Placebo	Male	No	Yes	Colon-Rectum	3.81	2.53	5.09
V2	Psychobiotics	Male	No	Yes	Colon-Rectum	8.27	6.91	9.62
V2	Placebo	Male	No	Yes	Colon-Rectum	-1.39	-2.72	-0.07
V1	Psychobiotics	Female	No	Yes	Colon-Rectum	5.76	4.41	7.12
V1	Placebo	Female	No	Yes	Colon-Rectum	3.61	2.28	4.93
V2	Psychobiotics	Female	No	Yes	Colon-Rectum	8.06	6.67	9.45
V2	Placebo	Female	No	Yes	Colon-Rectum	-1.60	-2.97	-0.23
V1	Psychobiotics	Male	Yes	Yes	Colon-Rectum	6.29	4.90	7.69
V1	Placebo	Male	Yes	Yes	Colon-Rectum	4.14	2.81	5.47
V2	Psychobiotics	Male	Yes	Yes	Colon-Rectum	8.59	7.18	10.01
V2	Placebo	Male	Yes	Yes	Colon-Rectum	-1.07	-2.44	0.30
V1	Psychobiotics	Female	Yes	Yes	Colon-Rectum	6.09	4.59	7.58
V1	Placebo	Female	Yes	Yes	Colon-Rectum	3.93	2.49	5.37
V2	Psychobiotics	Female	Yes	Yes	Colon-Rectum	8.39	6.87	9.91
V2	Placebo	Female	Yes	Yes	Colon-Rectum	-1.27	-2.76	0.21
V1	Psychobiotics	Male	No	No	Other	5.54	4.53	6.56
V1	Placebo	Male	No	No	Other	3.39	2.34	4.44
V2	Psychobiotics	Male	No	No	Other	7.84	6.78	8.91
V2	Placebo	Male	No	No	Other	-1.82	-2.89	-0.74
V1	Psychobiotics	Female	No	No	Other	5.34	4.19	6.48
V1	Placebo	Female	No	No	Other	3.18	1.99	4.37
V2	Psychobiotics	Female	No	No	Other	7.64	6.44	8.83
V2	Placebo	Female	No	No	Other	-2.02	-3.24	-0.81
V1	Psychobiotics	Male	Yes	No	Other	5.87	4.78	6.96
V1	Placebo	Male	Yes	No	Other	3.71	2.61	4.82
V2	Psychobiotics	Male	Yes	No	Other	8.17	7.04	9.30
V2	Placebo	Male	Yes	No	Other	-1.49	-2.62	-0.37
V1	Psychobiotics	Female	Yes	No	Other	5.66	4.37	6.96

Table S3.10. Mental Component Summary [MCS]: Least Squares Mean Estimates for Change from Baseline by Visit and Group Across Covariate Levels

							95% Confidence Interval	
Visit	Group	Gender	Depression	Pain	Cancer Location	LS Mean	Lower Bound	Upper Bound
V1	Placebo	Female	Yes	No	Other	3.51	2.19	4.82
V2	Psychobiotics	Female	Yes	No	Other	7.96	6.63	9.30
V2	Placebo	Female	Yes	No	Other	-1.70	-3.03	-0.36
V1	Psychobiotics	Male	No	Yes	Other	5.51	4.37	6.65
V1	Placebo	Male	No	Yes	Other	3.36	2.24	4.47
V2	Psychobiotics	Male	No	Yes	Other	7.81	6.63	8.99
V2	Placebo	Male	No	Yes	Other	-1.85	-3.00	-0.70
V1	Psychobiotics	Female	No	Yes	Other	5.31	4.12	6.49
V1	Placebo	Female	No	Yes	Other	3.15	1.98	4.32
V2	Psychobiotics	Female	No	Yes	Other	7.61	6.38	8.83
V2	Placebo	Female	No	Yes	Other	-2.05	-3.27	-0.84
V1	Psychobiotics	Male	Yes	Yes	Other	5.84	4.66	7.01
V1	Placebo	Male	Yes	Yes	Other	3.68	2.56	4.81
V2	Psychobiotics	Male	Yes	Yes	Other	8.14	6.93	9.35
V2	Placebo	Male	Yes	Yes	Other	-1.52	-2.69	-0.36
V1	Psychobiotics	Female	Yes	Yes	Other	5.63	4.33	6.93
V1	Placebo	Female	Yes	Yes	Other	3.48	2.21	4.74
V2	Psychobiotics	Female	Yes	Yes	Other	7.93	6.60	9.26
V2	Placebo	Female	Yes	Yes	Other	-1.73	-3.03	-0.43

LS=Least Squares

Estimates are adjusted for visit, group, baseline MCS, gender, depression status, pain status, and cancer location, including visit-by-group and visit-by-baseline MCS interactions.

Reference Table 10.2

Table S3.11 Baseline SF-36 domain and component summary scores by treatment group

SF-36 outcome	Psychobiotics, mean ± SD	Placebo, mean ± SD
Physical Functioning (PF)	28.26 ± 11.71	48.88 ± 15.80
Role Physical (RP)	37.31 ± 23.42	33.21 ± 19.58
Bodily Pain (BP)	40.95 ± 21.07	48.99 ± 18.11
General Health (GH)	37.42 ± 12.02	37.01 ± 12.72
Vitality (VT)	45.38 ± 19.50	60.00 ± 13.85
Social Functioning (SF)	37.50 ± 20.95	39.74 ± 19.26
Role Emotional (RE)	36.87 ± 25.47	38.81 ± 27.48
Mental Health (MH)	47.09 ± 18.12	58.27 ± 10.48
Physical Component Summary (PCS)	31.98 ± 5.12	35.12 ± 4.83
Mental Component Summary (MCS)	38.32 ± 7.78	41.26 ± 5.53

Table S3.12. Participant numbers across categories included in the exploratory subgroup analyses.

Exploratory subgroup	Psychobiotics (n=132)	Placebo (n=134)	Total (N=266)
Gender			
Male	88	90	178
Female	44	44	88
Baseline depression status			
No	84	83	167
Yes	48	51	99
Baseline pain status			
No	87	80	167
Yes	45	54	99
Cancer location			
Colon–Rectum	89	91	180
Other	43	43	86
